# Correlating exhaled aerosol images to small airway obstructive diseases: A study with dynamic mode decomposition and machine learning

**DOI:** 10.1371/journal.pone.0211413

**Published:** 2019-01-31

**Authors:** Jinxiang Xi, Weizhong Zhao

**Affiliations:** 1 Department of Biomedical Engineering, California Baptist University, Riverside, California, United States of America; 2 Department of Aerospace and Mechanical Engineering, California Baptist University, Riverside, California, United States of America; 3 Division of Bioinformatics and Biostatistics, National Center for Toxicological Research, Jefferson, Arkansas, United States of America; Coastal Carolina University, UNITED STATES

## Abstract

**Background:**

Exhaled aerosols from lungs have unique patterns, and their variation can be correlated to the underlying lung structure and associated abnormities. However, it is challenging to characterize such aerosol patterns and differentiate their difference because of their complexity. This challenge is even greater for small airway diseases, where the disturbance signals are weak.

**Objectives and methods:**

The objective of this study is exploiting different feature extraction algorithms to develop a practical classifier to diagnose obstructive lung diseases using exhaled aerosol images. These include proper orthogonal decomposition (POD), principal component analysis (PCA), dynamic mode decomposition (DMD), and DMD with control (DMDC). Aerosol images were generated via physiology-based simulations in one normal and four diseased airway models in G7-9 bronchioles. The image data were classified using both the support vector machine (SVM) and random forest (RF) algorithms. The effectiveness of different features was evaluated by classification accuracy and misclassification rate.

**Findings:**

Results show a significantly higher performance using dynamic feature extractions (DMD and DMDC) than static algorithms (POD and PCA). Adding the control variables to DMD further improved classification accuracy. Comparing the classification methods, RF persistently outperformed SVM for all types of features considered. While the performance of RF constantly increased with the number of features retained, the performance of SVM peaked at 50 and decreased thereafter. The 5-class classification accuracy was 94.8% using the DMDC-RF model and 93.0% using the DMD-RF model, both of which were higher than 87.0% in the previous study that used fractal dimension features.

**Conclusion:**

Considering that disease progression is inherently a dynamic process, DMD(C)-based feature extraction preserves temporal information and is preferred over POD and PCA. Compared with hand-crafted features like fractals, feature extraction by DMD and DMDC is automatic and more accurate.

## Introduction

Lung diseases, either being restrictive (inhalation) such as acute respiratory distress syndrome (ARDS) and cystic fibrosis, or obstructive (exhalation) such as asthma and chronic obstructive pulmonary disease (COPD), will affect the respiratory airflow and cause a disturbance to the exhaled airflow pattern [1–3]. Exhaled aerosols can reveal a wealth of information about the health of the lungs [4]. However, there are many challenges to correlate these images to the underlying lung structural remodeling. The distributions of the exhaled aerosols are exceedingly complex, which are determined by the airflow and aerosol dynamics. Exhaled aerosol images from deep lungs generally cannot be differentiated by mere inspection. As a result, how to extract useful features from these seemingly chaotic observables is crucial in developing an effective algorithm to diagnose lung abnormalities based on exhaled aerosol images. In our previous studies [5–9], fractal-based features, such as lacunarity, fractal dimension (FD), and multifractal spectrum, have been explored for the quantification of aerosol images and subsequent machine learning of disease status. In combination with the random forest (RF) algorithm [10, 11], the optimal accuracy was predicted at 87.0% for a five-class classification of asthmatic diseases located in small airways (G8 bronchiole) [12].

Our hypothesis in this study is as follows. Instead of using FD (global or local) of the image which may suffer information loss [13, 14], aerosol patterns formed by exhaled airflows, together with their temporal dynamic processes, should better capture the progression of airway structural remodeling in deep lungs. Boser et al. [13] demonstrated that global FD could not accurately describe the asthmatic lungs and local features of the diseased region should be included. To avoid possible information loss, it is suggested that features, or eigenmodes, be extracted directly from the images (i.e., pixel values). By projecting the aerosol images onto low-dimensional eigenmodes, the underlying physics (fluid-particle transport equations) can be approximated by a dynamical system with fewer degrees of freedom, which can be used for the detection, monitoring, and when combined with targeted pulmonary drug delivery, treatment of the lung diseases.

Great advances were made in extracting features or eigenmodes from numerical simulations and experimental visualizations. Proper orthogonal decomposition (POD) [15], principal component analysis (PCA) [16], global eigenmodes [17], balanced modes [18, 19], and dynamic mode decomposition (DMD) [20, 21] have given useful insights on the dynamics of fluid flows. POD decomposes the dynamics into orthogonal modes. It provides a low-rank basis and a hierarchy of features that are most predominant in the system. PCA is equivalent to POD but removes the mean to increase the contrast. In machine learning and pattern recognition PCA has been widely applied for modal decomposition and dimensionality reduction. In recent years, DMD has attracted attention in various fields as an approach for the above purpose that works without explicit knowledge of the governing equations. Although DMD is a data-driven decomposition technique like POD and PCA, it generates modes that are directly linked with the transient dynamics of the data. In this sense, DMD is inherently suitable for studying time-evolution observables that evolve on an attractor (i.e., healthy lungs) with transient oscillators (i.e., diseases of varying severities). DMD with control (DMDC) uses both the snapshots and externally applied control to extract input-output characteristics and makes it possible to design the controller for high-dimensional, complex systems [22]. Besides analysis of fluid flow and vortex dynamics, successful applications of DMD and its variants have been made in power systems [23], robotic control [24], neuroscience [25], image processing [26], epidemiology [27], financial market [28, 29], and weather broadcasting [30]. Comprehensive reviews of the theory and applications of DMD can be found in Schmid et al. [21] and Tu et al. [31].

The objective of this study is to exploit different feature extraction algorithms in order to develop a practical classifier to diagnose obstructive lung diseases using exhaled aerosol images. There are three specific aims:

1. To explore the feature extraction algorithms such as POD, PCA, DMD, and DMDC in characterizing the exhaled aerosol images and identifying underlying dynamics.
2. To compare the classification accuracy of the extracted features in both SVM and RF.
3. To conduct sensitivity and error analysis of the proposed classifiers in detecting small structural variations.

The remaining text is organized as follows. Study design and methods of data collection, feature extraction and classification will be described in section 2. The results of physiology-based simulations, eigenmode decomposition, and ten-fold cross-validations of classification will be presented in section 3, and implication, significance, and limitations of the results will be discussed in section 4.

## Methods

### Study design

**Fig 1:**
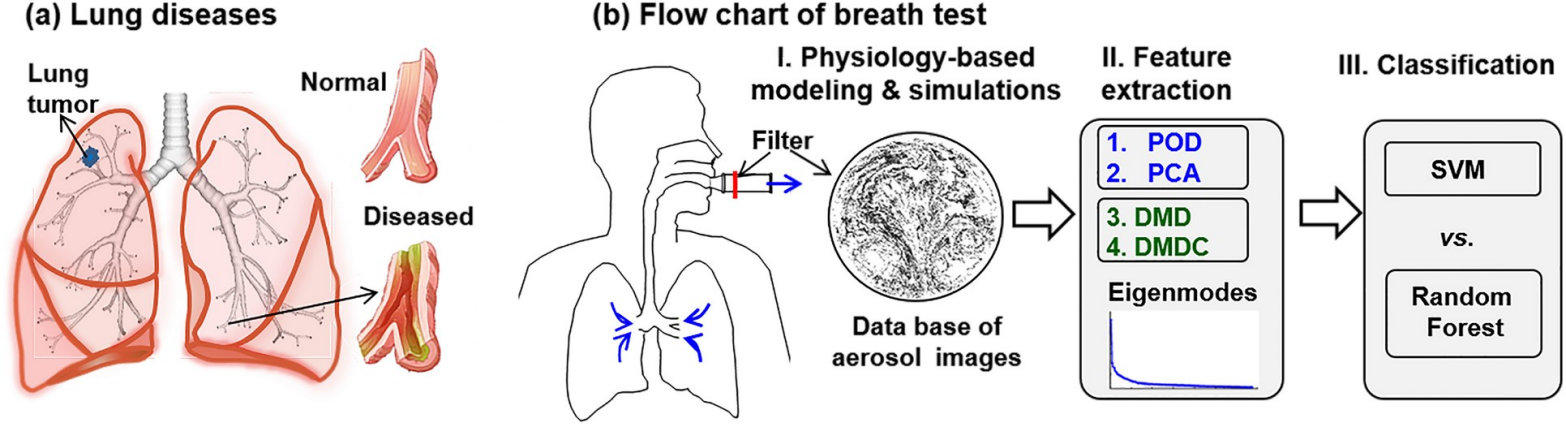
Diagram of lung diseases and study design. There are three steps in the proposed breath test: (1) physiology-based modeling and simulations to generate a database of exhaled aerosol images, (2) image feature extraction using different eigenmode decomposition algorithms, and (3) data classification (training and testing) using SVM and random forest methods.

Fig 1 depicts the workflow of this study that consists of three steps. First, a database consisting of 405 exhaled aerosol images will be generated with physiology-based modeling and simulations. The variables of interest include three respiration rates (27, 30, 33 L/min), five airway models (one normal A0, 4 diseased A1-4), nine particle sizes (0.2, 0.4, 0.6, 0.8, 1, 2, 3, 5 10 μm), and three stochastically generated inlet profiles for each particle size. An averaged inhalation flow rate of 30 L/min was chosen as the test condition in this study, which represents the light activity condition and has been often used for inhalation drug delivery in adults. To assess the uncertainties from breathing, 10% uncertainties in the inhalation flow rate (30±3 L/min) will be considered. Multiple particle sizes are considered because of their different responses to structure variations, which altogether will provide a more accurate characterization of the underlying disease. Second, Feature extraction from the aerosol images will be performed using four different eigenmode algorithms: POD, PCA, DMD, and DMDC. Thirdly, the extracted feature vectors will be deployed in two supervised machine learning methods, SVM and RF, to classify the images according to disease levels [32, 33]. A ten-fold cross-validation approach will be used for data classification and error analysis. To obtain statistically significant results, the classification will be repeated 100 times.

### Normal and diseased airway models

**Fig 2:**
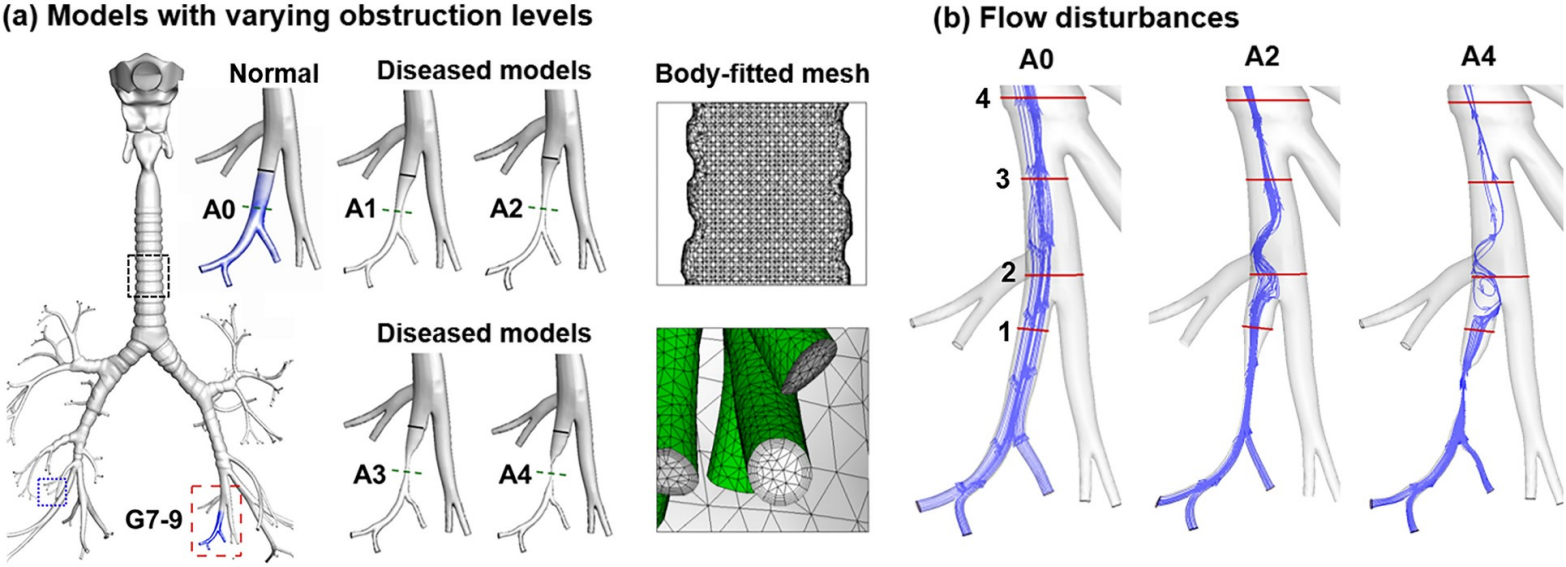
Diseased lung models and disturbed respiration. (a) One normal (A0) and four diseased bronchioles (A1-4) with increasing levels of constriction due to inflammation, excessive mucus secretion, or tumorigenesis. Body-fitted computational mesh was generated for high-fidelity physiology-based modeling and simulations. (b) The airflow was altered due to the airway constrictions.

The respiratory airway model of this study extended from the mouth opening to the ninth generation (G9) bronchioles (Fig 2a). The upper airway was originally reconstructed from CT images of a 53-year-old male that comprised the oral cavity, pharynx, and larynx [34]. The lung geometry was developed from an anatomical replica [35]. It had a functional residual capacity (FRC) of 3.5 L [36] and was consistent with the morphometric dimensions (i.e., branch diameter, length, and angle) reported in Heistracher and Hofmann [37]. A total of 115 outlets were retained in the normal lung model, which was further modified to produce four diseased models with varying severities (Fig 2a). Using HyperMorph 10.0 (Troy, MI), branches (red rectangle, Fig 2a) of G7-9 of the lower left lobe was deformed to generate airway obstructions (A1, A2, A3, A4) by progressively reducing the bronchiolar diameter. More details of the HyperMorph can found in [12, 38]. Table 1 lists the dimensions of the deformed bronchiole in terms of the diameter (mm) and cross-sectional area (mm^2^) at the constriction, as well as the volume (mm^3^) of the disease-affected region (Fig 2a).

**Table 1:** Dimension of normal and diseased airways.

	A0	A1	A2	A3	A4
Minimum bronchiole diameter (mm)	3.74	1.88	1.13	0.87	0.25
Minimum cross-sectional area (mm^2^)	11.0	2.8	1.0	0.6	0.05
Affected bronchiole volume (mm^3^)	189.9	77.6	50.5	46.6	37.9

### Aerosol image generation

Physiology-based fluid-particle computations were conducted to simulate the breath tests and generate the exhaled aerosol images. Particle tracers were inhaled as a bolus and then exhaled to a mouth filter. A stochastic scheme was used to generate the initial aerosol profiles that consisted of 30,000 particles [39]. The low Reynolds number (LRN) *k-ω* model was used to simulate the air flows [40]. This model can accurately capture flow transitions and has been widely used in respiratory flows [41].

A well-validated Lagrangian approach was used to track the particle motion and fate. User-defined functions such as the near-wall interpolation algorithm [42] were implemented. In our previous studies, this model had provided close agreement with experimental data in the upper respiratory tract for both submicrometer [43] and micrometer particles [44]. ANSYS ICEM CFD (Ansys, Inc) was used to generate the computational mesh in the airway models. A grid independence study was conducted with varying mesh densities [45]. The final mesh consisted of 4.8 million cells with a height of 0.05 mm near the wall (Fig 2a).

### Feature extraction of exhaled aerosol images

**Fig 3:**
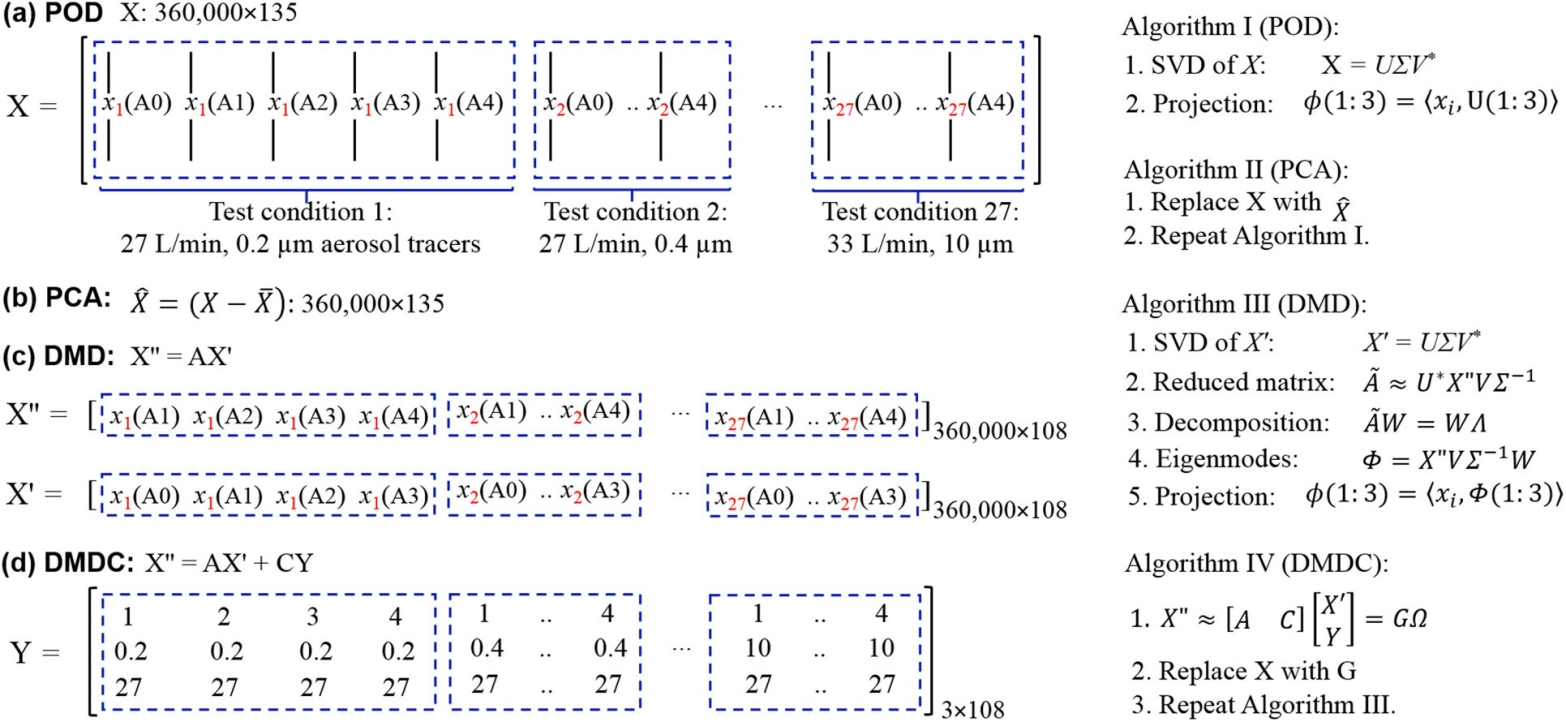
Schematic of image data processing using different SVD-based feature extraction algorithms: (a) POD, (b) PCA, (c) DMD, and (d) DMDC. Here *x*_1_(A0) represents the aerosol image as a single-column vector (360,000 = 600×600) at test condition of 27 L/min and 0.2 μm aerosol size generated in the normal lung model (A0).

In this study, we have limited time-series data (five disease stages only), which, if stacked by the disease stage, will make the data matrix extremely tall and skinny (i.e., with a high aspect ratio). Using a standard mode decomposition method, this data set can only generate five eigenmodes. This dramatic dimension reduction inevitably leads to severe information loss. To alleviate this loss, the data matrix was rearranged by dividing the data into 27 groups (three inhalation flow rates × 9 particle sizes), and in each group the five images were staked in the disease-progression sequence (i.e., following the order of A0-A4). In this arrangement, the columns of the matrix are continuous temporally within one group but have an abrupt change between different groups. These groups, even though are not correlated in a time-series manner, are correlated by inhalation rate and particle size. Therefore, both spatial and temporal correlations are needed to fully represent the data. As SVD (singular value decomposition)-based algorithms, both POD and PCA consider the spatial and temporal correlations, but in an implicit manner (Fig 3a and 3b). By contrast, DMD explicitly considers the temporal evolution of the system via the correlation matrix *A* from the discrete-time dynamical equation X'' = A*X', where X'' = [A1, A2, A3, A4] and X' = [A0, A1, A2, A3], as shown in Fig 3c. The differences between POD and PCA is that POD deals with the original image matrix X, while PCA deals with the modified image matrix $\hat{X}=\left(X-\bar{X}\right)$, where the averaged image was subtracted from each image. In the DMD algorithm, there are three additional steps than POD, which extract the time-evolution from X' to X'', as shown in Fig 3c. Compared to DMD, the DMDC algorithm can also consider the control parameters Y that elicit the observables: X'' = AX' + CY. Here the control parameters include particle size, flow rate, and airway constriction level. By organizing the discrete-time linear dynamical system as:

$$X^{\prime \prime }\approx \left[\begin{array}{cc} A & C \end{array}\right]\left[\begin{array}{c} X^{\prime }\\ Y \end{array}\right]=G\Omega$$ (1)

where G is the augmented operator matrix, Ω is the augmented data matrix that contains both image information and control information.

Once the eigenmodes are extracted, each image is projected to the coordinate system spanned by the eigenmodes, which will be further used for disease classification. To evaluate the effect of the retained number of eigenmodes, six tests were planned, which retained 3, 5, 10, 25, 75, and 100 eigenmodes, respectively.

### Disease classification

Two machine learning algorithms, support vector machine (SVM) [46, 47] and random forest (RF) [33], were selected to classify the normal and four diseased airway models (i.e., 5-class classification) due to their superior performances [48]. A ten-fold cross-validation approach was employed, in which the dataset was randomly divided into ten equal-sized subsets. In each run, a subset was used for testing and other nine subsets were used for training. The procedure was iterated ten times. In other words, each subset was used as the testing set once and as the training set nine times. The classification accuracy was calculated as:

$$Accuracy=1-\frac{total\;number\;of\;misclassified\;samples}{total\;number\;of\;samples}$$ (2)

To attain statistically averaged prediction accuracy, the ten-fold cross-validation test was repeated 100 times, and the average accuracy was computed for final comparison. The *R* package “*e1071*” was utilized to train and test the SVM/RF classifiers. One-way analysis of variance (ANOVA) was used to evaluate the classification variability in Minitab 17 (State College, PA).

## Results

### Physiology-based airflow and particle dynamics

**Fig 4:**
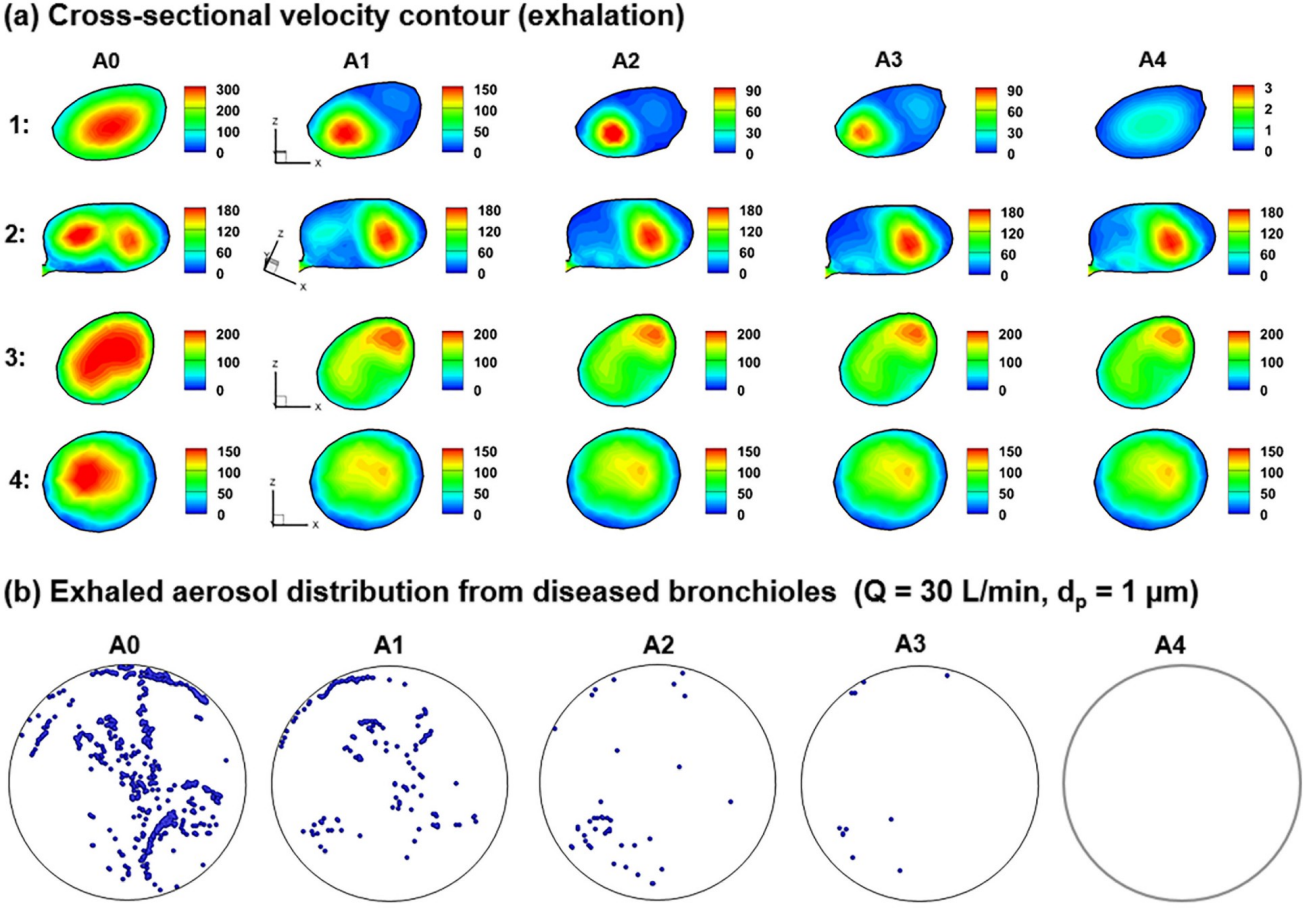
Numerically predicted airflow and aerosol dynamics during exhalation in models with varying levels of constriction: (a) cross-sectional velocity contours, and (b) distribution of exhaled aerosols that were from the diseased bronchioles only. The airway constriction disturbs the expiratory airflow. As a result, different exhaled aerosol profiles are expected due to these flow disturbances. The four locations in (a) was defined in Fig 2b.

The airway constrictions significantly alter the expiratory steam traces, as illustrated in Fig 2b. Expiration air flows in the five models are further compared in Fig 4 in terms of cross-sectional velocity contours and exhaled aerosol distributions. The presence of airway constriction causes a dramatic decrease in flow rate and perturbations in the flow field. The decreased volumetric flow rate will prevent particles from being inhaled and exhaled smoothly. The flow perturbation is evident by the variation of velocity amplitude in slice 1 and of the color depth in slice 3 of Fig 4a. In slice 2, there are two apparent peaks in A0, while one peak gradually fades away in the models of A1 to A4. The flow perturbance persists at least four bifurcations downstream of the disease site (slice 4 in Fig 3a). It is noted that particle profiles depend on both local flows and particle histories. Even though the downstream airflows may appear similar, the particle profiles can still be different because of their time-integrative characteristics.

Fig 4b shows the distributions of 1-μm aerosol tracers exhaled only from the diseased site under normal breathing conditions (30 L/min). Remarkable differences are noted among these aerosol patterns. The number of particles exhaled from the constricted bronchioles decreased gradually from A0 to A4. In the extremely constricted scenarios (A3-A4, 94.5–99.5% constriction of the cross-sectional area at the disease site, Table 1), negligible or no particles were exhaled. Interestingly, the aerosol distributions in A1 or A2 were not simply diluted versions of A0 but showed substantial variations in both pattern and intensity. This was because that a locally remodeled structure not only alters the local flow pattern but also affects the entire respiratory flow.

**Fig 5:**
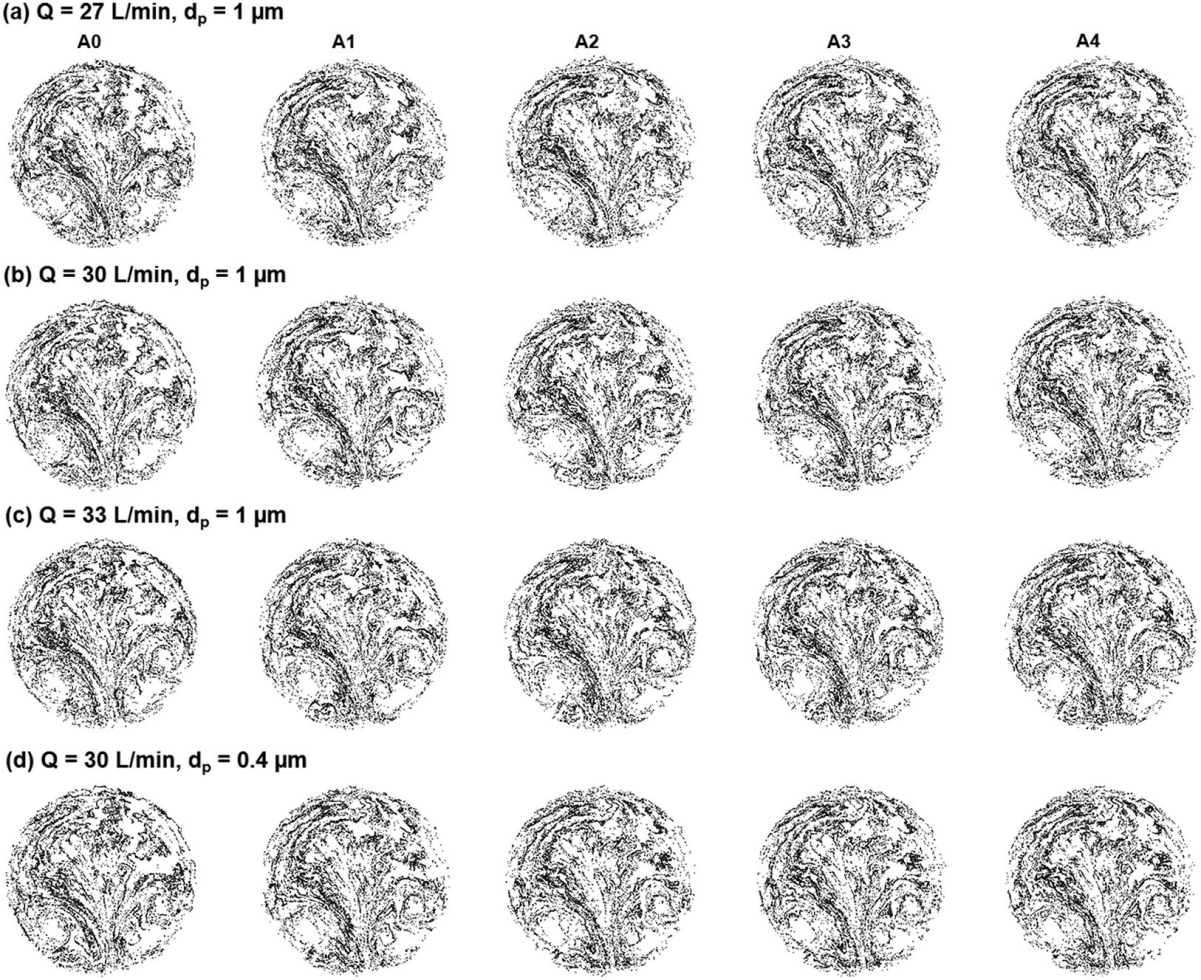
Comparison of exhaled aerosol patterns among normal (A0) and diseased (A1-A4) models for different flow rates and particle sizes: (a) *Q* = 27 L/min, *d*_*p*_ = 1 μm, (b) *Q* = 30 L/min, *d*_*p*_ = 1 μm, (c) *Q* = 33 L/min, *d*_*p*_ = 1 μm, and (d) *Q* = 30 L/min, *d*_*p*_ = 0.4 μm.

The exhaled aerosol distributions from all bronchioles are shown in Fig 5 for different test conditions. In each test case, particles collect in a unique pattern on the mouth outlet and can be seen as the “fingerprint” of the lung. A complete list of aerosol images can be viewed in S1 Animation and S1 Fig. Both similarities and disparities in the particle patterns were observed among the five models. Exhaled aerosols are very complex and irregular in pattern. Some of them are not readily distinguishable by mere inspection. Because of the close similarities, it is a significant challenge to quantitatively characterize the images and correlate these exterior images to the interior lung diseases. An automated technique is needed that can not only quickly quantify the images, but also adequately captures the key features of these images.

### Image data preprocessing

**Fig 6:**
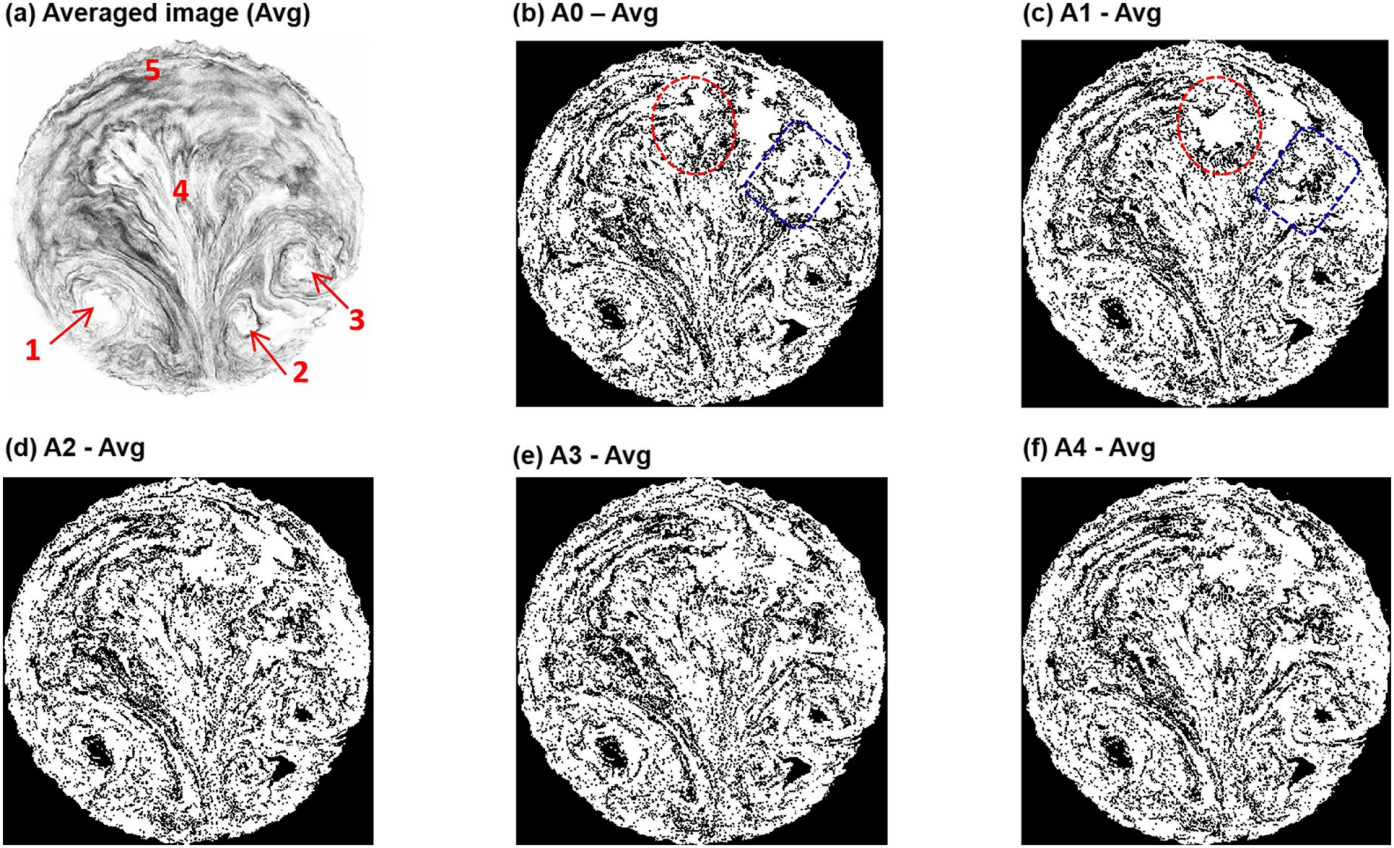
Data preprocessing: (a) averaged image of the whole database, and the images after subtracting the average for (b) A0, (c) A1, (d) A2, (e) A3, and (f) A4. The particle size for (b)–(f) is 1 μm and the inhalation flow rate is 30 L/min.

Data preprocessing was conducted in preparation to extract low-dimensional high-contrast features among disease stage in preparation for later data classification. The mean image is shown in Fig 6a that was averaged from the 405 images of the data set. This averaged image represents the overall (or background) features of the dataset with all details filtered out. There are several dominant background features, such as the three vortices denoted by arrows 1–3 (Fig 6a), a sprout-shaped pattern in the middle (label 4) and a hairpin-shaped aerosol streak on the top (label 5, Fig 6a). Such features are supposed to associate with the bifurcating structure of the lungs and the converging nature of the exhaled flow.

To isolate the specific features that are associated with diseases, each image was subtracted by the averaged image in hope to increase the contrast among images (Fig 5b–5f). For instance, apparent differences were observed between the first two images (Fig 5b *vs*. 5c), as marked in the dotted circles and rectangles. More subtle differences can also be spotted among images; however, more effective detection could be achieved by eigenmode extraction and machine learning, as discussed in the following sections.

### Feature extraction

#### POD and PCA

In this study, the proper orthogonal decomposition (POD) analysis was based on the singular value decomposition (SVD) of the original aerosol images (i.e., A), while the principal component analysis (PCA) was based on the image variance from the mean (i.e., A-Avg).

**Fig 7:**
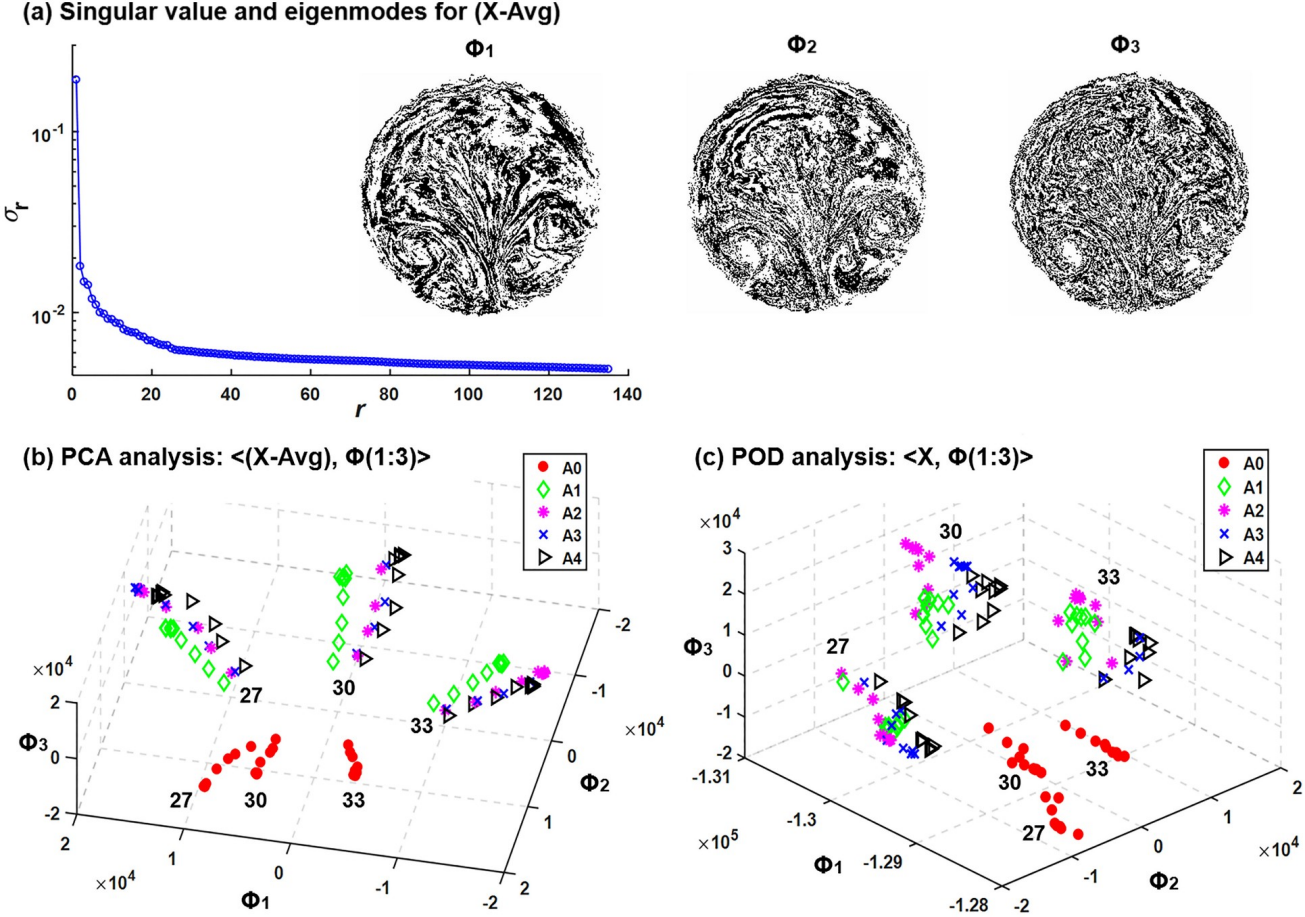
**Feature extraction and dimension reduction using principal component analysis (PCA):** (a) singular values for the high-contrast variance images from the mean (X-Avg). The first three PCA modes (*Φ*_*i*_, *i* = 1, 2,3) are also shown. Dataset projected onto the first three PCA modes are shown in (b). In comparison, values projected onto the first three POD (proper orthogonal decomposition) modes are shown in (c).

Fig 7a shows the singular values of the (A-Avg) dataset as well as the first three PCA modes. All singular values are positive and ordered in descending amplitude. As a result, the first three modes are the most dominant features of the dataset, each associated with a unique attribute/hallmark/facet /characteristic/property/aspect captured by the PCA algorithm. Even though the dataset had already been subtracted from the averaged image, dominant features still show up in the three PCA modes, such as vortices, sprouts, and hairpins (Fig 7a). Considering the complex patterns of the particle distributions, the three PCA modes were not as interpretable as other machine learning examples, such as face recognition or surveillance video processing [26], where dominant features can be readily associated with our familiar cognations. The first PCA mode consists of streaks of concentrated particles and seems like the skeleton of the exhaled aerosol images. Particle patterns become increasingly dispersed in the second and third modes (Φ_2_, Φ_3_ in Fig 7a), presumably capturing finer characteristics of the images. In addition, by viewing the images frame by frame, a relatively continuous variation of image pattern can be detected (S1 Animation), indicating a relatively smooth transition through different disease stages.

The image dataset spanned by the three PCA modes is shown in Fig 7b. Each point represents one image, which was obtained by taking the first three columns of PCA modes *Φ* and multiplying them by the image vector. The most obvious clusters were observed to related with the flow rate. The normal case (A0) was clearly separated from the diseased cases A1-4 with a large margin. Separations among A1-4 were also achieved, but with much smaller margins. One exception is between A2 and A3, where very small differences were predicted. For comparison purposes, the image dataset based on POD modes was plotted in Fig 7c. It can be seen that less distinctive clusters were predicted among the four diseased cases A1-4, even though the normal case was satisfactorily separated.

#### DMD

**Fig 8:**
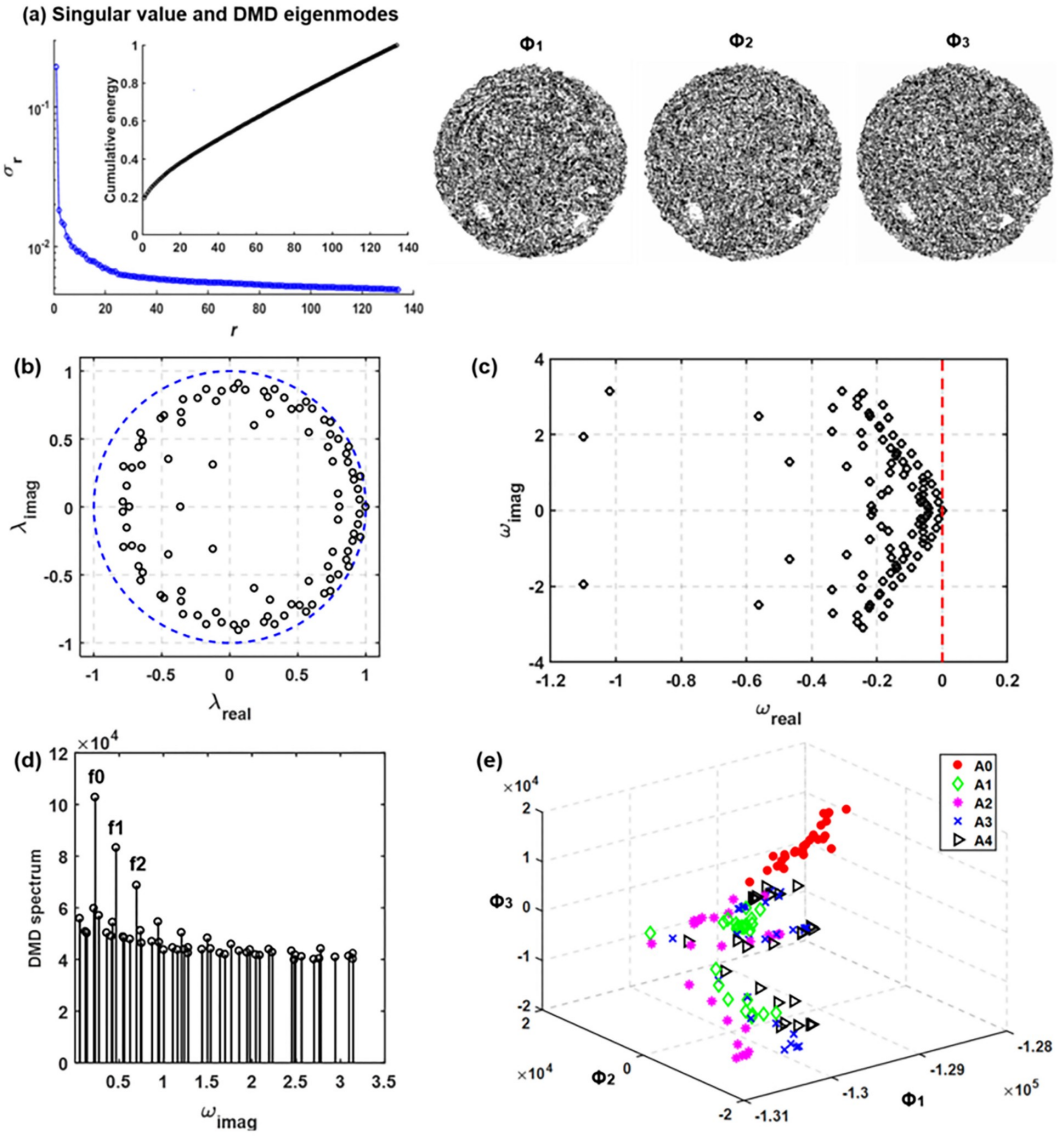
**DMD eigenvalues and energy spectrum of the exhaled aerosol images:** (a) discrete eigenvalues λ relative to the unit circle (blue dashed line) and (b) the transformed eigenvalues calculated as ω = log(λ)/(2π). The phase velocity ωi is normalized by 2π to reveal the frequency information. The DMD spectrum is shown in (d) and the projection of dataset onto the first three DMD modes are shown in (e).

Fig 8a shows the singular values, the cumulative energy, and the first three DMD eigenmodes. The singular value profile is similar to that in Fig 7a. However, the DMD eigenmodes appear very different from those of PCA (Fig 8a *vs*. Fig 7a). Contrast to the relatively well-defined aerosol skeletons in PCA modes, the DMD modes are more dispersed, which makes their differences almost indiscriminate to human eyes. In addition, the cumulative energy profile in Fig 8a shows that the first three DMD modes account for less than 30% of the bulk information of the dataset. As a result, more modes (or features) should be retained to increase classification accuracy.

Fig 8b shows the characteristics of the dynamic eigenvalue using the DMD algorithm, with each dot corresponds to one mode. As shown in Fig 8b, most of the dynamic modes fell inside the unit circle in the complex plane; only a small number of modes locating on or close to the unit circle. The distance from the origin to the dynamic mode signifies the temporal behavior of the corresponding mode, growing with time (disease progression in this study) if the distance is larger than one while decaying if smaller than one. The unit circle pattern of λ_i_ suggests that most modes are stable [6], which is reasonable for the present sequentially converging flow during exhalation, while those dynamic modes fallen on or close to the unit circle might result from the recursive usage of a selective group of flow rates and particle sizes. Eigenvalues in the interior of the unit circle are corresponding to strong damping, which leads to a stable system. Fig 8c shows the transformed eigenvalues calculated as ω = log(λ)/(2π). The read part of ω_i_ indicates the variation amplitude of the *i-th* eigenmode in response to the input (i.e., disease stages).

Three peaks of mode amplitude can be observed at frequencies f0, f1, and f2 in the DMD spectrum (Fig 8d). It is not surprising to find that f0 is associated with the variation of the inhalation flow rate (repeating every three cases), f1 with the disease stage (repeating every five cases), and f2 with the particle size (repeating every nine cases).

The image dataset spanned by the first three DMD modes is shown in Fig 8e. Surprisingly, less defined clusters were observed in comparison to those spanned by POD or PCA modes. This may result from the spatial non-orthogonality of the DMD modes may introduce a poor quality of approximation of the dataset when only a subset of modes with the largest amplitude is retained. It is noted that DMD eigenvalues are complex valued so that the dynamics have growth/decay (real part) as well as an oscillation (imaginary part). This ability to capture growth/decay of spatial patterns is important when analyzing time-varying signals and progressive behaviors. In this study, Fig 8e shows the real components only.

#### DMDC

**Fig 9:**
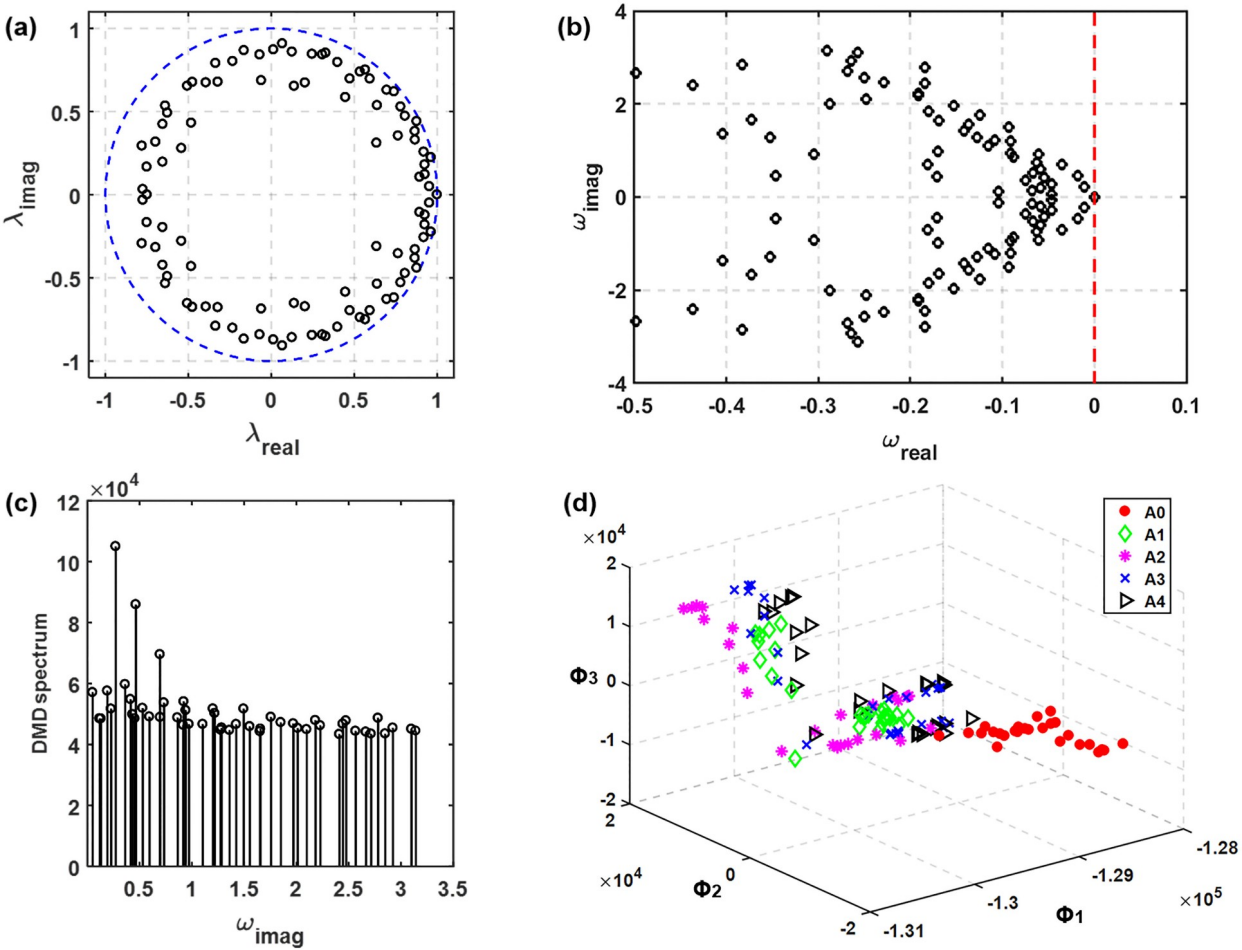
**DMDC eigenvalues and energy spectrum of the exhaled aerosol images:** (a) discrete eigenvalues λ relative to the unit circle (blue dashed line) and (b) the transformed eigenvalues calculated as ω = log(λ)/(2π). The DMDC spectrum is shown in (c) and the projection of dataset onto the first three DMDC modes are shown in (d).

Fig 9 shows the DMDC eigenvalues and energy spectrum of the exhaled aerosol images. Adding the control variables (i.e., particle size, flow rate, and airway constriction level) that caused the data discontinuity between different test conditions, the distribution of eigenvalues became more uniform than that of the DMD method. The highly damped eigenvalues in Fig 8b were eliminated in Fig 9a. This can also be noted in the reduced range of the real component of ω, i.e., from (-1.2, 0) in Fig 8c to (-0.5, 0) in Fig 9b. The DMDC was found to give rise to a very similar energy spectrum as the DMD (Fig 9c *vs*. 8d), with three dominant frequencies that are associated with inhalation flow rates, airway constriction levels, and particle sizes, respectively. With only three eigenmodes, the dataset projections do not show apparent clustering expect for the A0 class (Fig 9d), which is similar to the observation in Fig 8e. Considerable overlapping was found among data of A1, A2, A3, and A4. However, it is noted that the accuracy of classification can also be affected by the remaining less dominant features, especially when the first three features do not sufficiently capture the bulk information of the dataset, or when the classification is sought according to not-so-obvious differences.

### SVM- and RF-based classification

**Fig 10:**
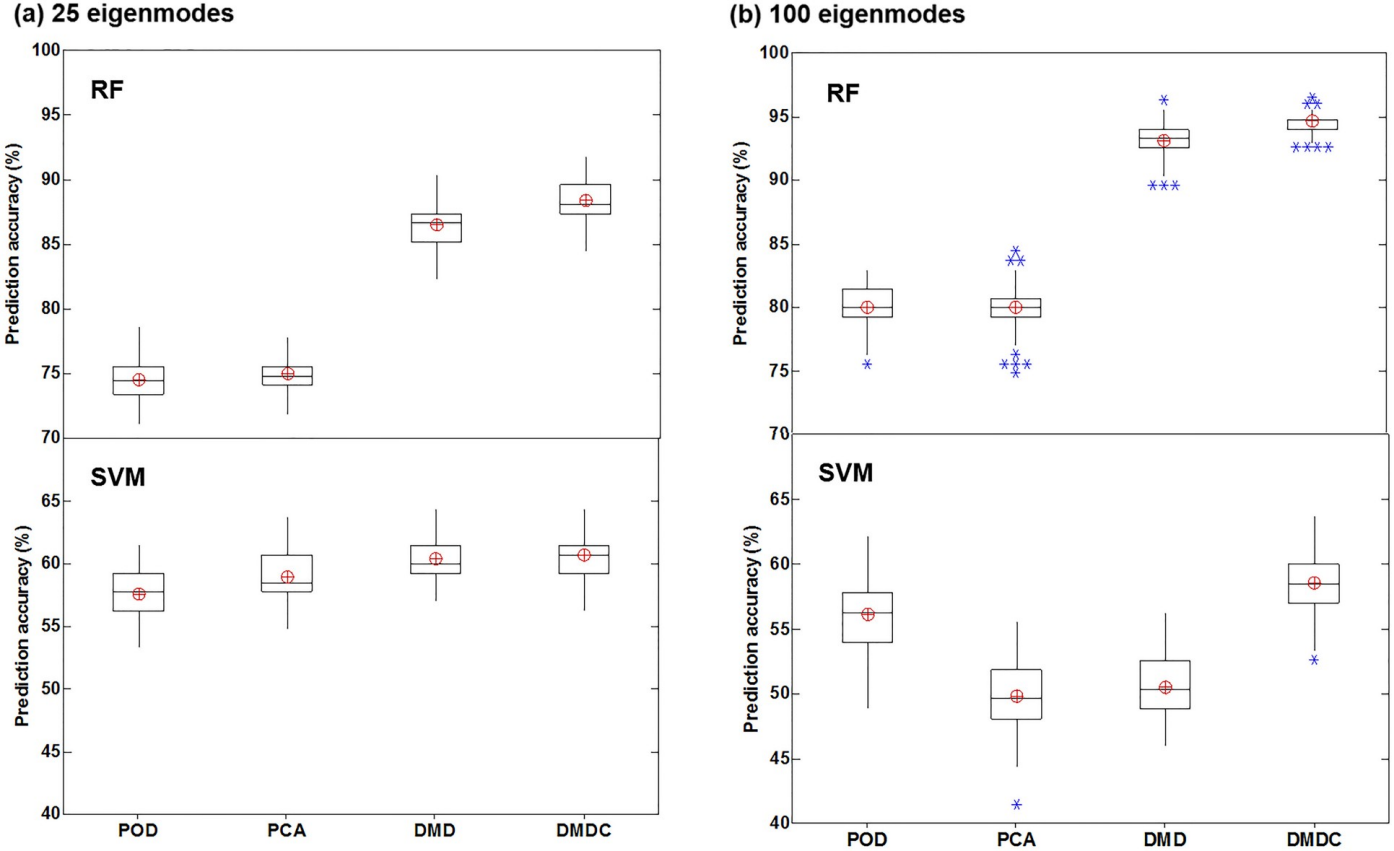
Box plot of the five-class (A0-4) classification accuracy using different combinations of feature extraction and classification algorithms with a different number of retained features: (a) 25 eigenmodes, and (b) 100 eigenmodes.

Fig 10 shows the box plot of five-class (A0-4) classification accuracy using different combinations of feature extraction and classification algorithms. These plots were obtained statistically from the classification results using ten-fold cross-validation that was repeated 100 times. To study the effect of feature vector effects, two cases were compared, with one retaining 25 eigenmodes and the other retaining 100 eigenmodes (Fig 10a *vs*. 10b). Several interesting results are noted when viewed from different perspectives. First, RF notably outperformed SVM for all feature-extraction algorithms considered here. Second, the DMD- and DMDC-based feature extraction performed much better than the POD- and PCA-based feature extraction when using the RF classification algorithm (Fig 10a and 10b, upper panel). With SVM, however, no significant difference in classification accuracy was observed among the four feature-extraction algorithms (Fig 10a and 10b, lower panel). Thirdly, retaining more features increased the variance (or uncertainty) in the classification results, as evident by the presence of outliers in Fig 10b in contrast to the absence of outlier in Fig 10a. This might have resulted from the inclusion of noise or correlated features that contaminated the disease-associated signals.

**Fig 11:**
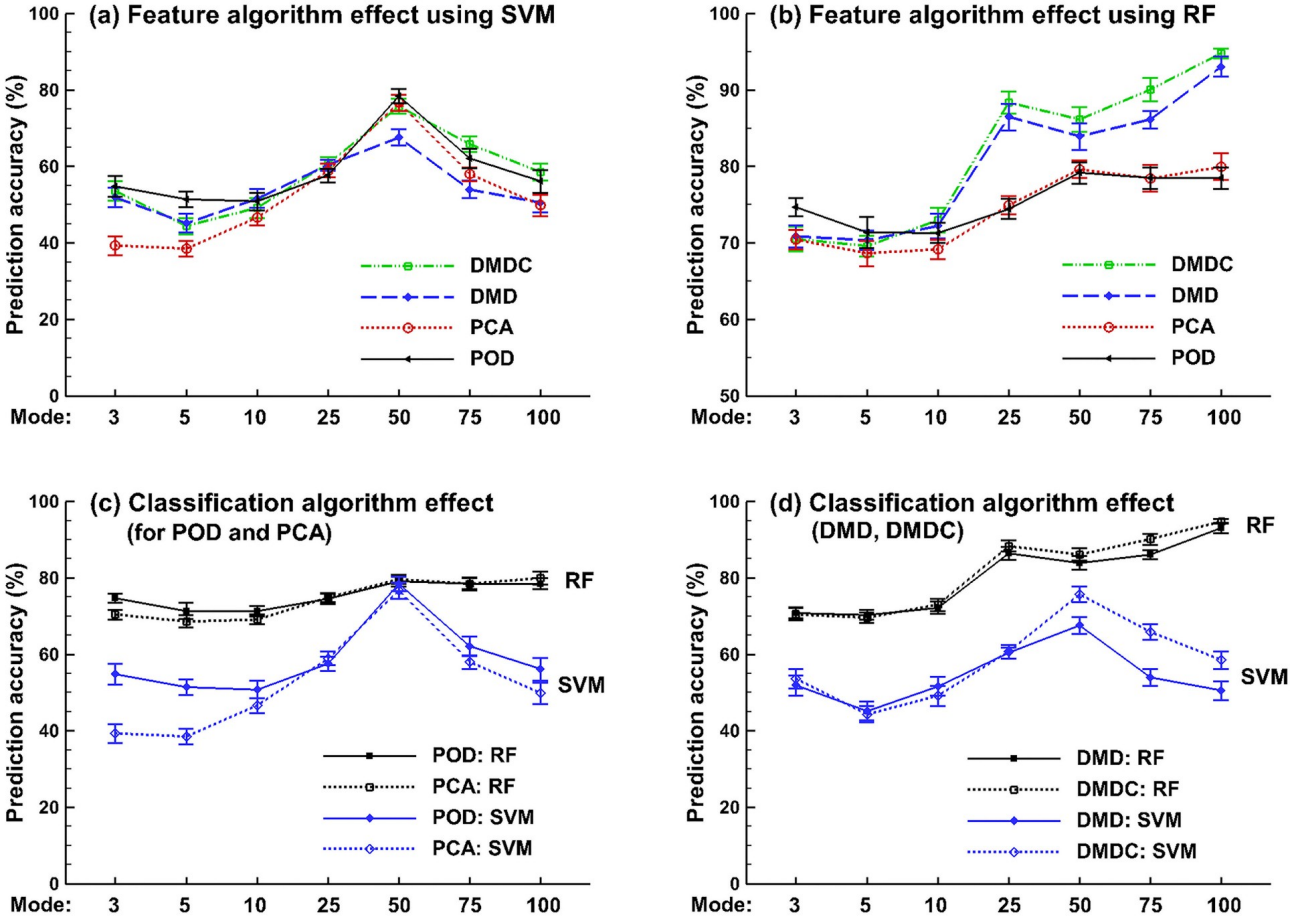
Prediction accuracy of the supervised learning algorithms for the datasets of different sampling resolutions. (a) and (b) show the box plot of the five-class classification accuracy with RF and SVM, respectively. (c) compares the performances between RF and SVM in the five-class classification. (d) compares the performances between RF and SVM in the two-class (binary) classification.

In many situations, it is not trivial to identify a subset of modes that have the strongest impact on flow and particle dynamics. The variation of prediction accuracy with the number of retained eigenmodes is shown in Fig 11. Considering that both feature extraction and classification method determined the prediction accuracy, their effects were compared separately, in Fig 11a and 11b for the feature extraction effects, and in Fig 11c and 11d for the classification algorithm effects. From Fig 11a, with SVM the best prediction accuracy was found at 50 features; increasing the number of features beyond 50 decreased the prediction accuracy. In contrast, the performance of RF either becomes asymptotic or continues to increase with more retained features (Fig 11b). With RF, the DMD and DMDC algorithms (i.e., dynamic-based) performs significantly better POD and PCA (i.e., static) when the number of retained eigenmodes is more than 10 (Fig 11b). Moreover, the DMDC performs slightly, but persistently, better than DMD (Fig 11b). Considering the classification algorithm effects (Fig 11c *vs*. 11d)., RF gave rise to constantly higher accuracy than SVM in this study, irrespective of the feature algorithms. In contrast to a relatively slow and constant increase of accuracy with the number of retained eigenmodes in RF, the accuracy in SVM varies more dramatically, which increases quickly from 3 to 50 in rank and drops precipitously thereafter (Fig 11c *vs*. 11d).

**Fig 12:**
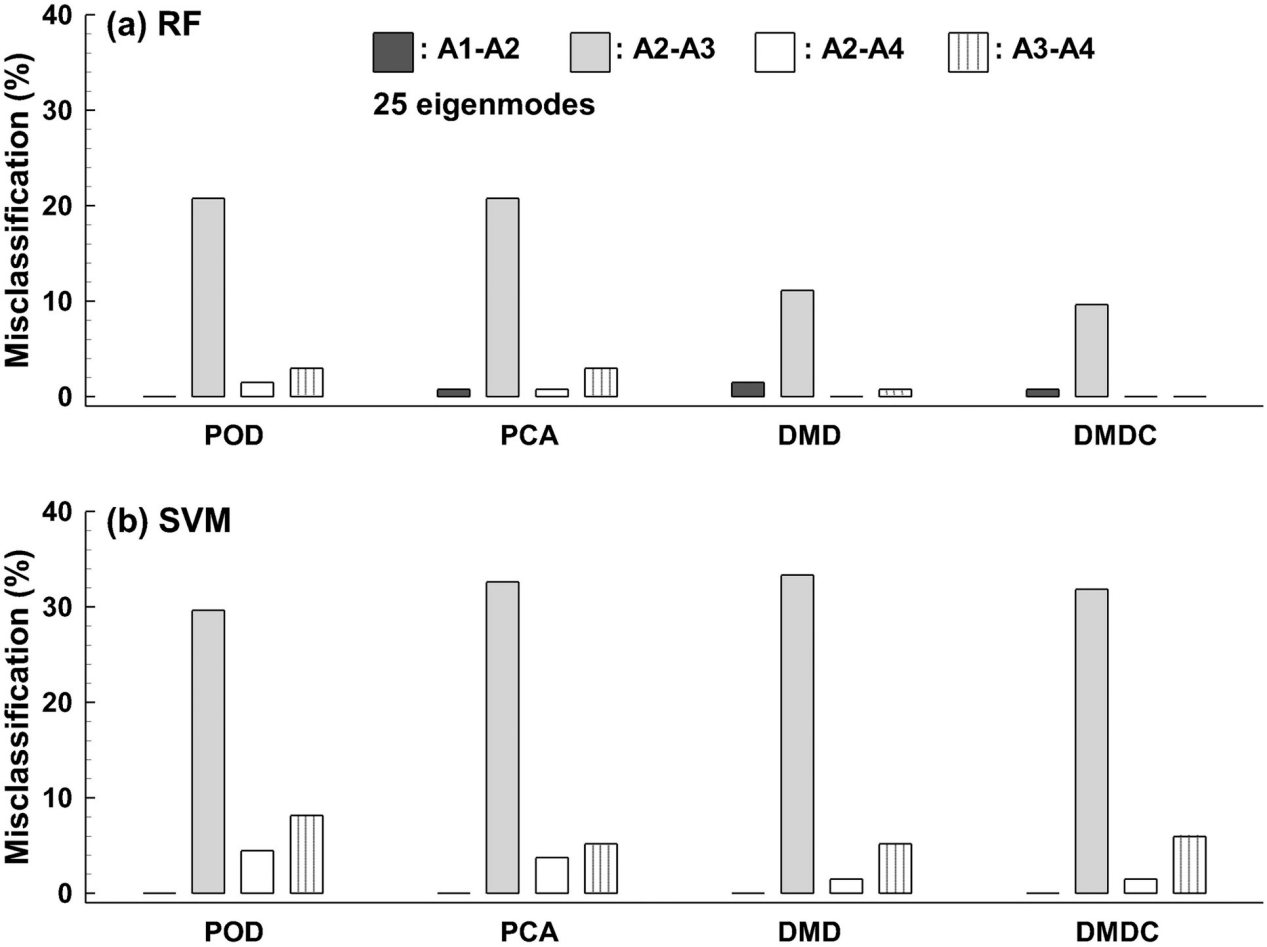
Comparison of the misclassification rate of the five-class classification among the four feature extraction algorithms using 25 eigenmodes: (a) RF and (b) SVM. The misclassification rate is zero for A0 *vs*. [A1, A2, A3, A4] and A1 *vs*. [A3, A4], which is not presented.

The misclassification rates are compared among the four feature-extraction algorithms (i.e., POD, PCA, DMD, and DMDC) in Fig 12 with 25 features retained. No misclassification was found between A0 and [A1, A2, A3, A4] and between A1 and [A3, A4] for any test in this study. For both classification algorithms, the highest misclassification rate occurred between A2 and A3, which ranged from 12–20% using RF (Fig 11a) and around 30% using SVM. (Fig 12a). The second highest misclassification occurred between A3 and A4 for both classification algorithms considered (Fig 12). In combination with the RF classifier, DMD-based features significantly reduced misclassification for A2-A3. Furthermore, adding the control parameters to DMD completely eliminated the A3-A4 misclassification in the scenario of ten-fold cross-validation repeated 100 times. Surprisingly, with SVM negligible benefits were achieved for the DMD and DMDC over the POD and PCA algorithms, as evident in Fig 12b. It is noted that the summation of the misclassification rates in each case equals (1 –prediction accuracy) of that case. For instance, the total misclassification for the POD-RF case with 25 retained features is 25.9% (= 0.7% +20.7% + 1.5% +3.0% in Fig 12a), which is equivalent to the prediction accuracy of 74.1% (1–25.9%) as shown in Fig 10a.

## Discussion

In this study, a machine learning framework with static and dynamic feature selections to classify obstructive lung diseases was presented. The impact of feature selection algorithms on classification performances was evaluated using two classification methods (SVM and RF). Results show that a classifier with features the include transient dynamics (DMD and DMDC) significantly outperformed that with static features (POD and PCA), which is consistent with the fact that disease growth is an inherently dynamic process. Also, including control parameters that are responsible for the dynamical system changes further improved the classification, but with a much smaller magnitude. On the other hand, RF gave rise a persistently higher classification accuracy than SVM, irrespective of the features. While the RF performance constantly improved with the number of retained eigenmodes, the SVM performance peaked at 50 features and decreased when more features were included. The best 5-class classification accuracy in this study was 94.8% using the DMDC-RF model, followed by 93.0% using the DMD-RF model with 100 features.

The reasons that DMD- and DMDC extracted more informative features than POD and PCA were speculated as follows. DMD and DMDC considered the temporal features associated with the disease progression from A0 to A4 and thus should better capture the phase transformation than the static feature extraction algorithms (POD and PCA). In contrast, POD and PCA extracted orthogonal coherent structures from the correlation matrix of the image ensemble; the averaging process to form the correlation tensor would lead to information loss of the disease progression. Moreover, the resulting spatial modes might not be temporally independent of each other [49], which could also compromise the performance of these two algorithms (POD and PCA).

The newly proposed DMDC-RF algorithm outperformed the fractal-RF algorithm by 7.8%. In the previous studies [9, 12], fractal dimensions were used as features to characterize the aerosol images at varying image resolutions. The optimal accuracy of the fractal-RF algorithm for five-class classification was 87.0% with each image represented by a 144 (12×12 image resolution) feature vector [12]. Even though fractals have been demonstrated to be good at characterizing complex patterns, they will inevitably lose some information, and it is unclear how such information loss will affect the data discrimination. In this study, features were extracted directly from the images in their raw form (i.e., pixel values) via SVD (singular value decomposition)-based dimension reductions to identify the most dominant coherent structures, therefore minimized the loss of information. The second improvement resulted from dynamic mode decompositions that naturally attended to the transient dynamics inherent in the disease progression. New features could be revealed that were otherwise missed by the static feature selection algorithms such as POD and PCA. The third improvement came from the inclusion of control parameters, which put more weight on features that were important for classification and suppressed irrelevant features.

Results of this study demonstrated high sensitivity of the proposed method to airway variations. The five categories (A0-A4) were defined according to the airway constriction level in one bronchiole, with the minimum bronchiolar diameter ranging from 3.74 mm in A0 to 0.26 mm in A4 (Table 1). As expected, large geometrical variation led to few misclassifications, as observed in A0 *vs*. A1. Conversely, misclassifications were predominantly predicted in the pair of A2 and A3 (Fig 12), whose dimensions were close to each other, with a 23% difference in diameter (1.13 mm *vs*. 0.87 mm) and 8% difference in volume (50.5 mm^3^ *vs*. 46.6 mm^3^). The abnormally high misclassification rate between A2 and A3 suggested that their differences might be too trivial to be clinically meaningful and therefore, should not be considered as distinct stages of the disease. In comparison, there was a remarkable reduction of the misclassification rate between A1 and A2, which differed by 40% and 35% in bronchiolar diameter (1.88 mm *vs*. 1.13 mm) and volume (77.6 mm^3^ *vs*. 50.5 mm^3^), respectively.

Similar as in the previous studies [12], RF persistently outperformed SVM regardless of the feature selection algorithms. It was noteworthy that the RF performance constantly increased with the number of retained eigenmodes, while the SVM performance climaxed when the number of retained eigenmodes was 50. Both observations might arise from the limited amount of image samples in this study and the associated overfitting (i.e., inferring too much from undersampled observations) in SVM. In practice, SVM tended to exhibit more over-fitting as the number of attributes increases [50]. Adding irrelevant features could also worsen the performance by diluting the signal. By contrast, RF was more resistant to overfitting problem [10, 11]. As indicated by its name, RF (random forest) developed a myriad of decision trees and weighed voting from all decision trees to determine the final classification. In this study, 1,000 decision trees were specified to increase the classification rate. Ten-fold cross-validation tests with 100 repetitions were conducted to minimize possible over-fitting [51].

Detecting an anomaly in deep lungs is more difficult than in the upper respiratory tract because of intrinsically weaker signals in the small airways. These weak signals can result from two factors: small perturbations by themselvesand signal attenuation during exhalation. It is crucial to know whether such weak signals can be detected at the mouth and then be retrieved to the source of the signals. The proposed DMDC-RF algorithm is highly sensitive to even miniature variations in aerosol distributions when 100 features were retained, as demonstrated by the 5-class classification accuracy of 94.8% of in small airways (G7-9, bronchiolar diameter less than 1.87 mm). The disease model in this study was very small in size and has only one bronchiole deformed; it would generate a much weaker perturbation signal than actual asthmas, which often has a whole sub-region of bronchioles constricted and therefore should be more amenable to be detected. It is also noted that the models A2 and A3 had a very small difference; with 25 retained features, the misclassification between A2 and A3 can be as high as 30% for SVM and 20% for RF. However, with 100 retained features, the total misclassification rate of the DMDC-RF model reduced to as low as 5.2%. This was remarkable considering that the disease-associated differences were not the predominating ones in this study, which was intermingled with more pronounced variances such as the inhalation flow rate and particle sizes. Thus, argumentation of disease signals and attenuation of unrelated ones were conducted in DMDC to emphasize the signal of interest. This is desirable considering that many extraneous factors can exert nontrivial influences on the outputs, and it is critical for the classifier to be sufficiently sensitive to pick out the factor of interest by disentangling it from other compounding factors.

The proposed breath test is envisioned to have two steps (i.e., screening and validation) and it is noted that the method proposed herein is for the second step (validation) only. In the first step for screening purposes, a population-based classifier is needed that had been trained on the database of a specific disease. The exhaled aerosol image of the individual patient will be used as a test sample to determine the probability of this patient to develop this disease. If the probability is high, follow-up breath tests are needed for validation purposes. In the second step, the aerosol images collected thenceforth will be grouped into a new database and used to train a personalized classifier to verify the initial screening result, as outlined in this study. Because of the persistence (or progression) of the disease, common features (or feature evolution) will show up in the time sequence of aerosol images. As such, this personalized database can also be used to measure disease progression or treatment efficacy of the patient. Moreover, this database can be incorporated into the population database to improve the applicability of the population-based classifier. Other than being used as an independent diagnostic tool, the proposed method can also be used as a supplemental tool to low-dose resolution CT scans to the validate the screening results. Considering that the proposed breath test is non-invasive, low-cost and easy-to-perform, it allows a higher frequency of tests than CT. Multiple tests on a regular basis can effectively rule out misdiagnosis, thereby increasing prediction accuracy and reducing false positive rates.

In terms of classifier selection, both the SVM and random forest algorithms exhibited satisfactory performances in this study. In recent years, deep learning algorithms have become the mainstream with increasing evidence of superiority over traditional machine learning algorithms in image classification [52, 53]. One particularly appealing feature in deep learning is that feature extraction and classification can be executed at the same time. The ability of convolutional neural network (CNN) model to learn rich features at multiple levels has led to a variety of successful application in medical image analysis [54, 55]. On the other hand, unique challenges present in applying CNN models. It generally requires large datasets for effective model training, while quality medical images are often limited. In this study, a database of 405 images was tested, which was adequate in SVM and random forest classifications, but appeared insufficient for a meaningful deep learning test. Evaluations of the performance of CNN models in analyzing exhaled aerosol images are needed as more image data are becoming available.

Challenges exist before the proposed method can be applied in clinic settings. One immediate challenge is the requirement of an existent population-based classier for screening purpose, as there is no record of aerosol images at the patient’s first visit. Further work is needed to develop such a classifier (e.g., a classifier for COPD) based on exhaled aerosol images from different (COPD) patients. This can be done using the PCA/POD features and RF classification algorithm, following the method described in this study. It is acknowledged that the performance of the proposed method can be reduced when training a classifier across individuals or among different diseases. The high predictive accuracy herein could partially come from the limited number of patient (one herein), controlled airway diseases, and predefined test conditions. Therefore, results in this study should not be generalized to population-based classifiers or other respiratory diseases. Some other limitations may also affect the clinical applicability of the proposed method, such as steady flows, rigid walls, *in silico* generated aerosol images, and a limited number of disease models originated from the same lung geometry. Natural breathing is characterized by tidal breathing [44] and compliant walls [56], which should be considered in future investigations. Aerosol images were acquired from physiology-based simulations, not from *in vivo* or *in vitro* tests. Even though such aerosol images did not affect the performance evaluation of the proposed algorithm, more realistic aerosol images should be used to be clinically relevant. One advantage of using numerically generated aerosol images is that a quantitative evaluation of the classifier is possible in light of the well-defined inputs and outputs as in this study. Another benefit of model-based classification is that the *in-silico* database allows more disease types and can be an ongoing process as well. Acquiring new images helps refine the classifier, improving both its prediction accuracy and statistical power.

In addition to the above-mentioned limitations, other factors may also affect the exhaled aerosol images, such as the mouth shape, breathing rate, body position, as well as intersubjective variability. While the effects from some factors can be minimized through standardization, others need further investigations. For instance, adopting a mouthpiece during the test is expected to alleviate the impact from the shape of the mouth and tongue position. likewise, standardizing the patient’s breathing pattern (slow and steady) and sitting position (upright) may reduce the complication from breathing and body position. Considering that diseases can occur anywhere in the lung, will the inclusion of aerosol images from different branches make it too complicated to be distinguished? The answer is that it is unlikely. All exhaled aerosol patterns, no matter how complex they appear, can be quantified as a linear combination of feature vectors and used as inputs to classify diseases. Considering that different diseases will generate different aerosol patterns, a database of commonly diagnosed lung diseases can be built to train and test the classifier model. The database can be extended by including samples of new or less diagnosed diseases. As more data become available, the existing classifier can be refined to increase the extent of its applicability.

In summary, eigenmode-based feature extractions were explored to characterize the exhaled aerosol images. RF was inherently a better classifier than SVM in classifying lung diseases. Features that included transient dynamics (DMD and DMDC) outperformed the conventional SVD-based features such as POD and PCA. The DMDC-RF (or DMD-RF) model was demonstrated to have a high sensitivity to lung structural remodeling [94.8% (or 93.0%) for 5-class classification] and is recommended for future machine-learning-based lung diagnosis. The inclusion of the evolving dynamics through DMD (or DMDC) can assist in machine-learning-based decision-making (i.e., diagnosis, prognosis, and treatment) of obstructive lung diseases in small airways.

## Supporting information

S1 Animation A list of the images of exhaled aerosol distributions under different airway constriction levels. (GIF)

S1 Fig Images of exhaled aerosol distributions under different airway constriction levels. (ZIP)
