# Supplementary figures and images for: Correlating exhaled aerosol images to small airway obstructive diseases: A study with dynamic mode decomposition and machine learning (part 1 of 2)
Source: PLoS One. 2019 Jan 31;14(1):e0211413. doi: 10.1371/journal.pone.0211413 (PMC6354993; doi:10.1371/journal.pone.0211413)

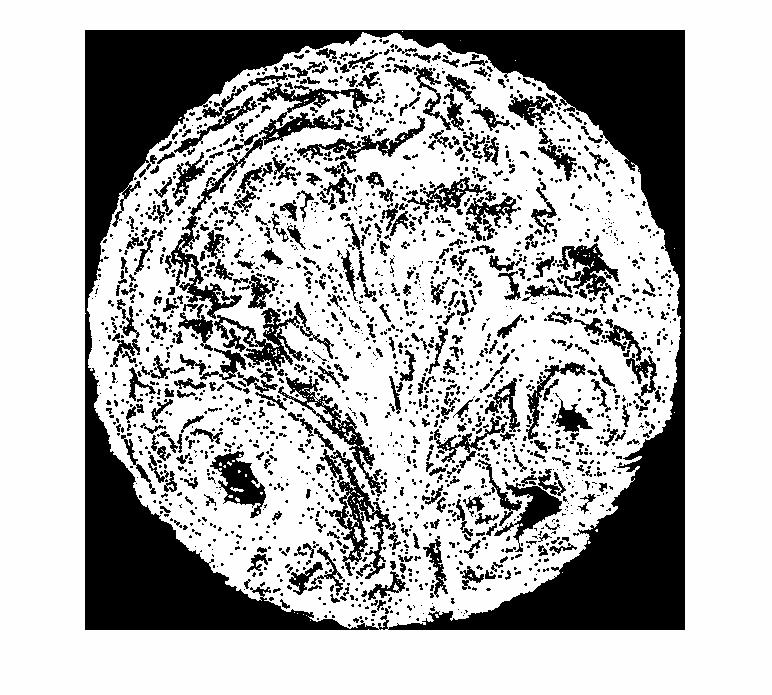

Supplement: S1 Animation — (GIF) [file pone.0211413.s001.gif]

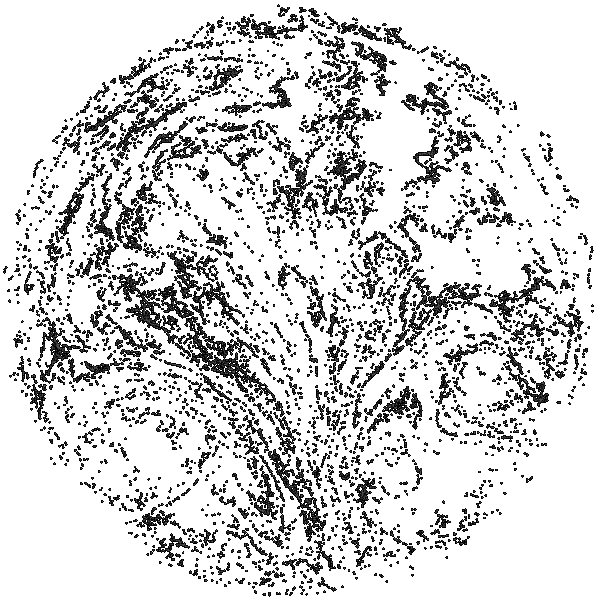

Supplement: S1 Fig — (ZIP) [file pone.0211413.s002.zip › Test images/S0_01.bmp]

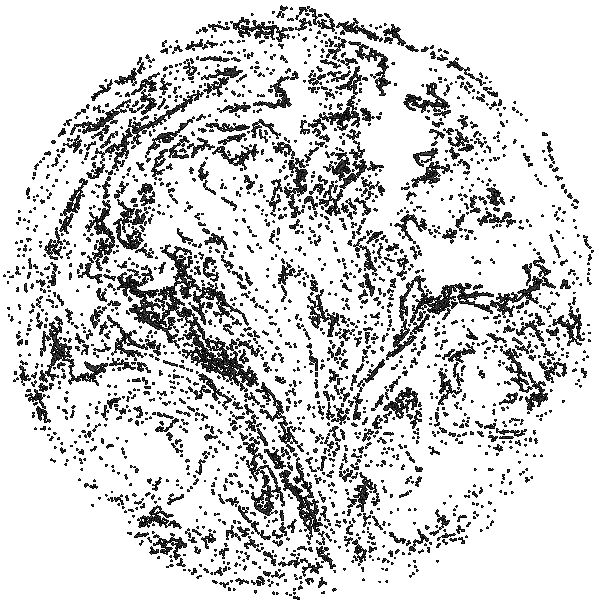

Supplement: S1 Fig — (ZIP) [file pone.0211413.s002.zip › Test images/S0_02.bmp]

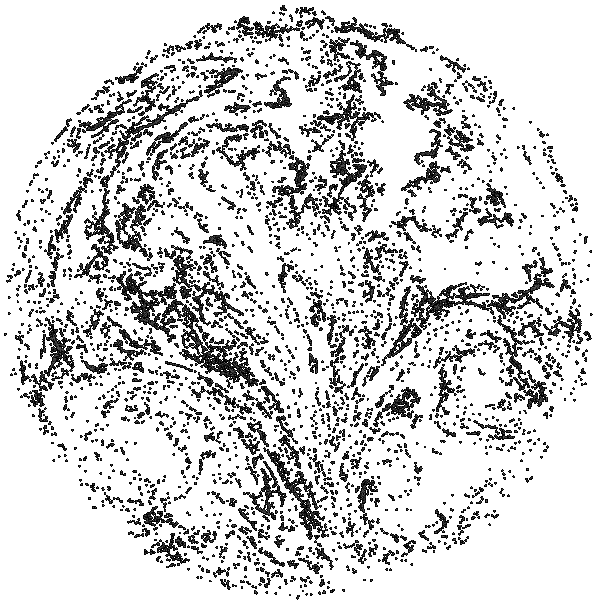

Supplement: S1 Fig — (ZIP) [file pone.0211413.s002.zip › Test images/S0_03.bmp]

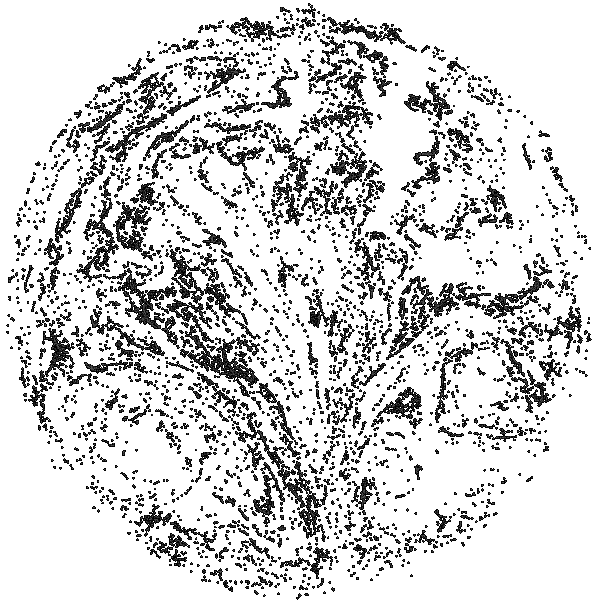

Supplement: S1 Fig — (ZIP) [file pone.0211413.s002.zip › Test images/S0_04.bmp]

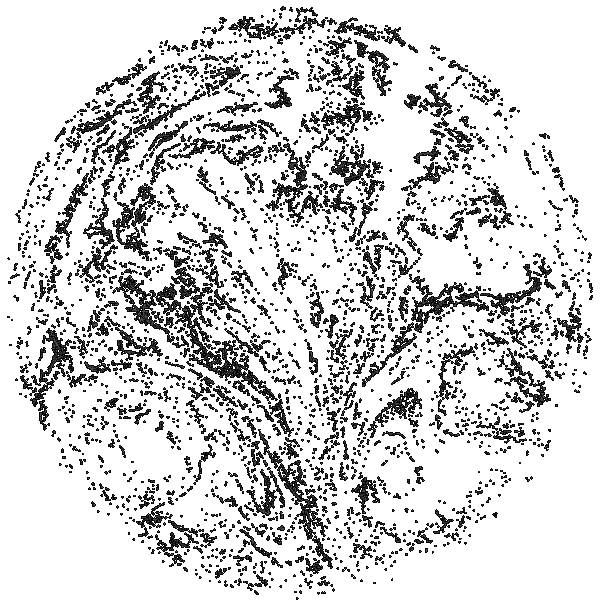

Supplement: S1 Fig — (ZIP) [file pone.0211413.s002.zip › Test images/S0_05.bmp]

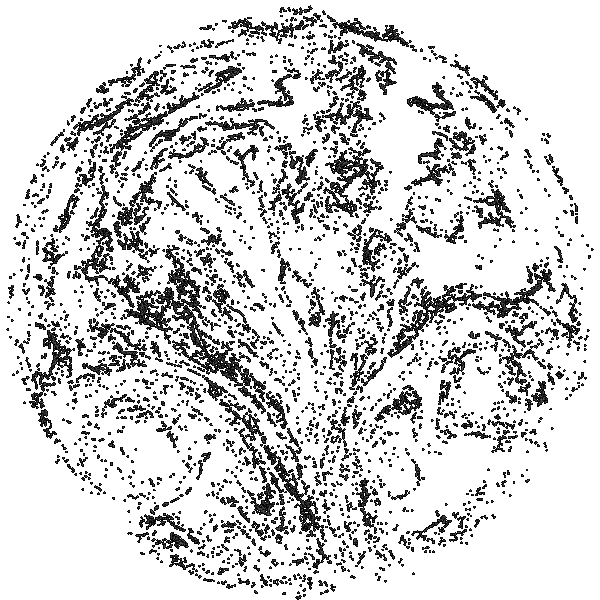

Supplement: S1 Fig — (ZIP) [file pone.0211413.s002.zip › Test images/S0_06.bmp]

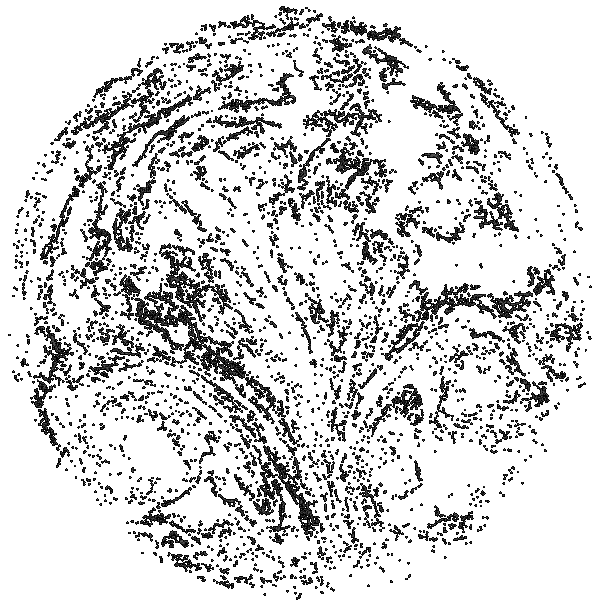

Supplement: S1 Fig — (ZIP) [file pone.0211413.s002.zip › Test images/S0_07.bmp]

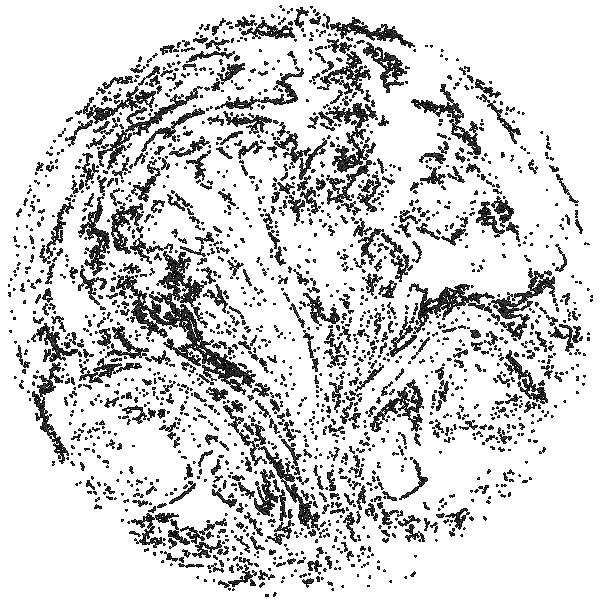

Supplement: S1 Fig — (ZIP) [file pone.0211413.s002.zip › Test images/S0_08.bmp]

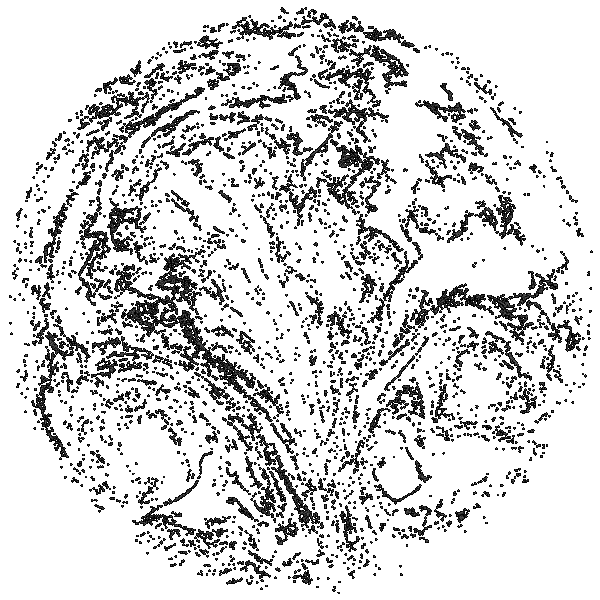

Supplement: S1 Fig — (ZIP) [file pone.0211413.s002.zip › Test images/S0_09.bmp]

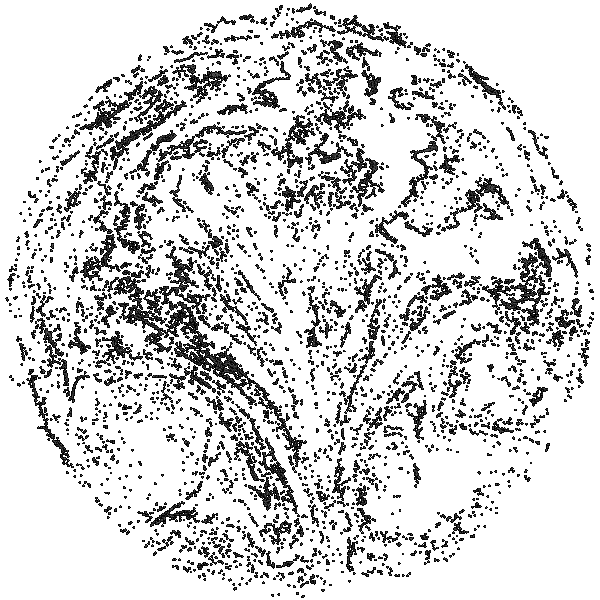

Supplement: S1 Fig — (ZIP) [file pone.0211413.s002.zip › Test images/S0_10.bmp]

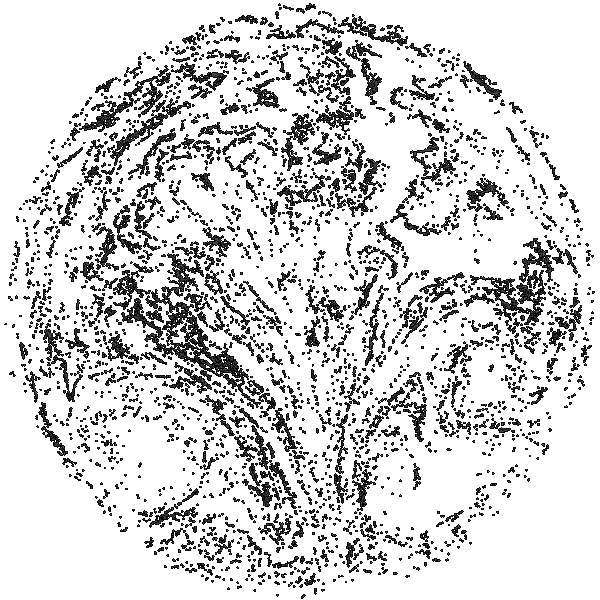

Supplement: S1 Fig — (ZIP) [file pone.0211413.s002.zip › Test images/S0_11.bmp]

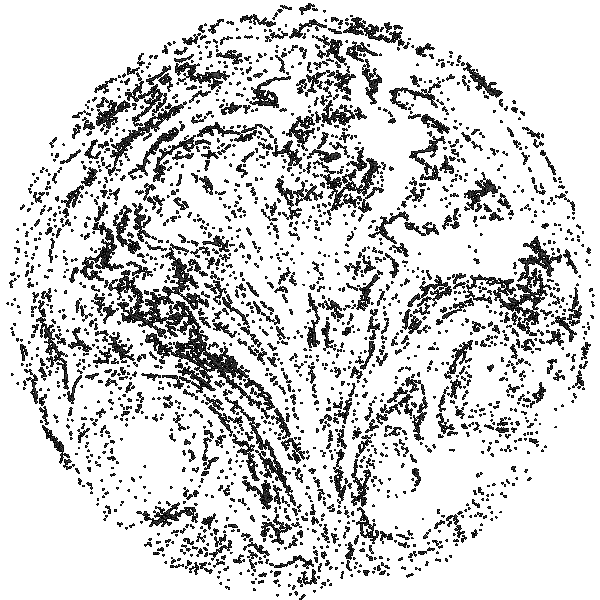

Supplement: S1 Fig — (ZIP) [file pone.0211413.s002.zip › Test images/S0_12.bmp]

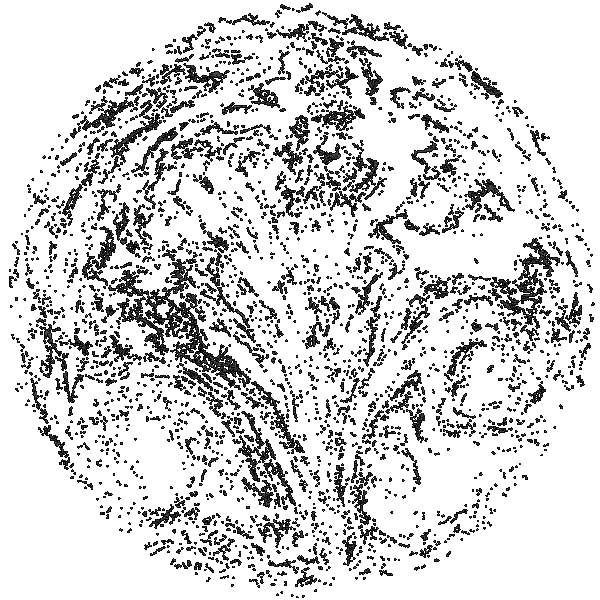

Supplement: S1 Fig — (ZIP) [file pone.0211413.s002.zip › Test images/S0_13.bmp]

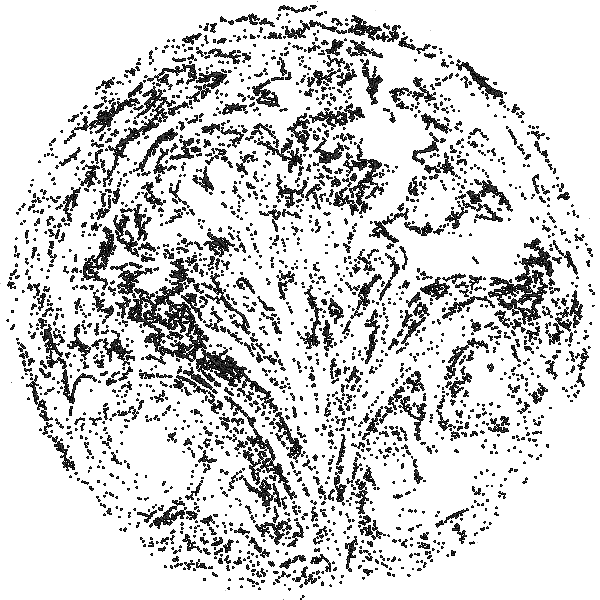

Supplement: S1 Fig — (ZIP) [file pone.0211413.s002.zip › Test images/S0_14.bmp]

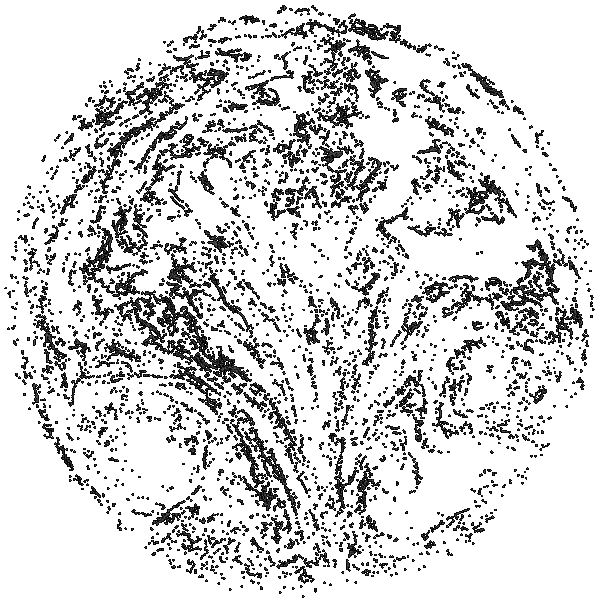

Supplement: S1 Fig — (ZIP) [file pone.0211413.s002.zip › Test images/S0_15.bmp]

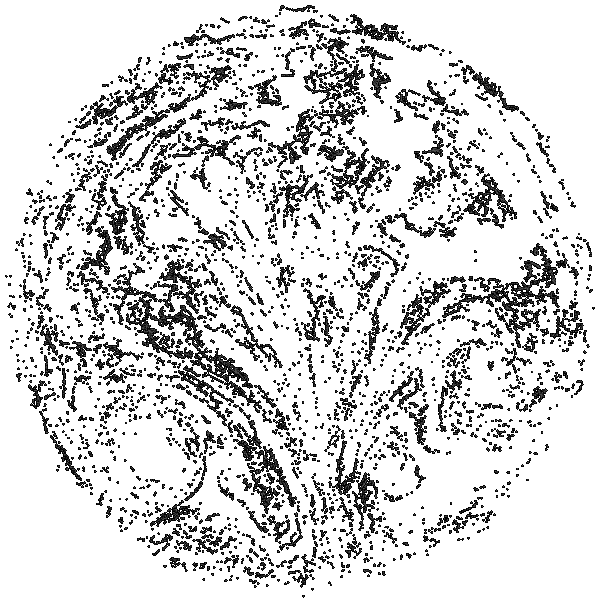

Supplement: S1 Fig — (ZIP) [file pone.0211413.s002.zip › Test images/S0_16.bmp]

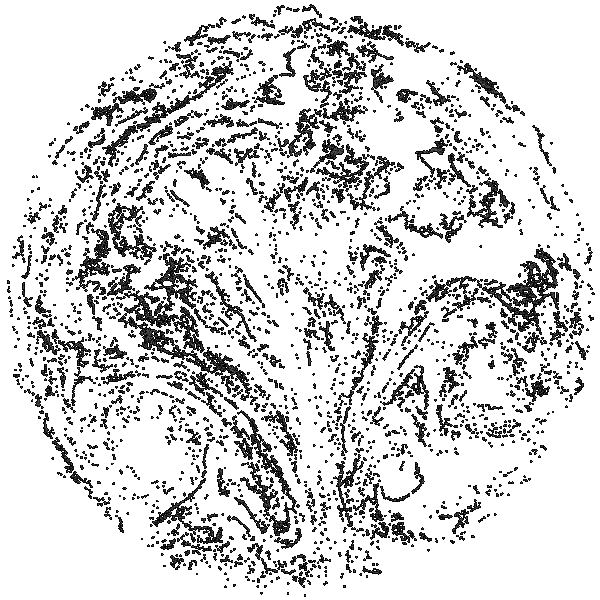

Supplement: S1 Fig — (ZIP) [file pone.0211413.s002.zip › Test images/S0_17.bmp]

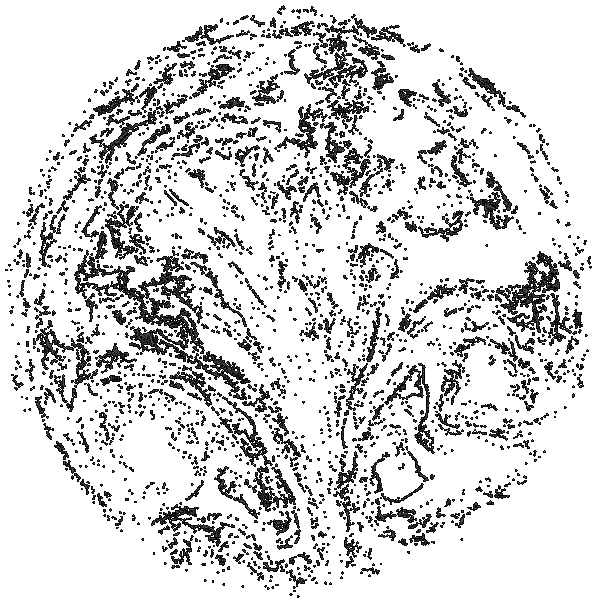

Supplement: S1 Fig — (ZIP) [file pone.0211413.s002.zip › Test images/S0_18.bmp]

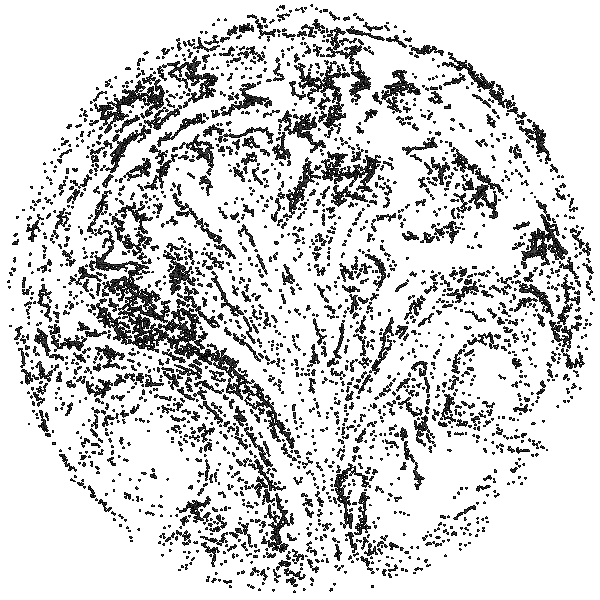

Supplement: S1 Fig — (ZIP) [file pone.0211413.s002.zip › Test images/S0_19.bmp]

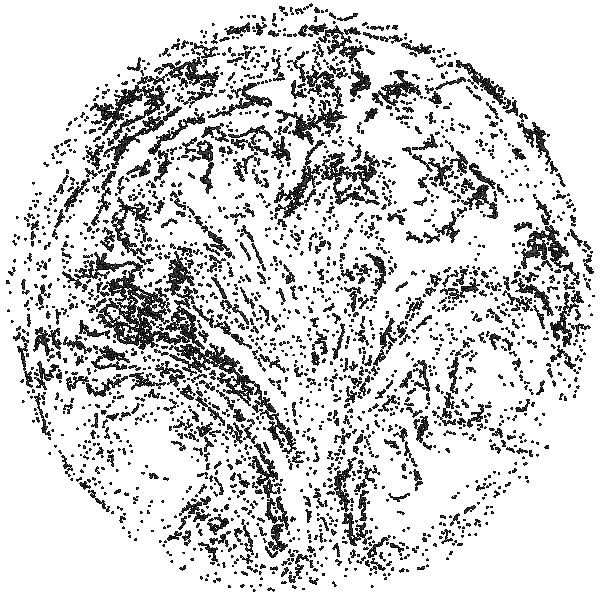

Supplement: S1 Fig — (ZIP) [file pone.0211413.s002.zip › Test images/S0_20.bmp]

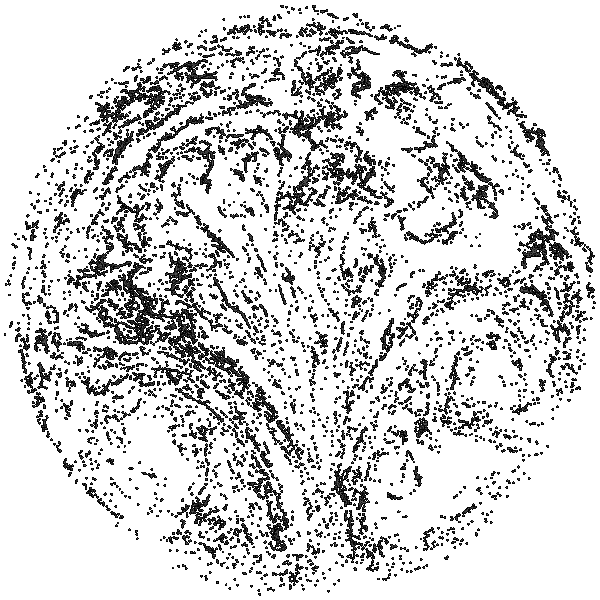

Supplement: S1 Fig — (ZIP) [file pone.0211413.s002.zip › Test images/S0_21.bmp]

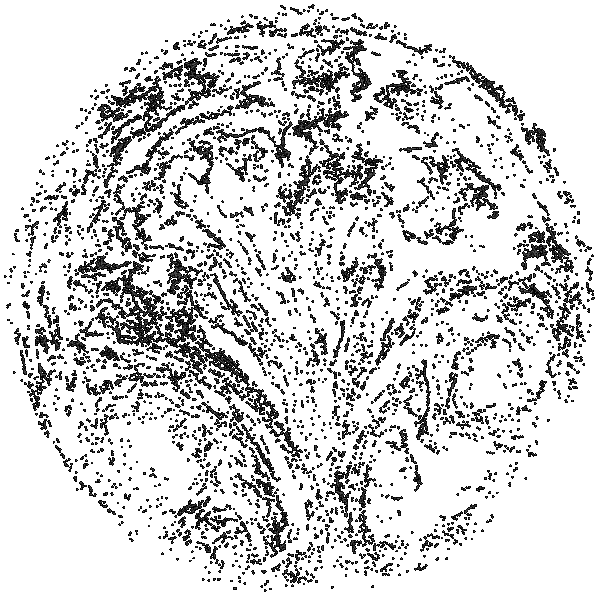

Supplement: S1 Fig — (ZIP) [file pone.0211413.s002.zip › Test images/S0_22.bmp]

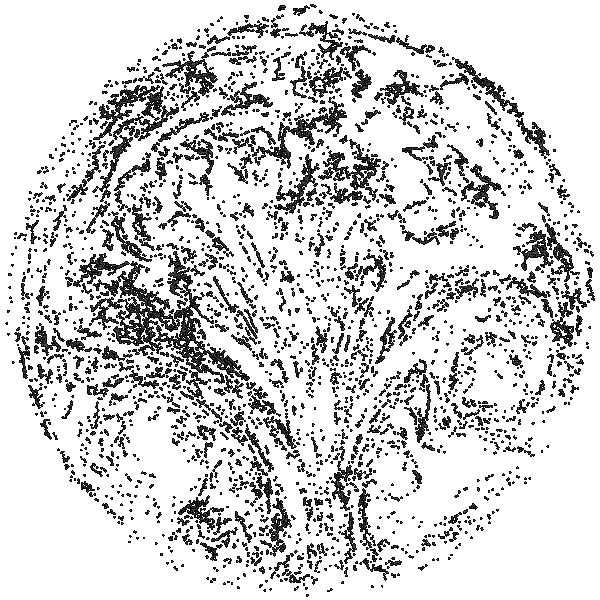

Supplement: S1 Fig — (ZIP) [file pone.0211413.s002.zip › Test images/S0_23.bmp]

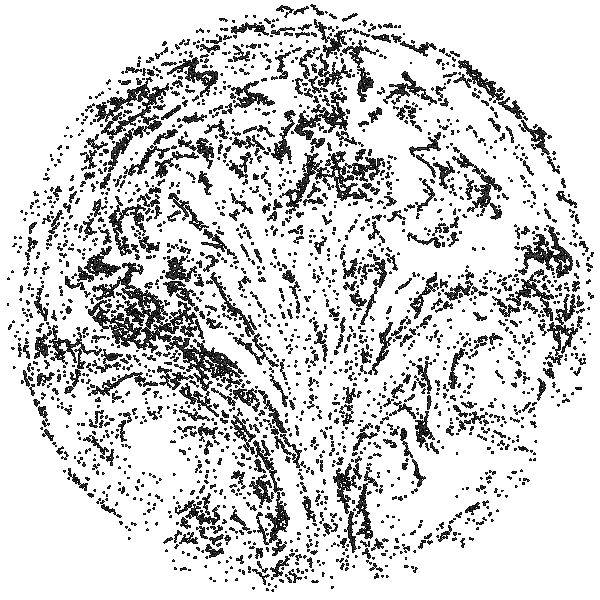

Supplement: S1 Fig — (ZIP) [file pone.0211413.s002.zip › Test images/S0_24.bmp]

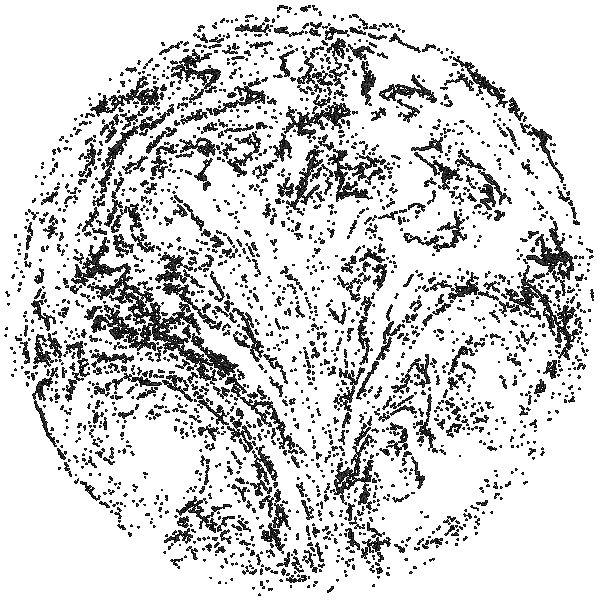

Supplement: S1 Fig — (ZIP) [file pone.0211413.s002.zip › Test images/S0_25.bmp]

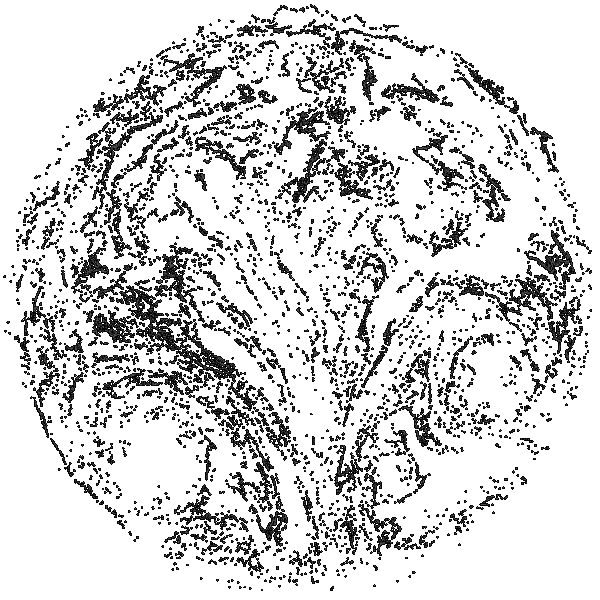

Supplement: S1 Fig — (ZIP) [file pone.0211413.s002.zip › Test images/S0_26.bmp]

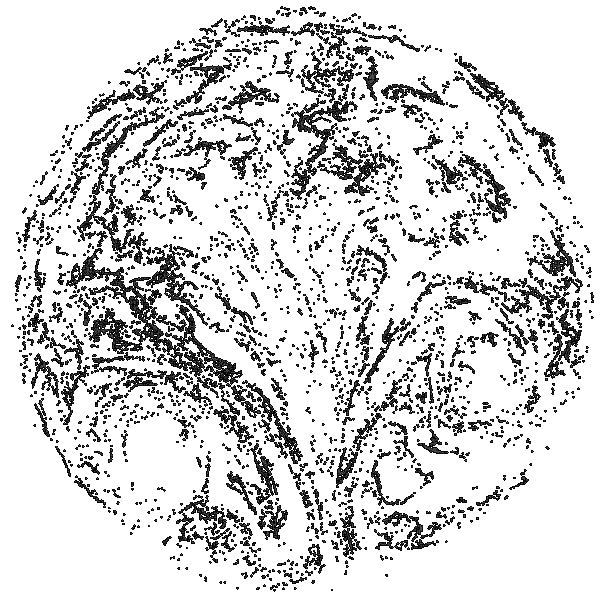

Supplement: S1 Fig — (ZIP) [file pone.0211413.s002.zip › Test images/S0_27.bmp]

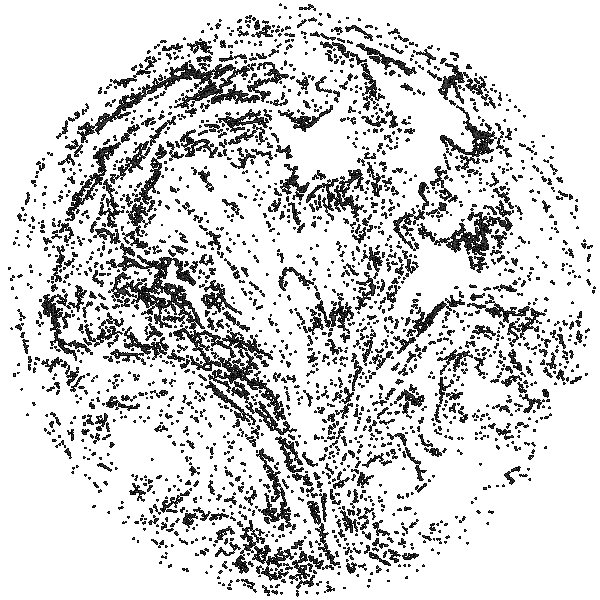

Supplement: S1 Fig — (ZIP) [file pone.0211413.s002.zip › Test images/S1_01.bmp]

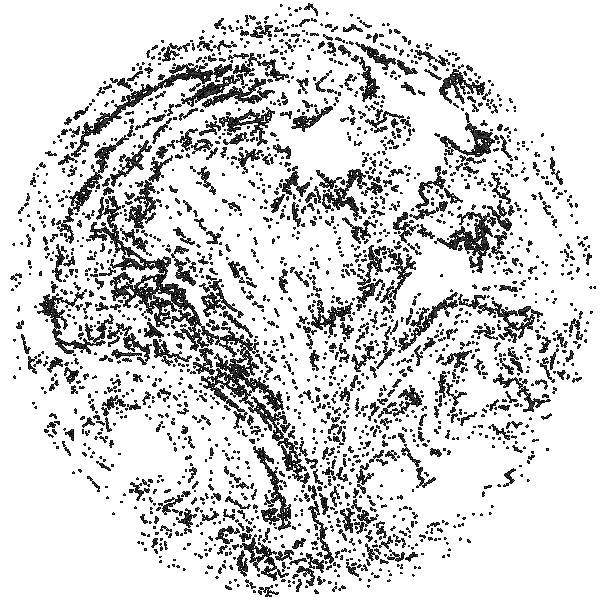

Supplement: S1 Fig — (ZIP) [file pone.0211413.s002.zip › Test images/S1_02.bmp]

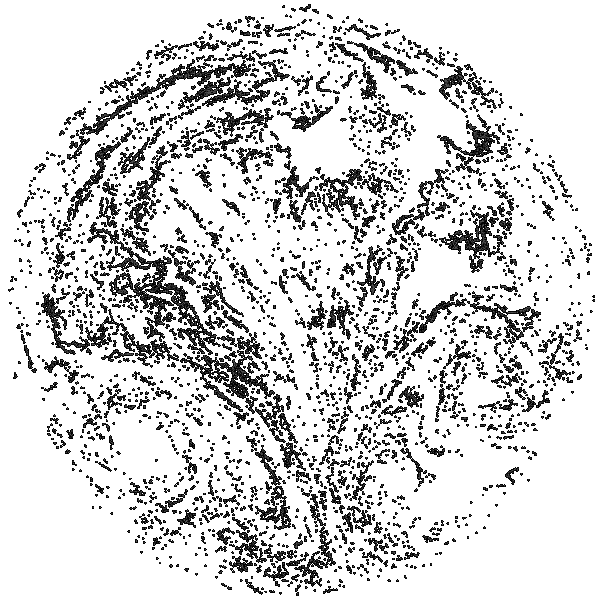

Supplement: S1 Fig — (ZIP) [file pone.0211413.s002.zip › Test images/S1_03.bmp]

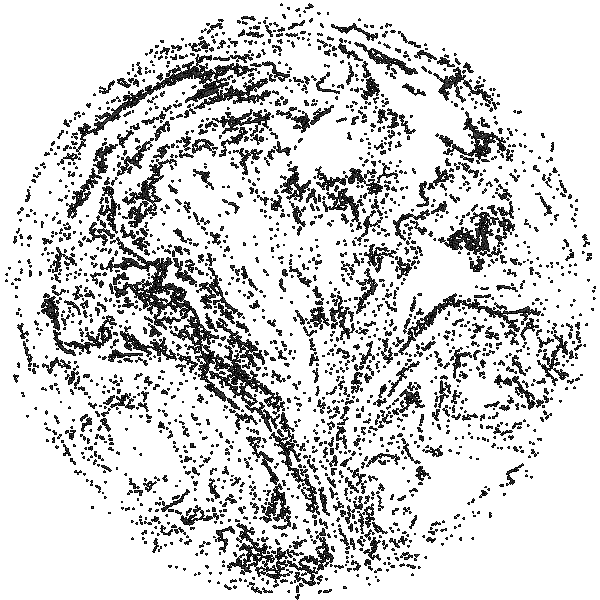

Supplement: S1 Fig — (ZIP) [file pone.0211413.s002.zip › Test images/S1_04.bmp]

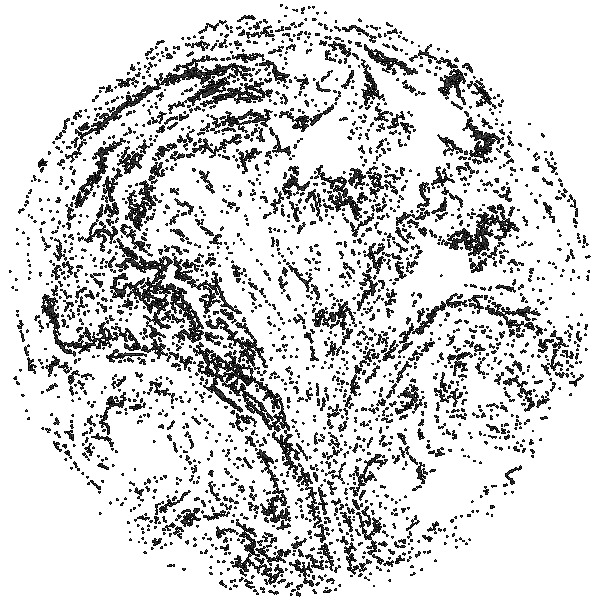

Supplement: S1 Fig — (ZIP) [file pone.0211413.s002.zip › Test images/S1_05.bmp]

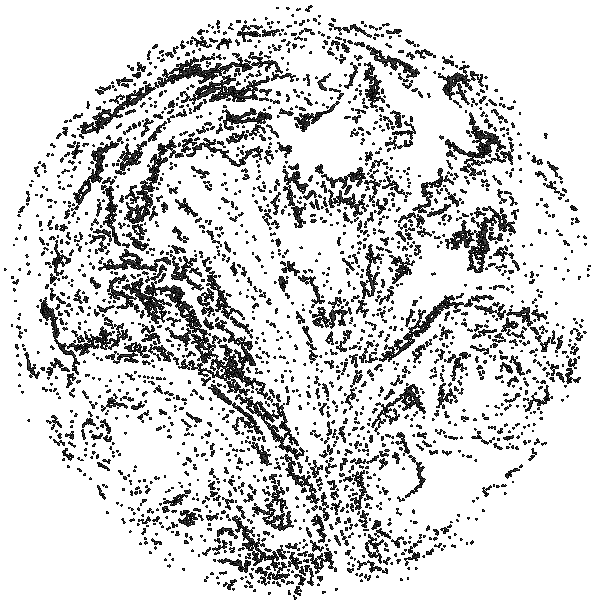

Supplement: S1 Fig — (ZIP) [file pone.0211413.s002.zip › Test images/S1_06.bmp]

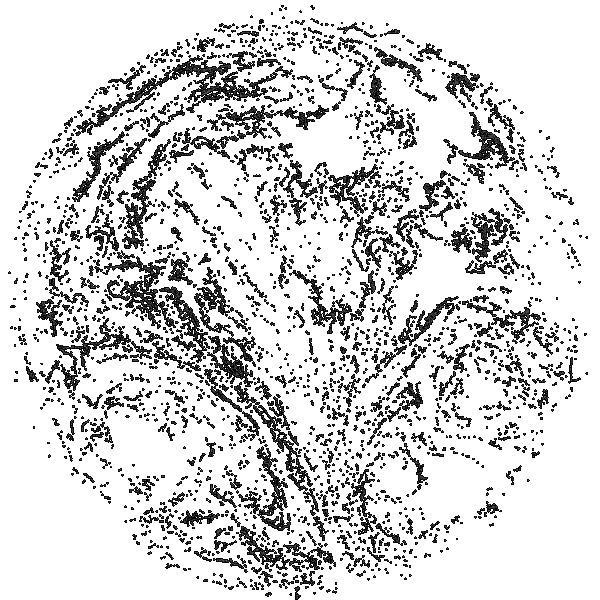

Supplement: S1 Fig — (ZIP) [file pone.0211413.s002.zip › Test images/S1_07.bmp]

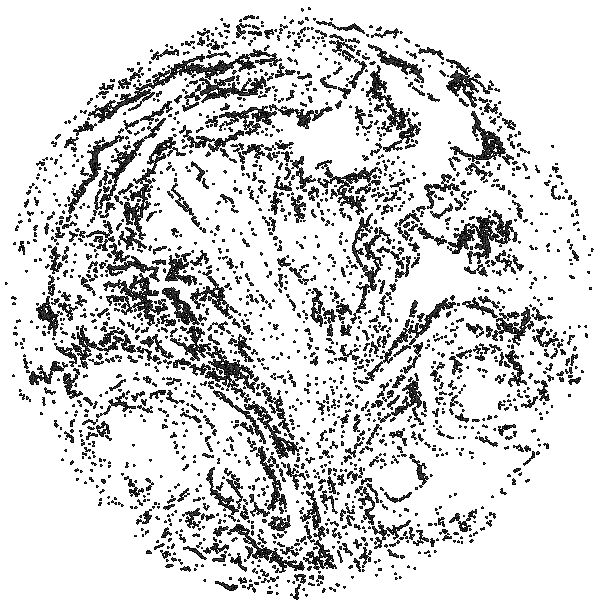

Supplement: S1 Fig — (ZIP) [file pone.0211413.s002.zip › Test images/S1_08.bmp]

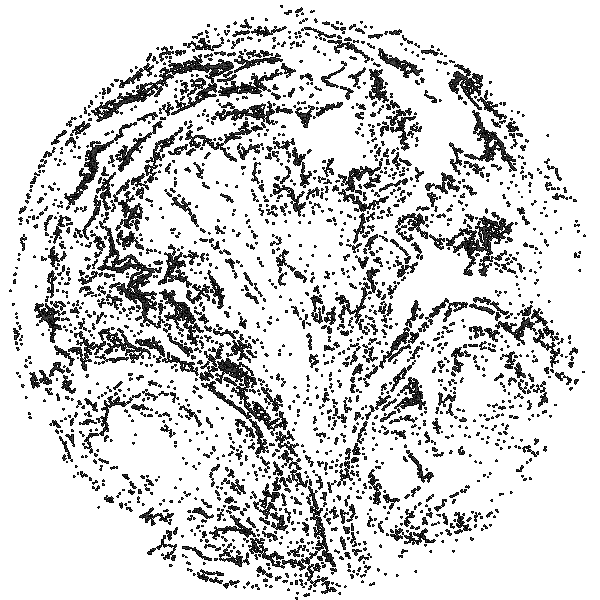

Supplement: S1 Fig — (ZIP) [file pone.0211413.s002.zip › Test images/S1_09.bmp]

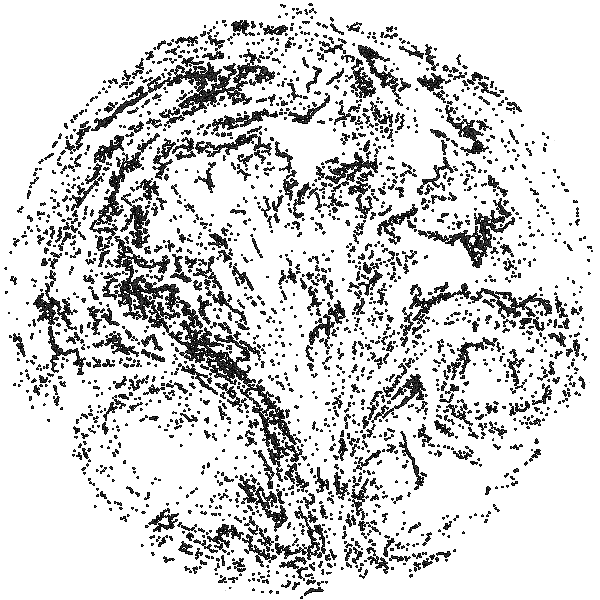

Supplement: S1 Fig — (ZIP) [file pone.0211413.s002.zip › Test images/S1_10.bmp]

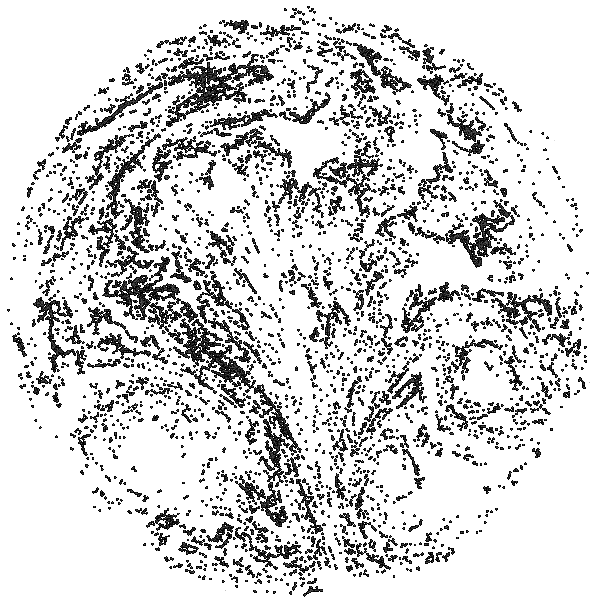

Supplement: S1 Fig — (ZIP) [file pone.0211413.s002.zip › Test images/S1_11.bmp]

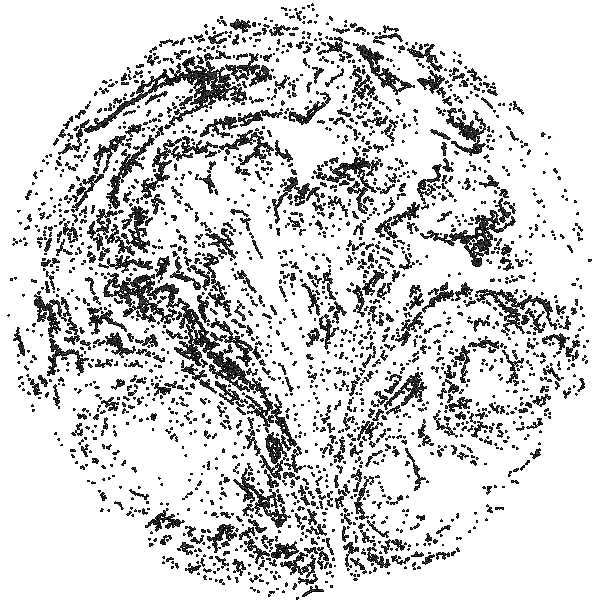

Supplement: S1 Fig — (ZIP) [file pone.0211413.s002.zip › Test images/S1_12.bmp]

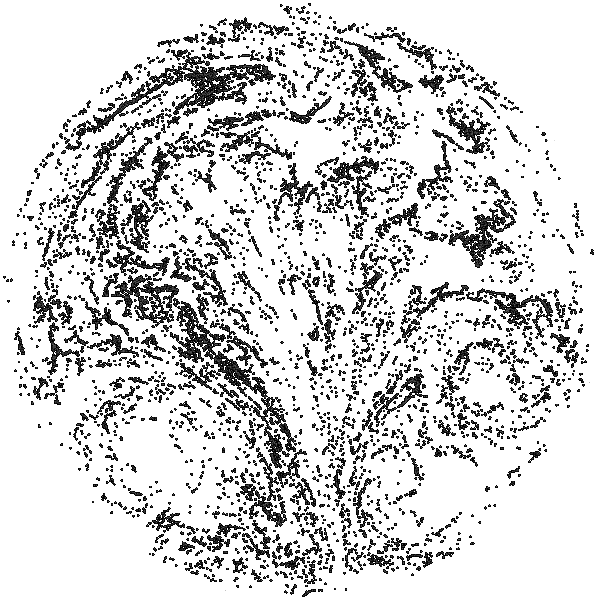

Supplement: S1 Fig — (ZIP) [file pone.0211413.s002.zip › Test images/S1_13.bmp]

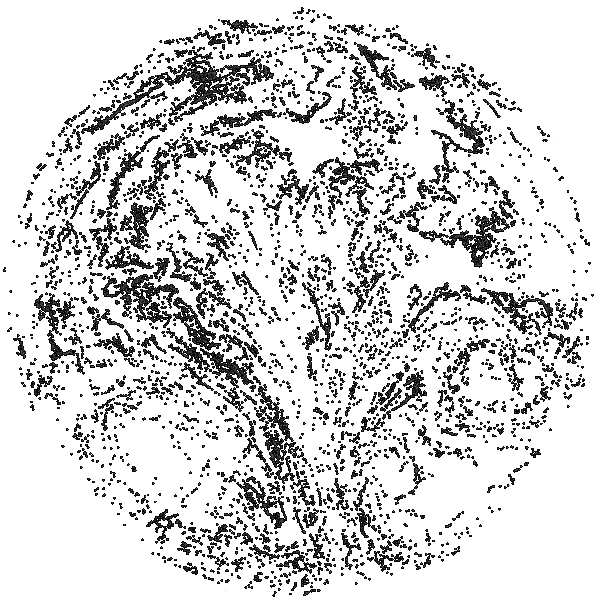

Supplement: S1 Fig — (ZIP) [file pone.0211413.s002.zip › Test images/S1_14.bmp]

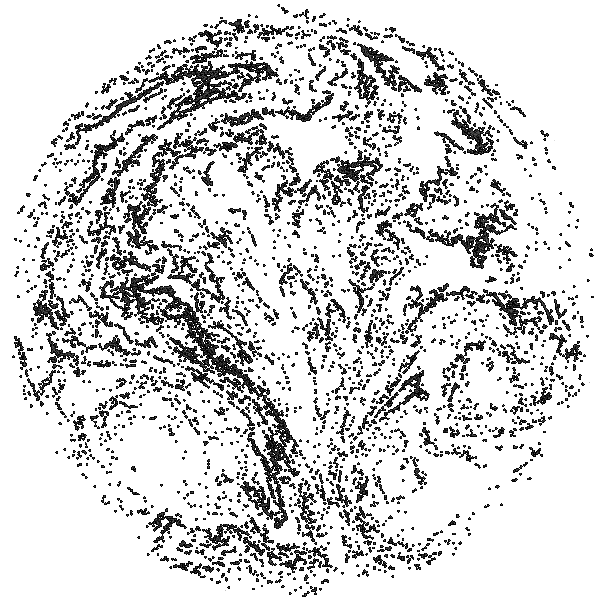

Supplement: S1 Fig — (ZIP) [file pone.0211413.s002.zip › Test images/S1_15.bmp]

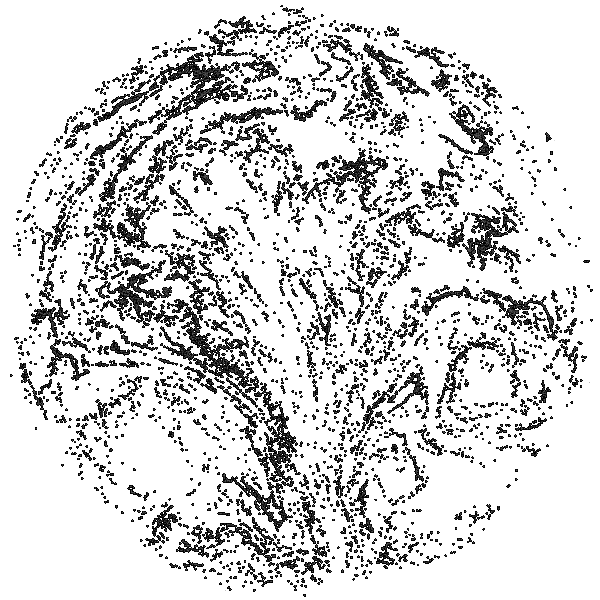

Supplement: S1 Fig — (ZIP) [file pone.0211413.s002.zip › Test images/S1_16.bmp]

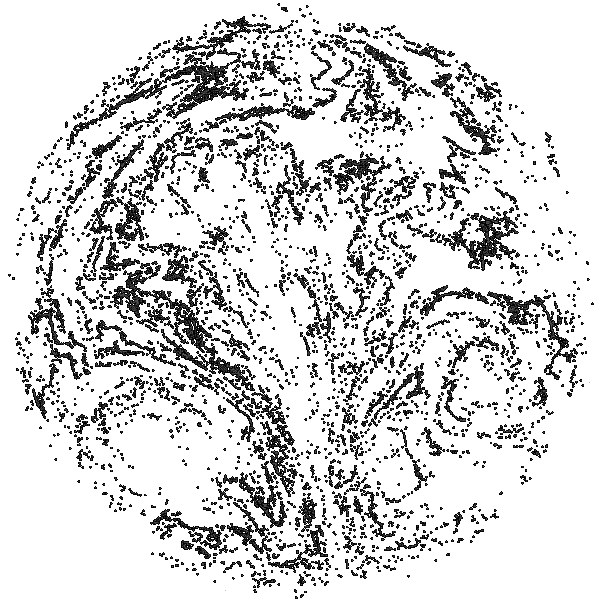

Supplement: S1 Fig — (ZIP) [file pone.0211413.s002.zip › Test images/S1_17.bmp]

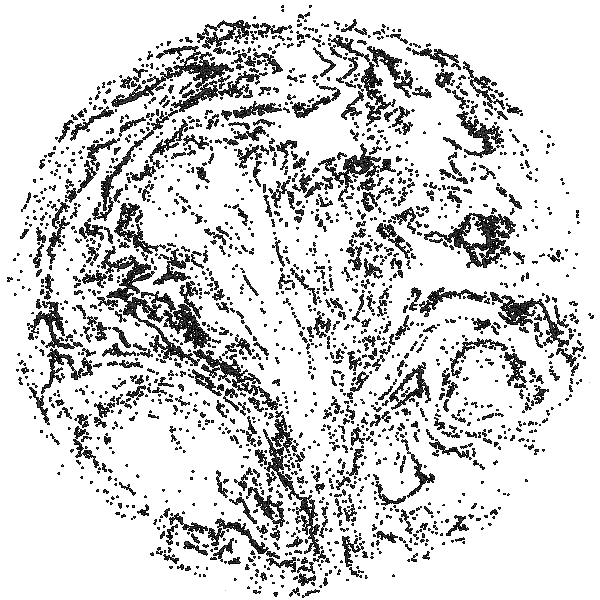

Supplement: S1 Fig — (ZIP) [file pone.0211413.s002.zip › Test images/S1_18.bmp]

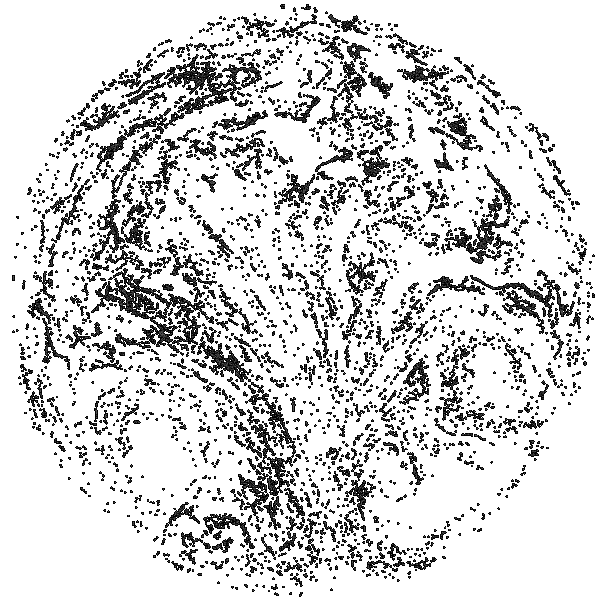

Supplement: S1 Fig — (ZIP) [file pone.0211413.s002.zip › Test images/S1_19.bmp]

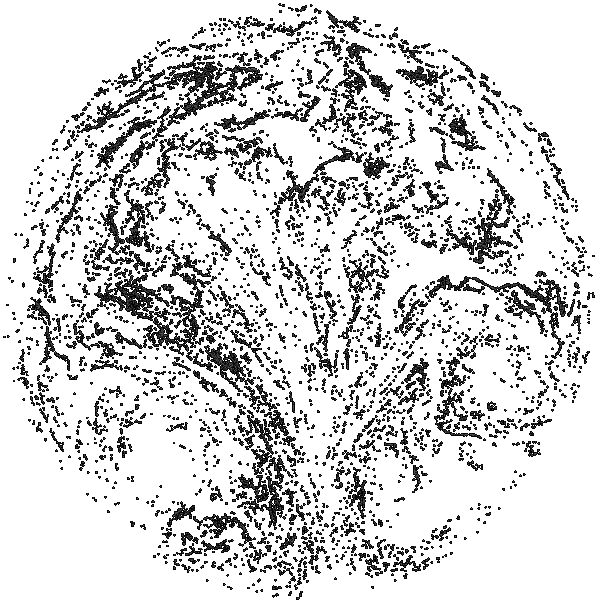

Supplement: S1 Fig — (ZIP) [file pone.0211413.s002.zip › Test images/S1_20.bmp]

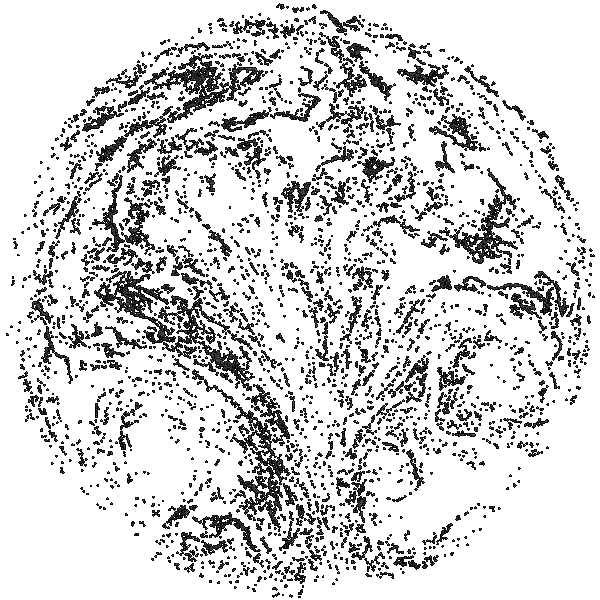

Supplement: S1 Fig — (ZIP) [file pone.0211413.s002.zip › Test images/S1_21.bmp]

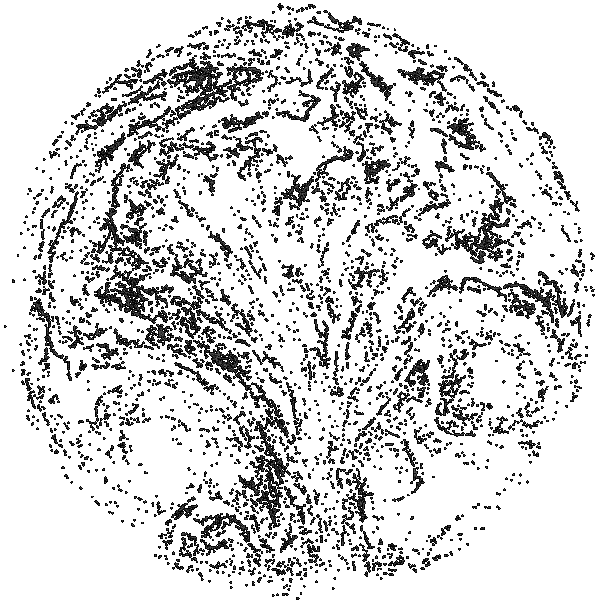

Supplement: S1 Fig — (ZIP) [file pone.0211413.s002.zip › Test images/S1_22.bmp]

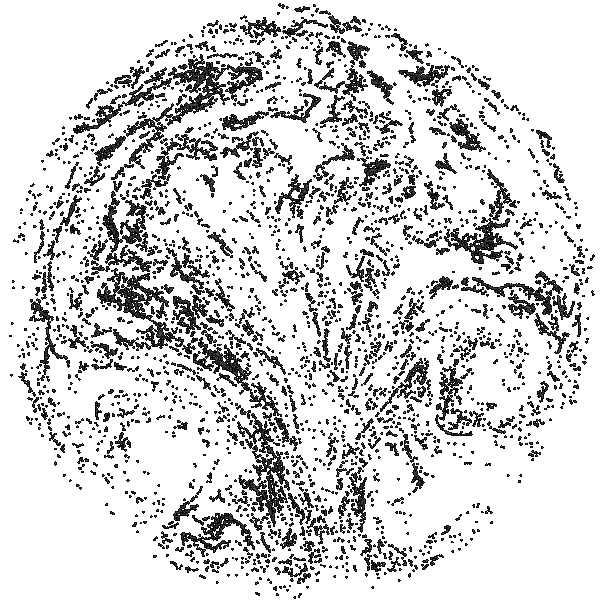

Supplement: S1 Fig — (ZIP) [file pone.0211413.s002.zip › Test images/S1_23.bmp]

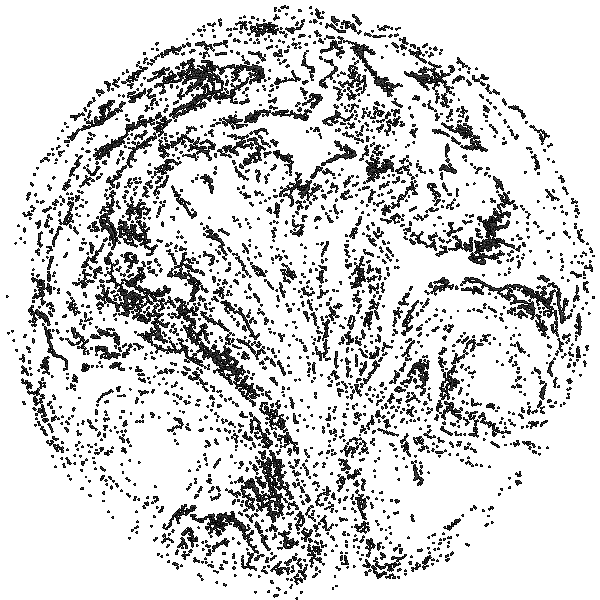

Supplement: S1 Fig — (ZIP) [file pone.0211413.s002.zip › Test images/S1_24.bmp]

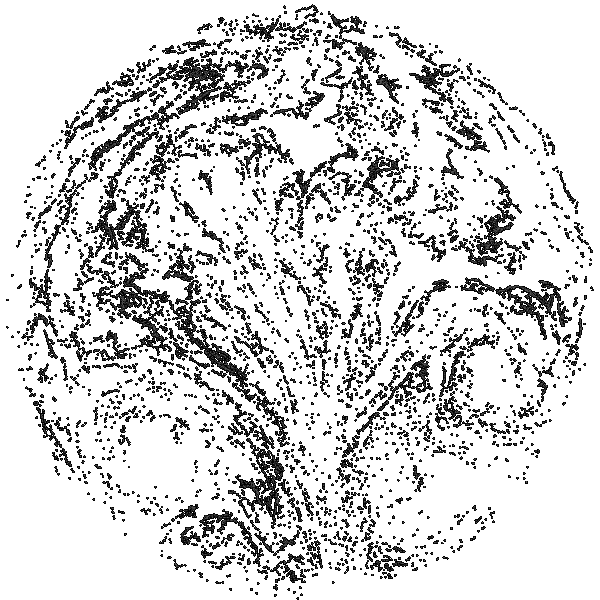

Supplement: S1 Fig — (ZIP) [file pone.0211413.s002.zip › Test images/S1_25.bmp]

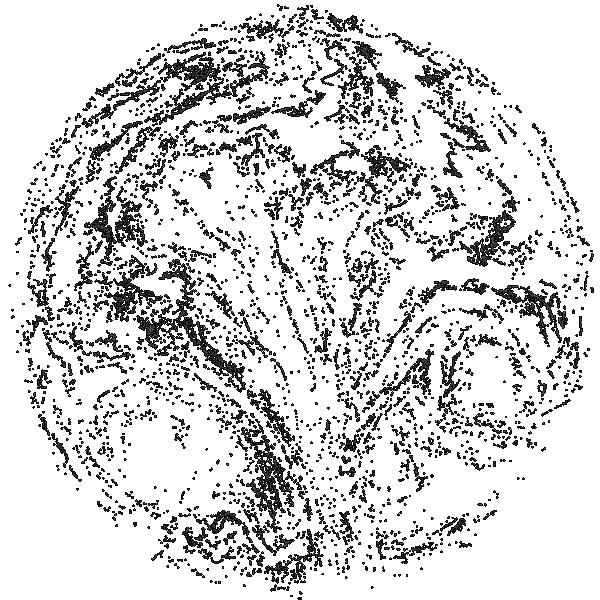

Supplement: S1 Fig — (ZIP) [file pone.0211413.s002.zip › Test images/S1_26.bmp]

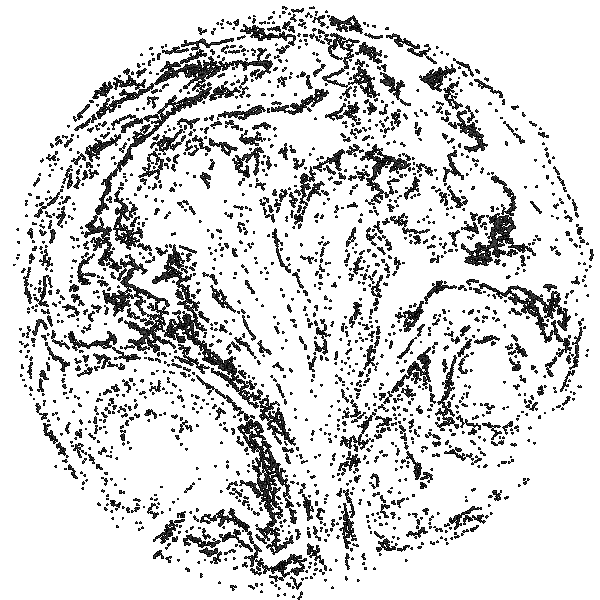

Supplement: S1 Fig — (ZIP) [file pone.0211413.s002.zip › Test images/S1_27.bmp]

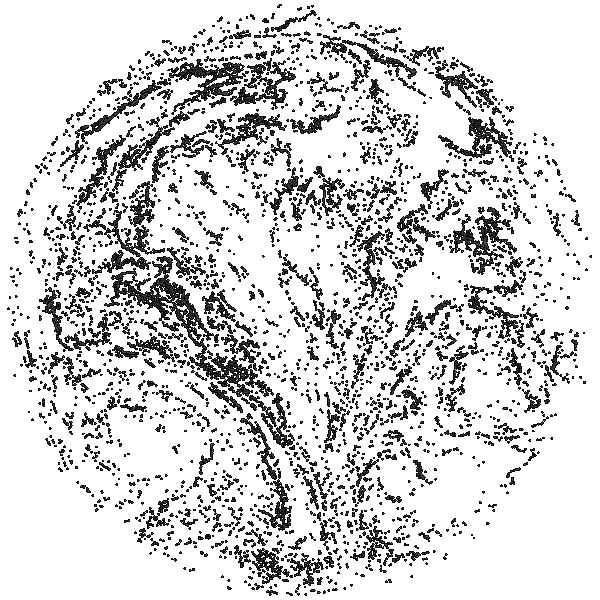

Supplement: S1 Fig — (ZIP) [file pone.0211413.s002.zip › Test images/S2_01.bmp]

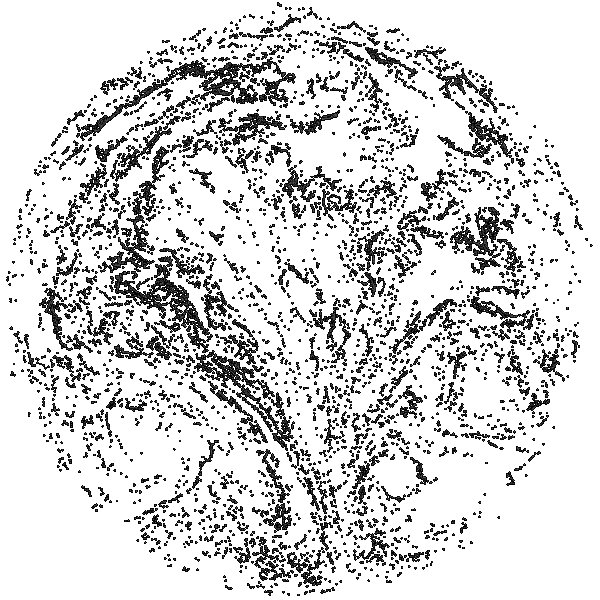

Supplement: S1 Fig — (ZIP) [file pone.0211413.s002.zip › Test images/S2_02.bmp]

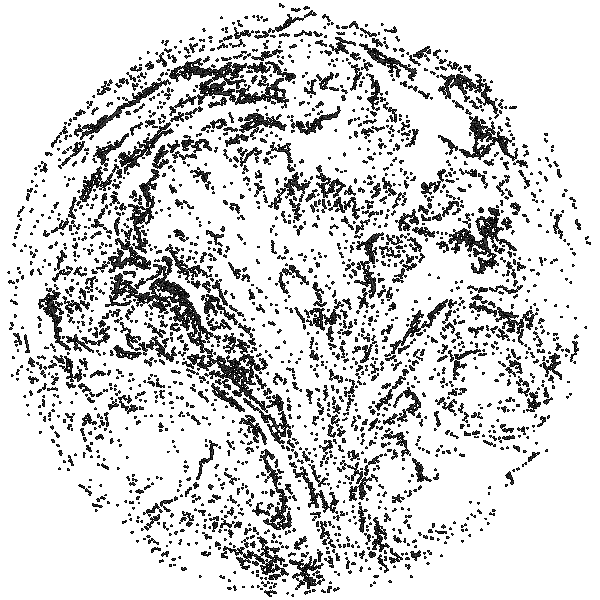

Supplement: S1 Fig — (ZIP) [file pone.0211413.s002.zip › Test images/S2_03.bmp]

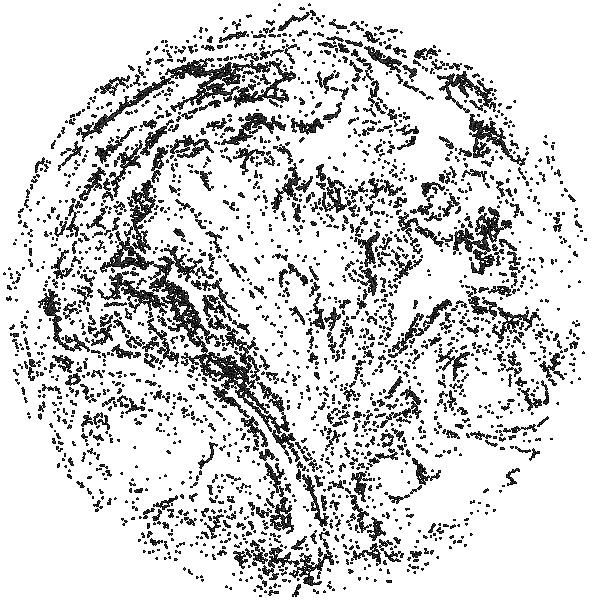

Supplement: S1 Fig — (ZIP) [file pone.0211413.s002.zip › Test images/S2_04.bmp]

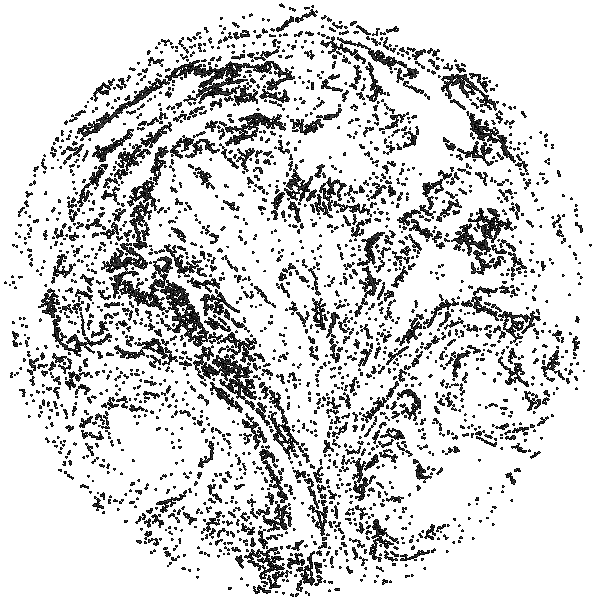

Supplement: S1 Fig — (ZIP) [file pone.0211413.s002.zip › Test images/S2_05.bmp]

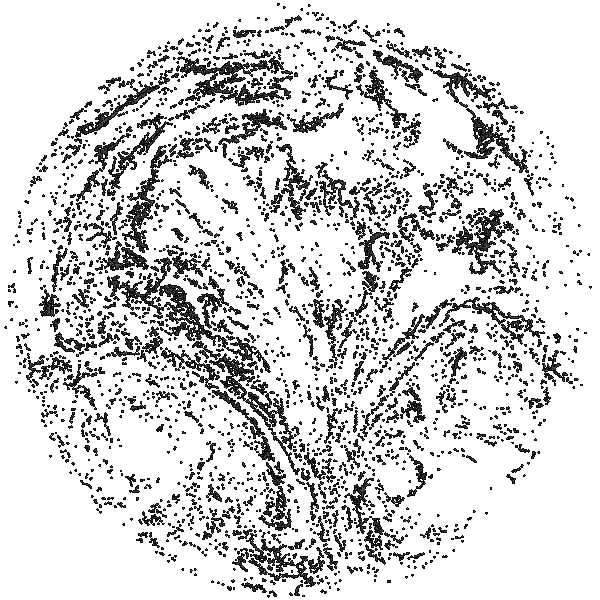

Supplement: S1 Fig — (ZIP) [file pone.0211413.s002.zip › Test images/S2_06.bmp]

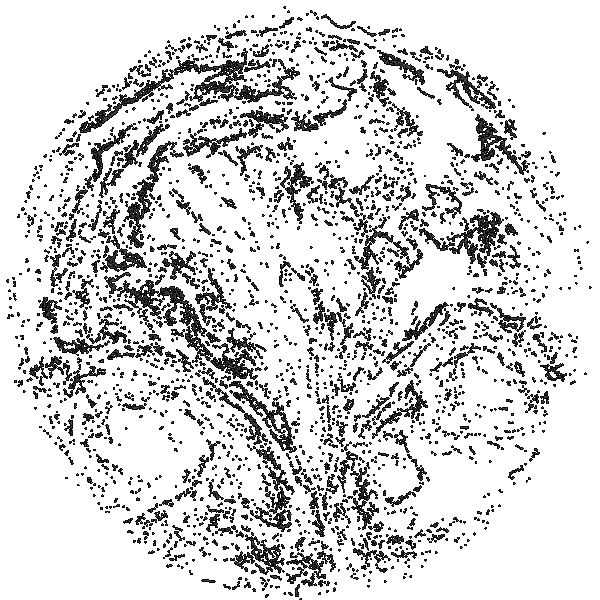

Supplement: S1 Fig — (ZIP) [file pone.0211413.s002.zip › Test images/S2_07.bmp]

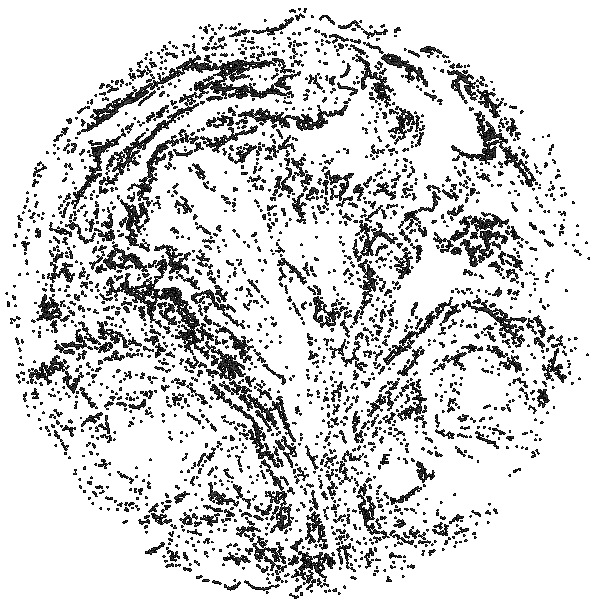

Supplement: S1 Fig — (ZIP) [file pone.0211413.s002.zip › Test images/S2_08.bmp]

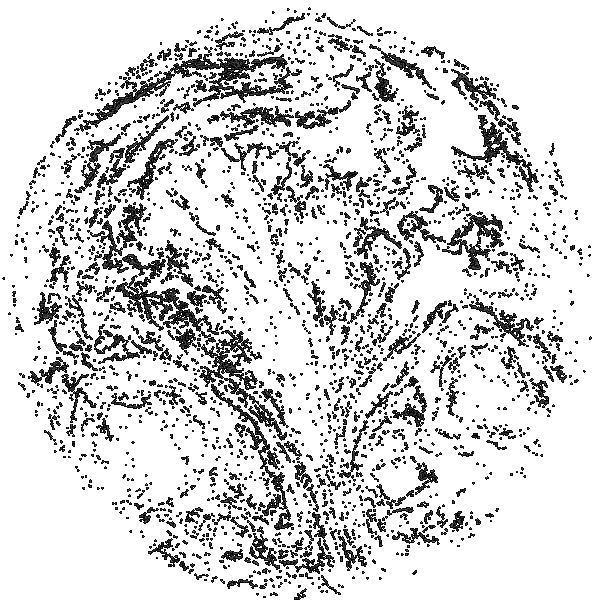

Supplement: S1 Fig — (ZIP) [file pone.0211413.s002.zip › Test images/S2_09.bmp]

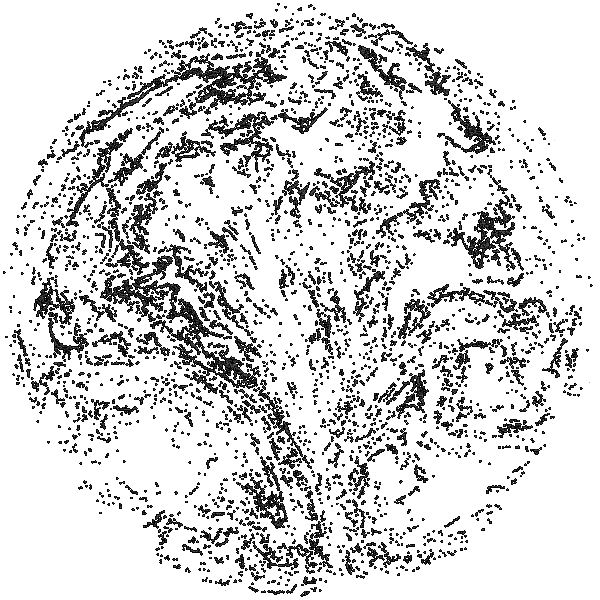

Supplement: S1 Fig — (ZIP) [file pone.0211413.s002.zip › Test images/S2_10.bmp]

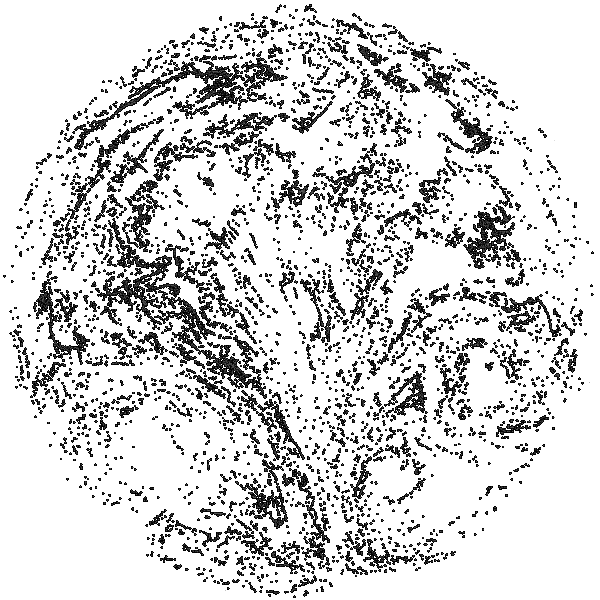

Supplement: S1 Fig — (ZIP) [file pone.0211413.s002.zip › Test images/S2_11.bmp]

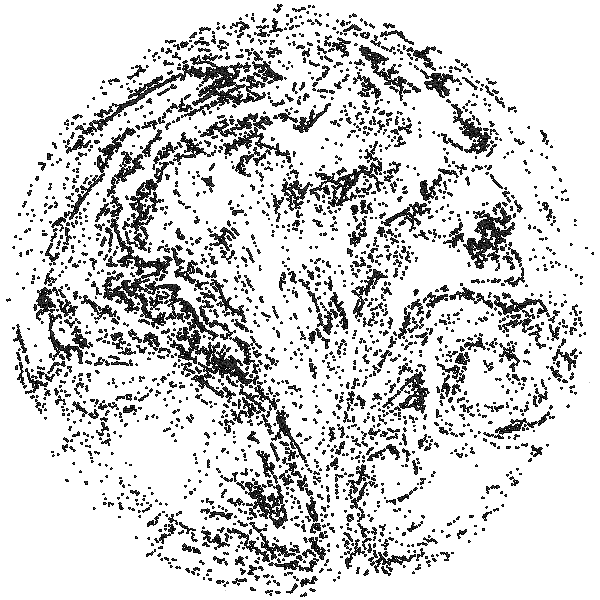

Supplement: S1 Fig — (ZIP) [file pone.0211413.s002.zip › Test images/S2_12.bmp]

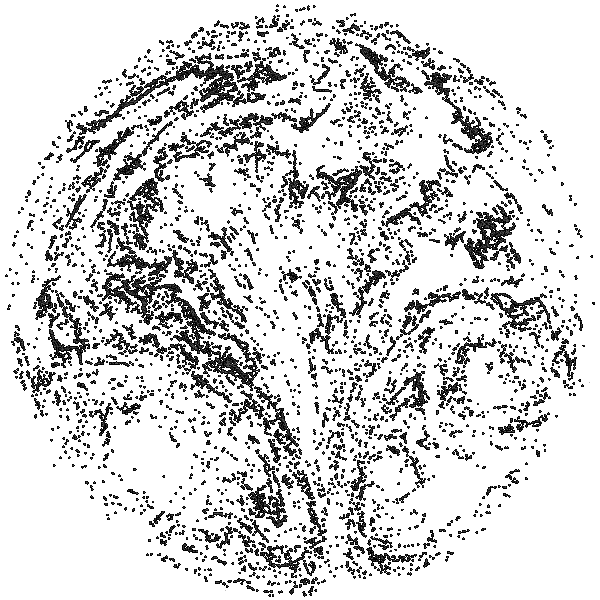

Supplement: S1 Fig — (ZIP) [file pone.0211413.s002.zip › Test images/S2_13.bmp]

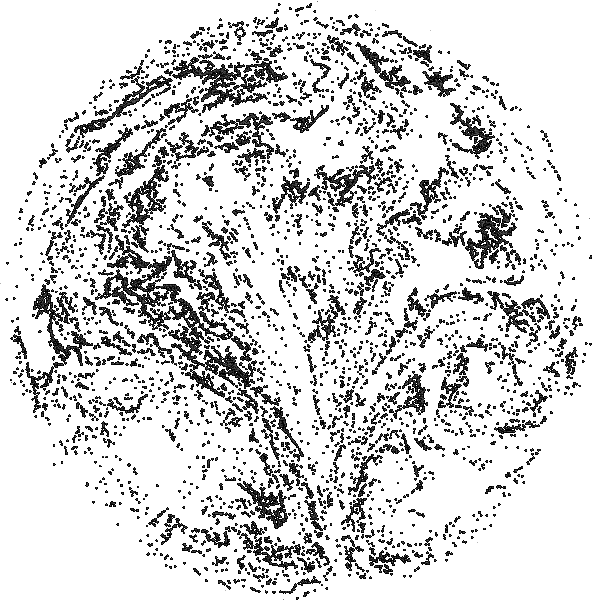

Supplement: S1 Fig — (ZIP) [file pone.0211413.s002.zip › Test images/S2_14.bmp]

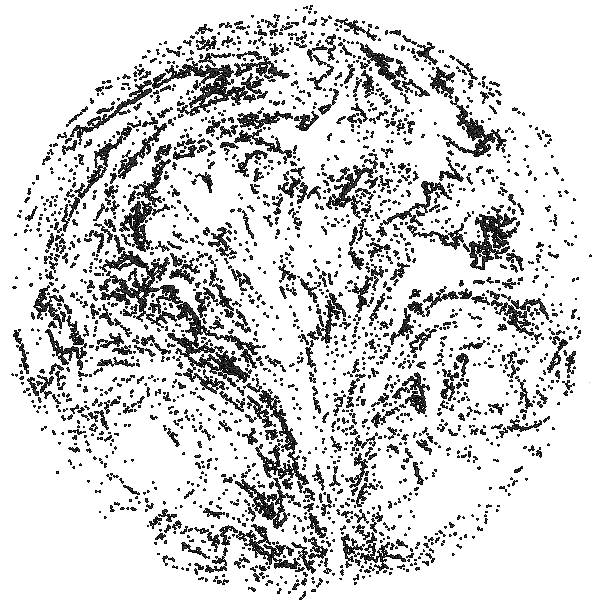

Supplement: S1 Fig — (ZIP) [file pone.0211413.s002.zip › Test images/S2_15.bmp]

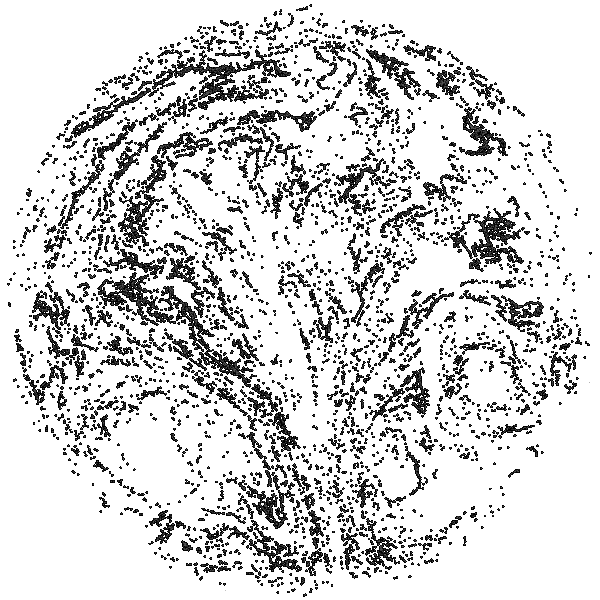

Supplement: S1 Fig — (ZIP) [file pone.0211413.s002.zip › Test images/S2_16.bmp]

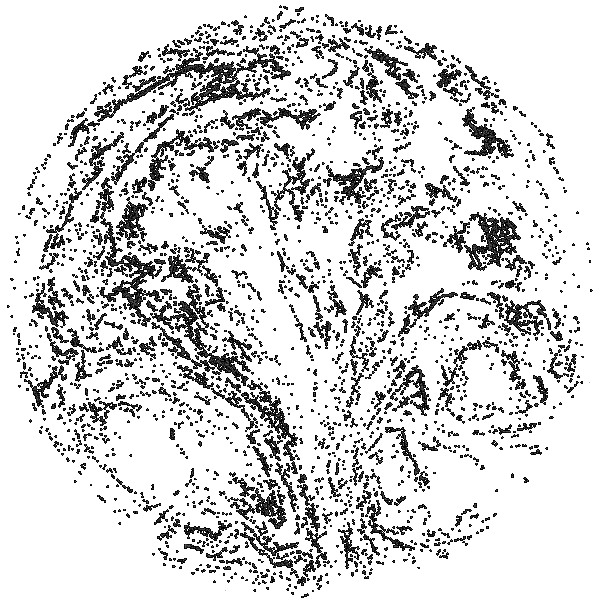

Supplement: S1 Fig — (ZIP) [file pone.0211413.s002.zip › Test images/S2_17.bmp]

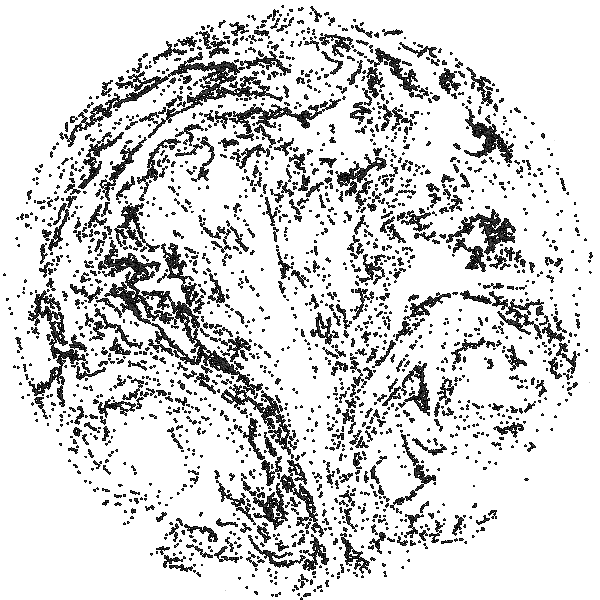

Supplement: S1 Fig — (ZIP) [file pone.0211413.s002.zip › Test images/S2_18.bmp]

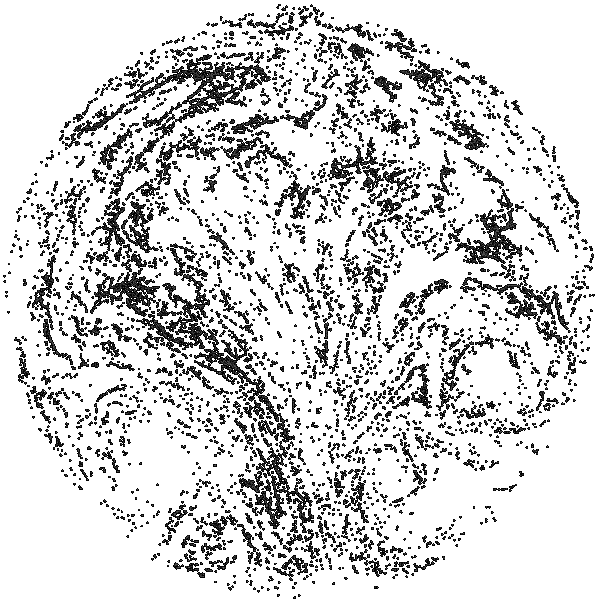

Supplement: S1 Fig — (ZIP) [file pone.0211413.s002.zip › Test images/S2_19.bmp]

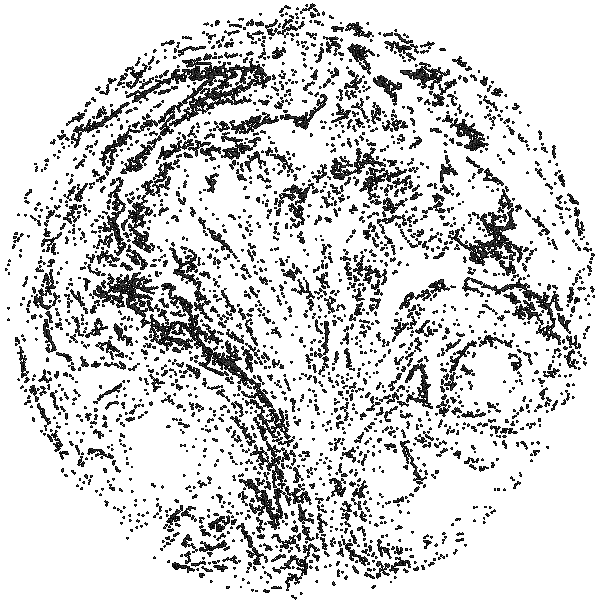

Supplement: S1 Fig — (ZIP) [file pone.0211413.s002.zip › Test images/S2_20.bmp]

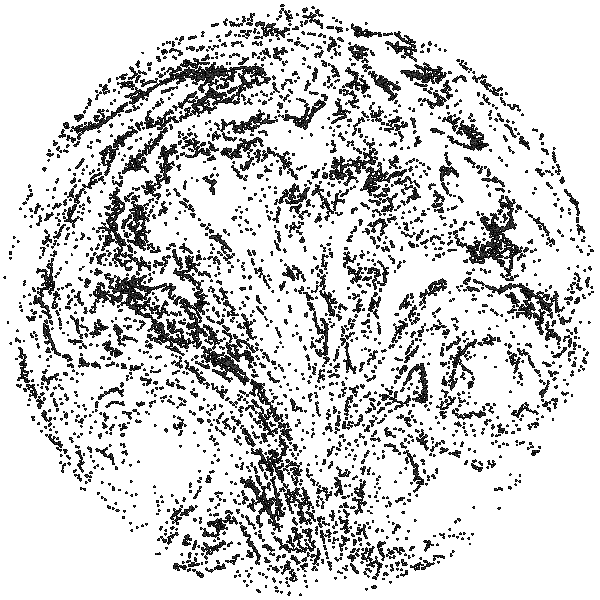

Supplement: S1 Fig — (ZIP) [file pone.0211413.s002.zip › Test images/S2_21.bmp]

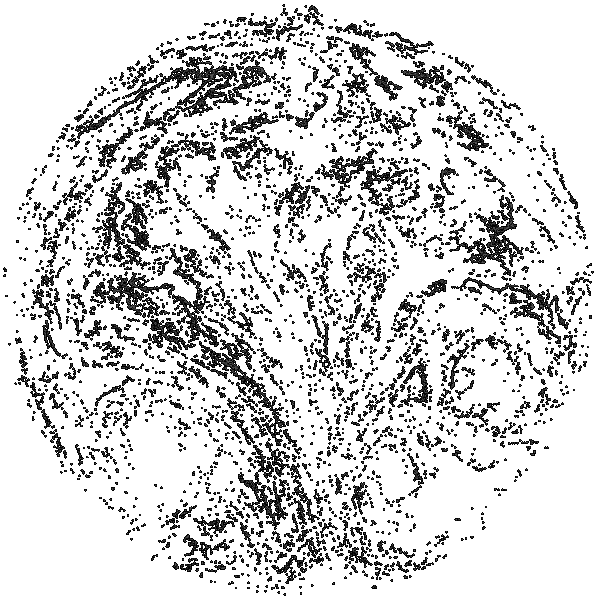

Supplement: S1 Fig — (ZIP) [file pone.0211413.s002.zip › Test images/S2_22.bmp]

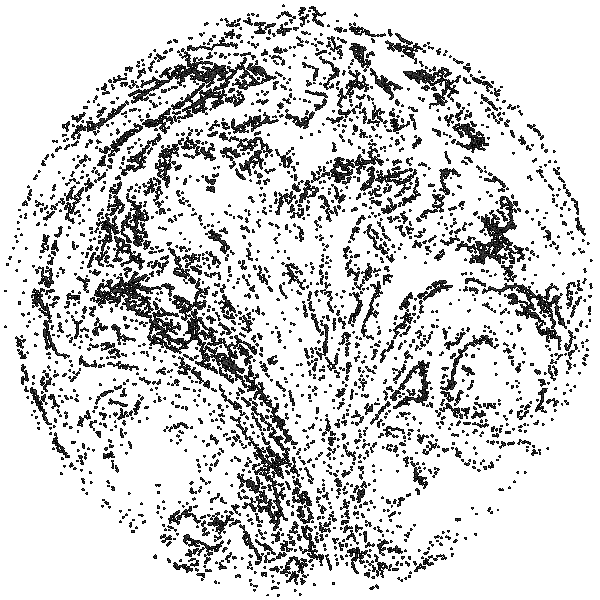

Supplement: S1 Fig — (ZIP) [file pone.0211413.s002.zip › Test images/S2_23.bmp]

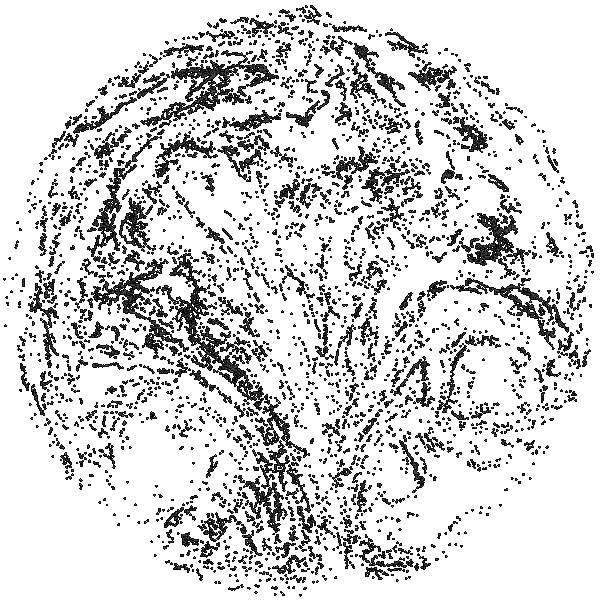

Supplement: S1 Fig — (ZIP) [file pone.0211413.s002.zip › Test images/S2_24.bmp]

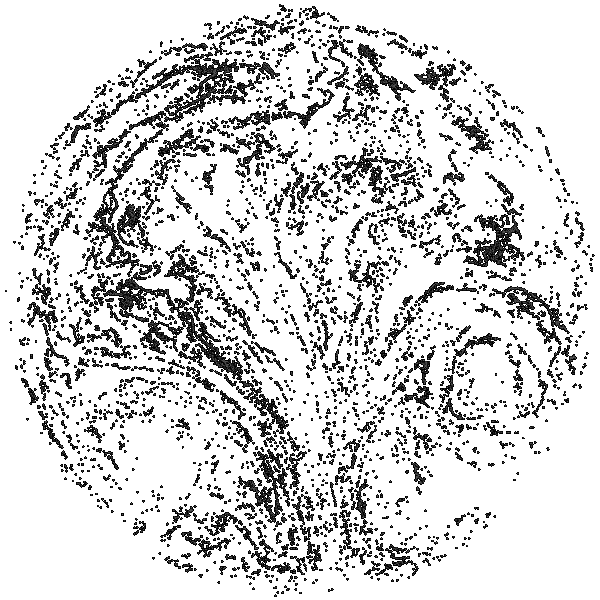

Supplement: S1 Fig — (ZIP) [file pone.0211413.s002.zip › Test images/S2_25.bmp]

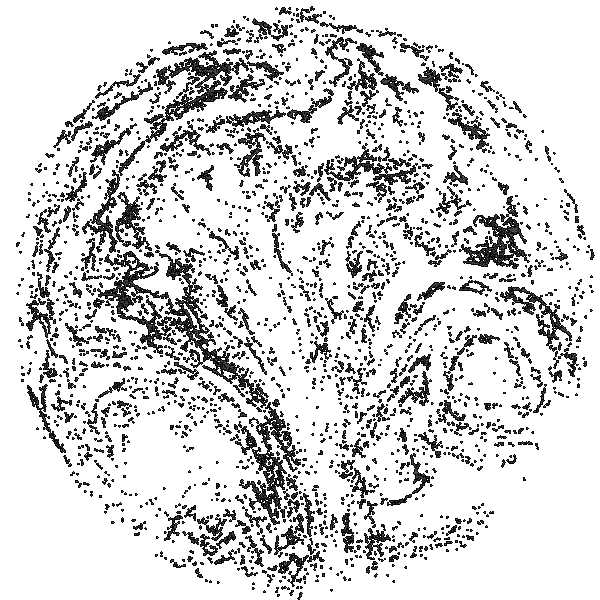

Supplement: S1 Fig — (ZIP) [file pone.0211413.s002.zip › Test images/S2_26.bmp]

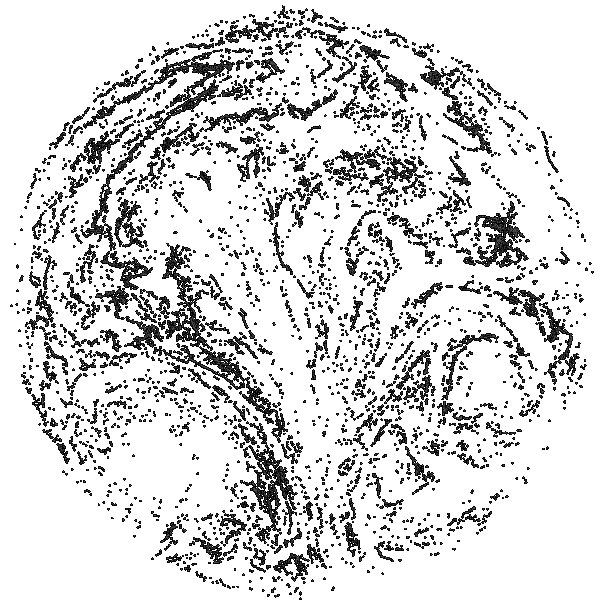

Supplement: S1 Fig — (ZIP) [file pone.0211413.s002.zip › Test images/S2_27.bmp]

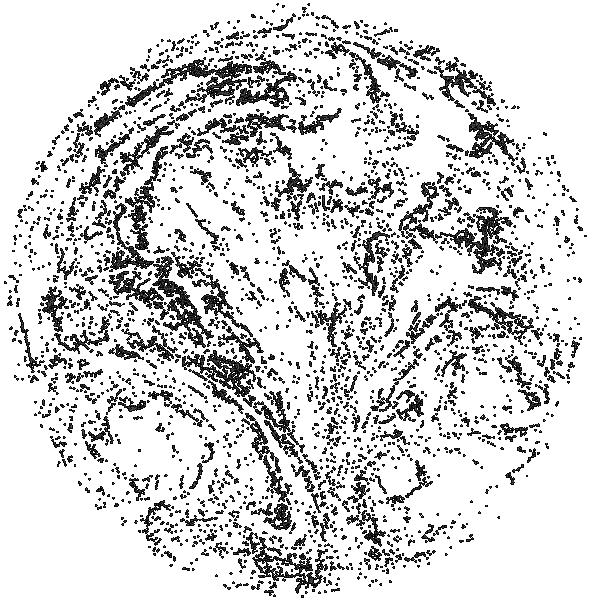

Supplement: S1 Fig — (ZIP) [file pone.0211413.s002.zip › Test images/S3_01.bmp]

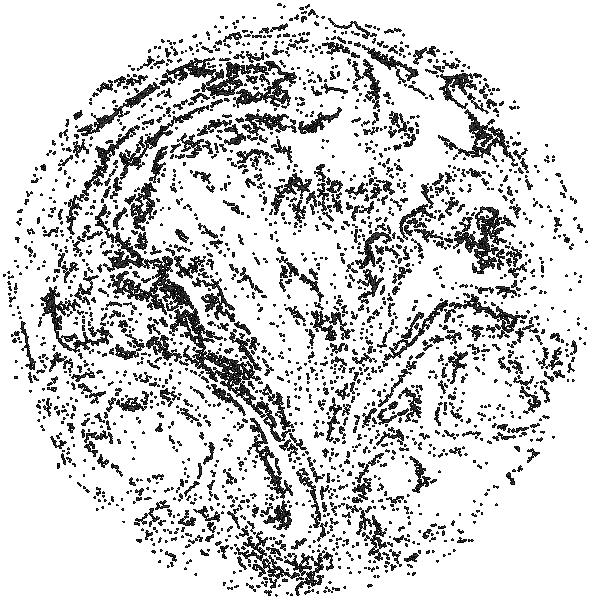

Supplement: S1 Fig — (ZIP) [file pone.0211413.s002.zip › Test images/S3_02.bmp]

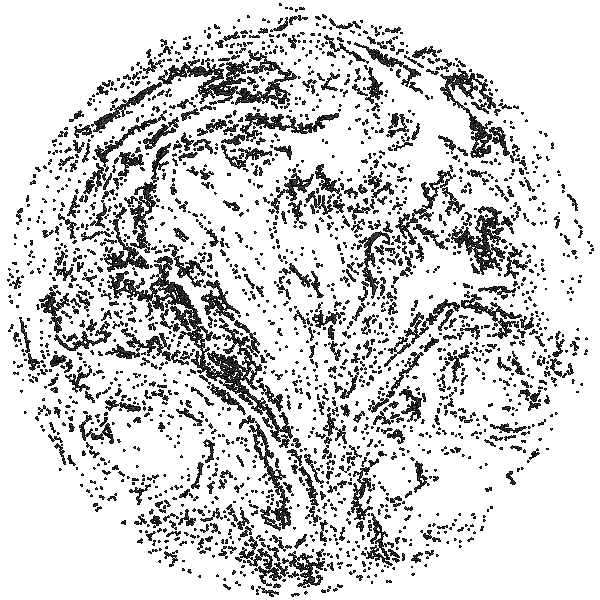

Supplement: S1 Fig — (ZIP) [file pone.0211413.s002.zip › Test images/S3_03.bmp]

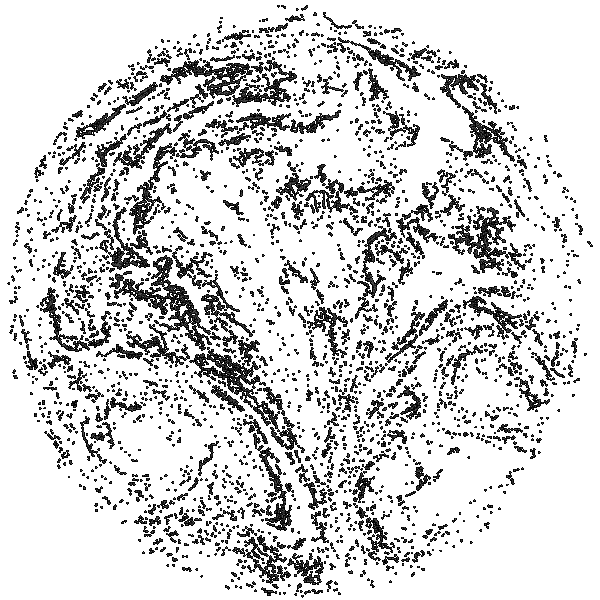

Supplement: S1 Fig — (ZIP) [file pone.0211413.s002.zip › Test images/S3_04.bmp]

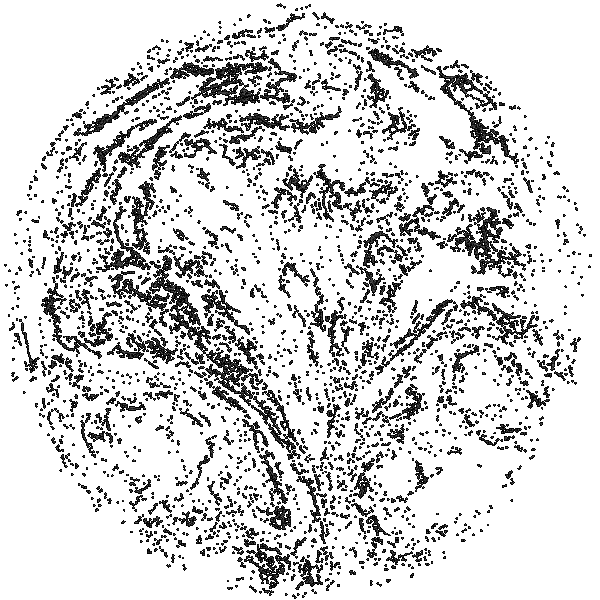

Supplement: S1 Fig — (ZIP) [file pone.0211413.s002.zip › Test images/S3_05.bmp]

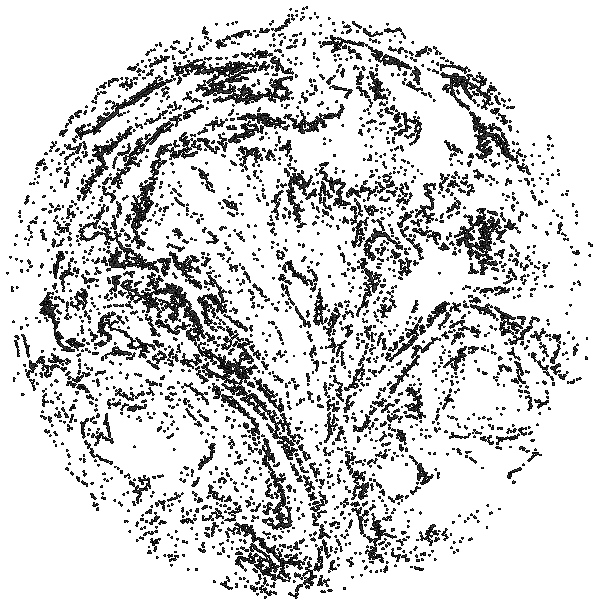

Supplement: S1 Fig — (ZIP) [file pone.0211413.s002.zip › Test images/S3_06.bmp]

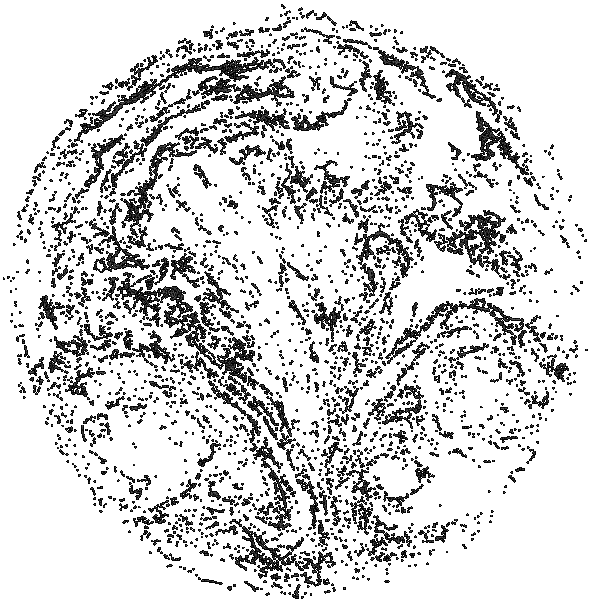

Supplement: S1 Fig — (ZIP) [file pone.0211413.s002.zip › Test images/S3_07.bmp]

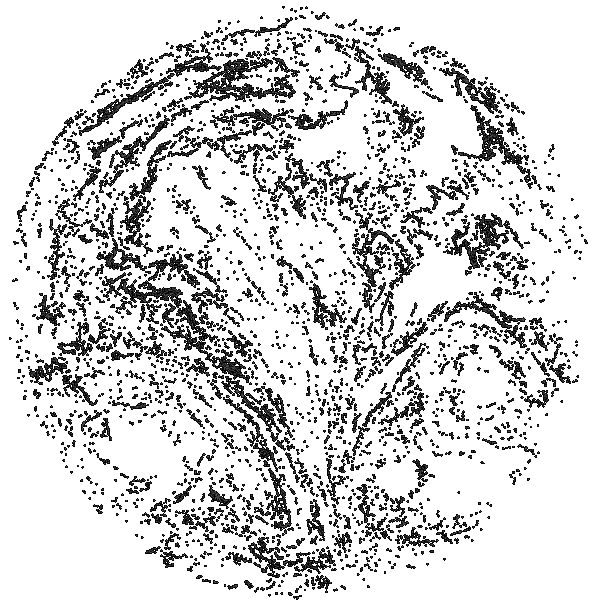

Supplement: S1 Fig — (ZIP) [file pone.0211413.s002.zip › Test images/S3_08.bmp]

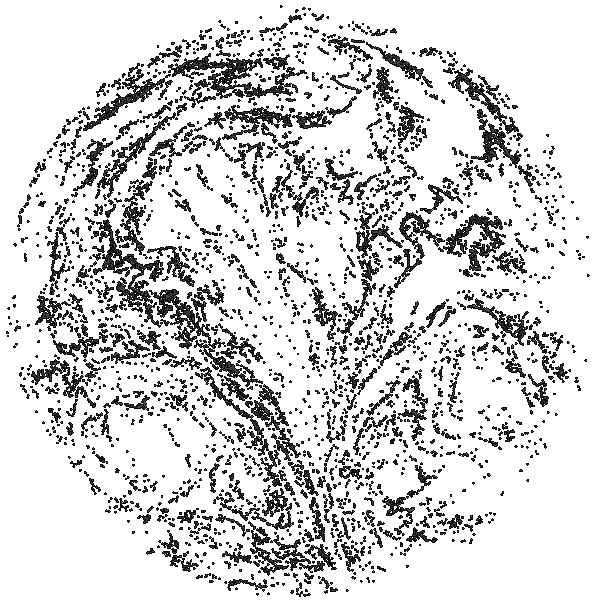

Supplement: S1 Fig — (ZIP) [file pone.0211413.s002.zip › Test images/S3_09.bmp]

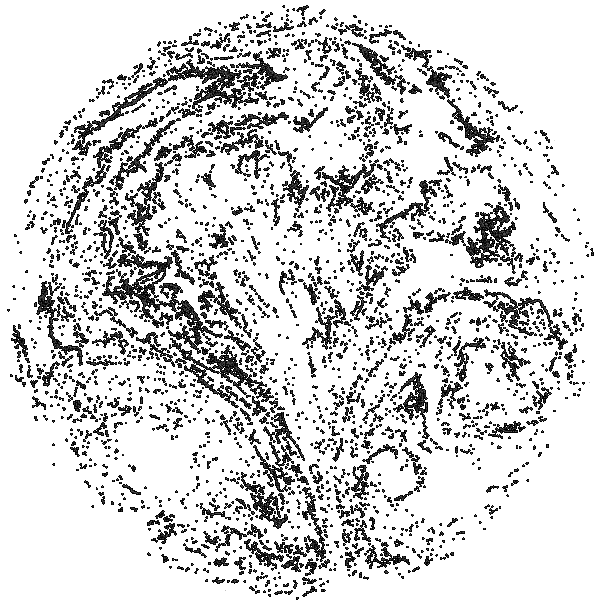

Supplement: S1 Fig — (ZIP) [file pone.0211413.s002.zip › Test images/S3_10.bmp]

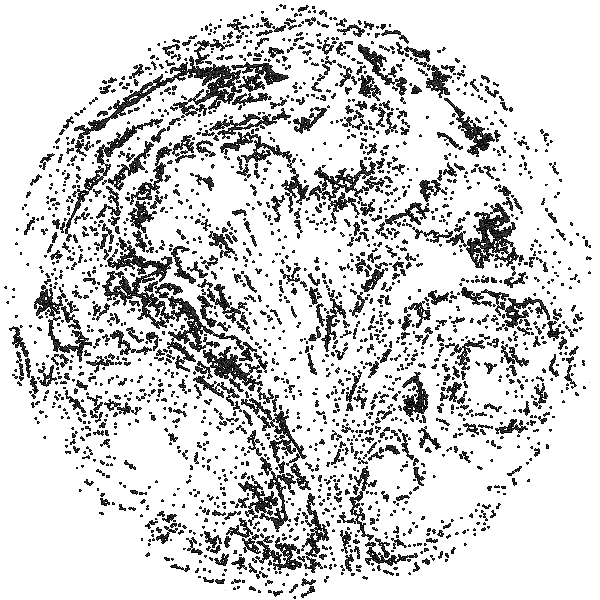

Supplement: S1 Fig — (ZIP) [file pone.0211413.s002.zip › Test images/S3_11.bmp]

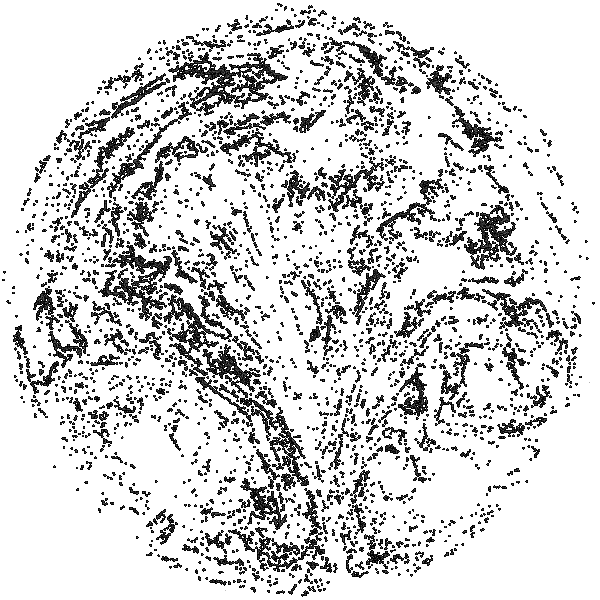

Supplement: S1 Fig — (ZIP) [file pone.0211413.s002.zip › Test images/S3_12.bmp]

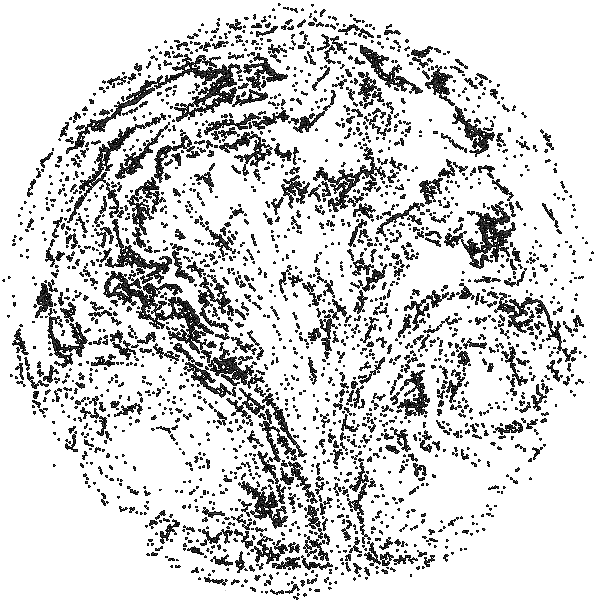

Supplement: S1 Fig — (ZIP) [file pone.0211413.s002.zip › Test images/S3_13.bmp]

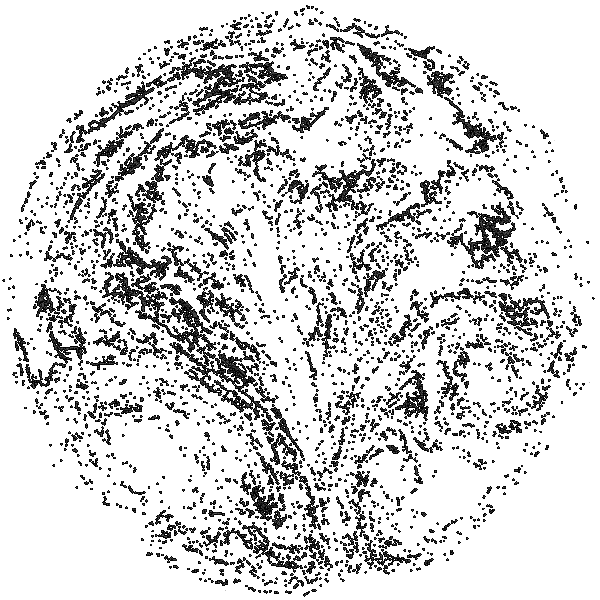

Supplement: S1 Fig — (ZIP) [file pone.0211413.s002.zip › Test images/S3_14.bmp]

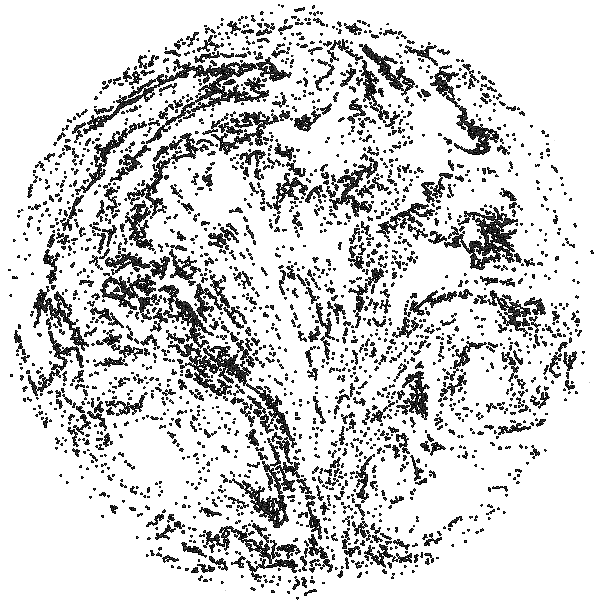

Supplement: S1 Fig — (ZIP) [file pone.0211413.s002.zip › Test images/S3_15.bmp]

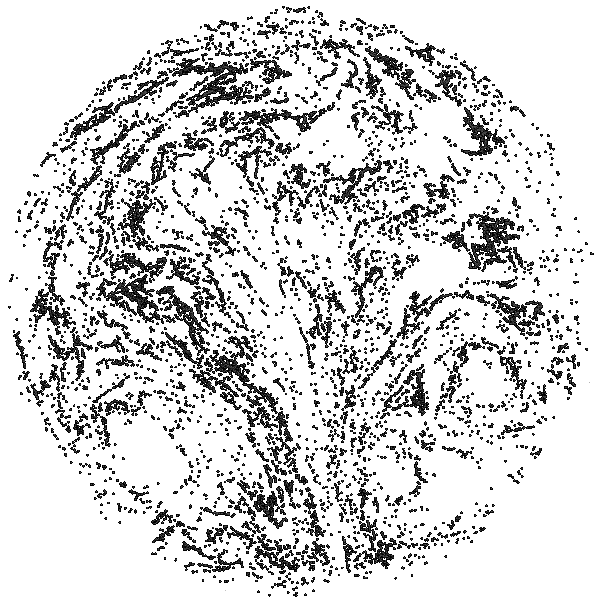

Supplement: S1 Fig — (ZIP) [file pone.0211413.s002.zip › Test images/S3_16.bmp]

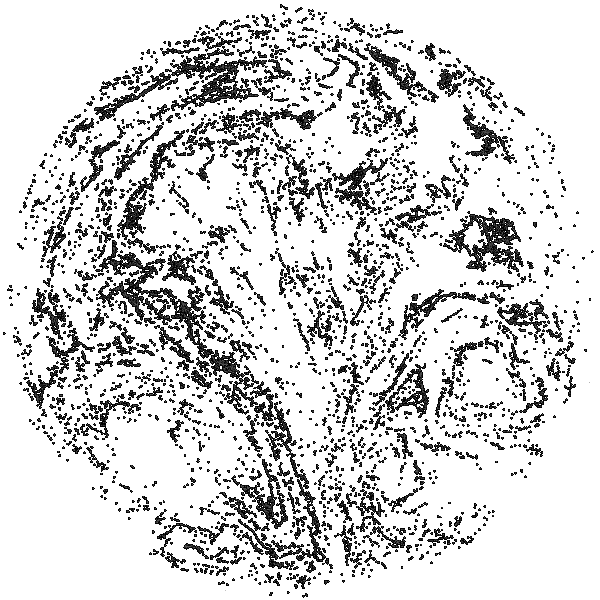

Supplement: S1 Fig — (ZIP) [file pone.0211413.s002.zip › Test images/S3_17.bmp]

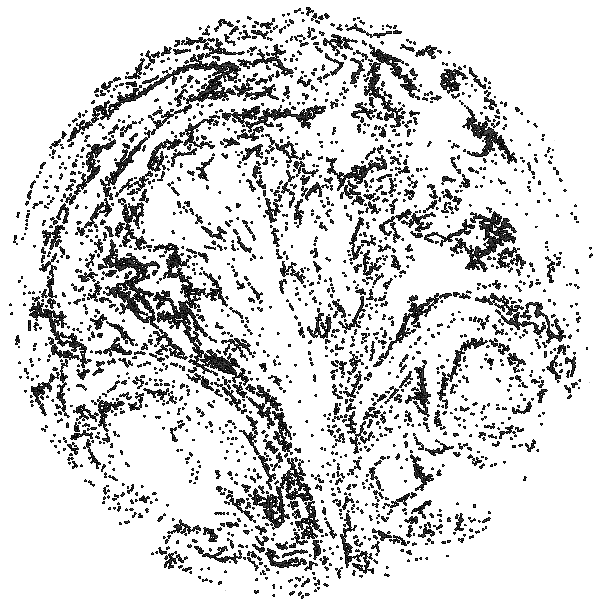

Supplement: S1 Fig — (ZIP) [file pone.0211413.s002.zip › Test images/S3_18.bmp]
