# Supplementary figures and images for: Correlating exhaled aerosol images to small airway obstructive diseases: A study with dynamic mode decomposition and machine learning (part 2 of 2)
Source: PLoS One. 2019 Jan 31;14(1):e0211413. doi: 10.1371/journal.pone.0211413 (PMC6354993; doi:10.1371/journal.pone.0211413)

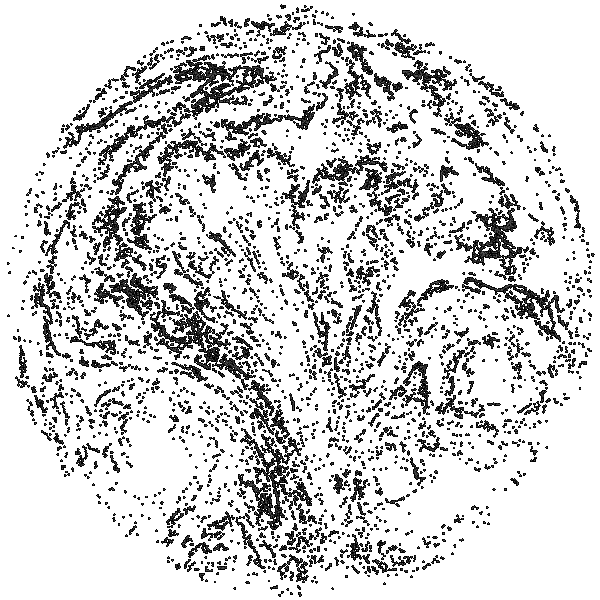

Supplement: S1 Fig — (ZIP) [file pone.0211413.s002.zip › Test images/S3_19.bmp]

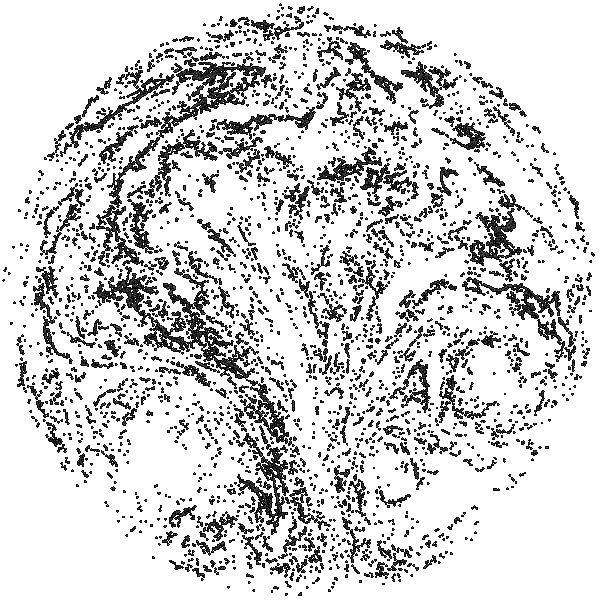

Supplement: S1 Fig — (ZIP) [file pone.0211413.s002.zip › Test images/S3_20.bmp]

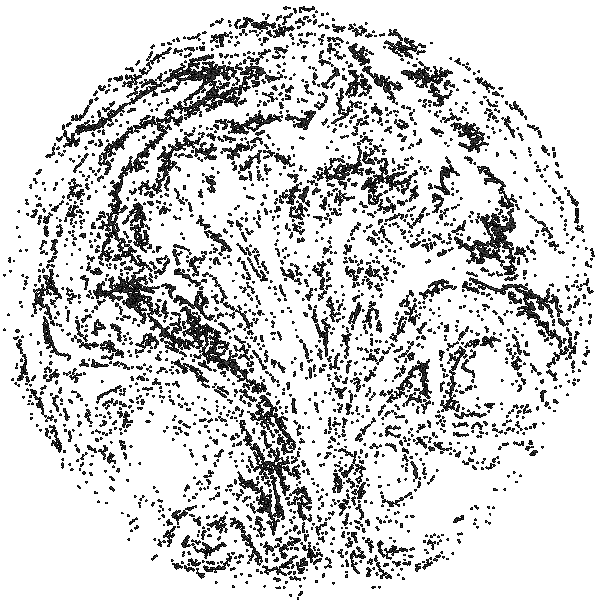

Supplement: S1 Fig — (ZIP) [file pone.0211413.s002.zip › Test images/S3_21.bmp]

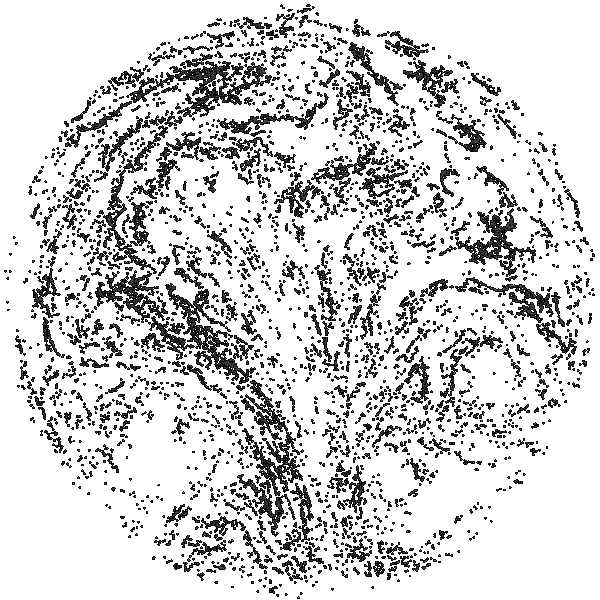

Supplement: S1 Fig — (ZIP) [file pone.0211413.s002.zip › Test images/S3_22.bmp]

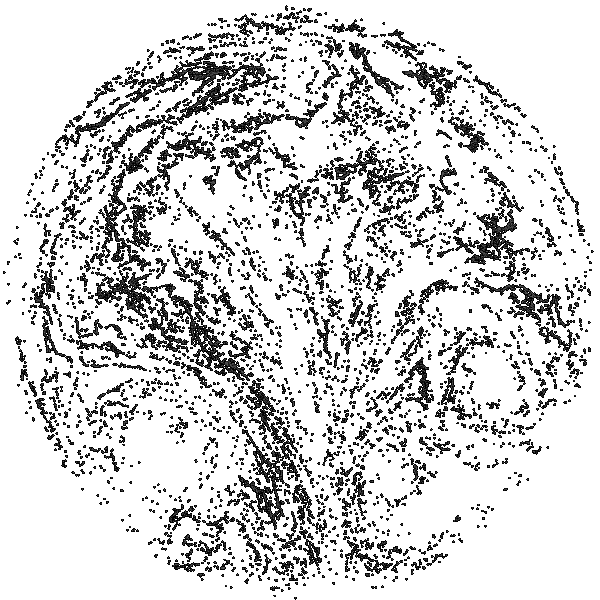

Supplement: S1 Fig — (ZIP) [file pone.0211413.s002.zip › Test images/S3_23.bmp]

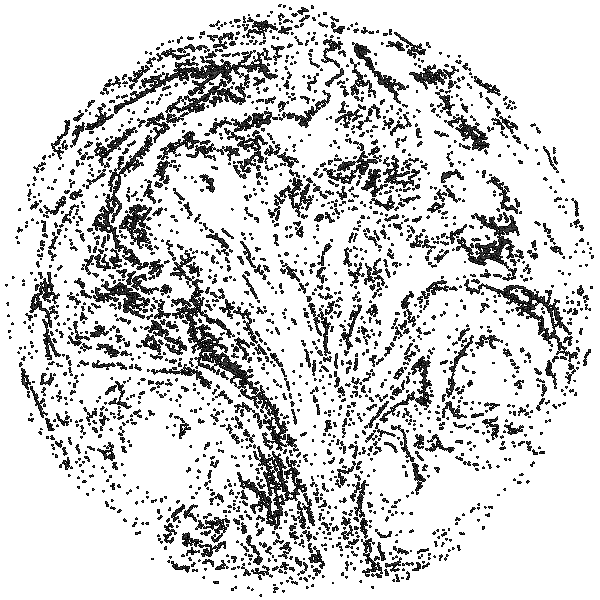

Supplement: S1 Fig — (ZIP) [file pone.0211413.s002.zip › Test images/S3_24.bmp]

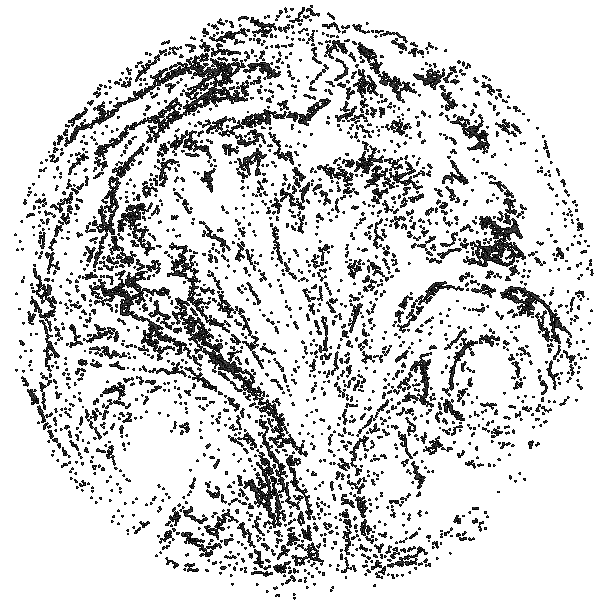

Supplement: S1 Fig — (ZIP) [file pone.0211413.s002.zip › Test images/S3_25.bmp]

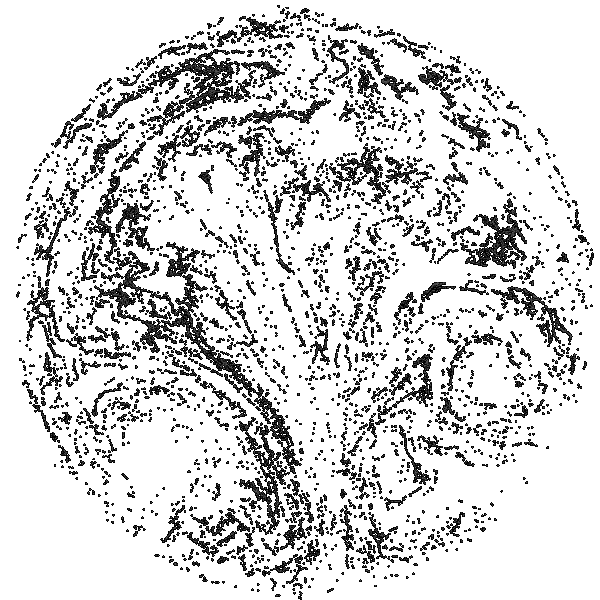

Supplement: S1 Fig — (ZIP) [file pone.0211413.s002.zip › Test images/S3_26.bmp]

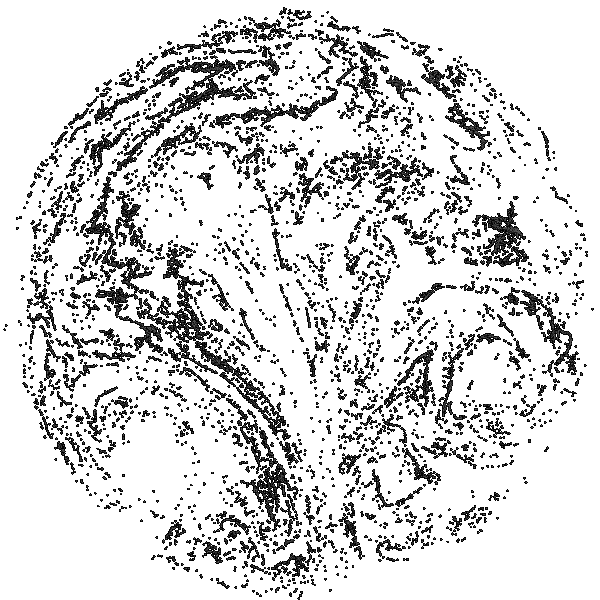

Supplement: S1 Fig — (ZIP) [file pone.0211413.s002.zip › Test images/S3_27.bmp]

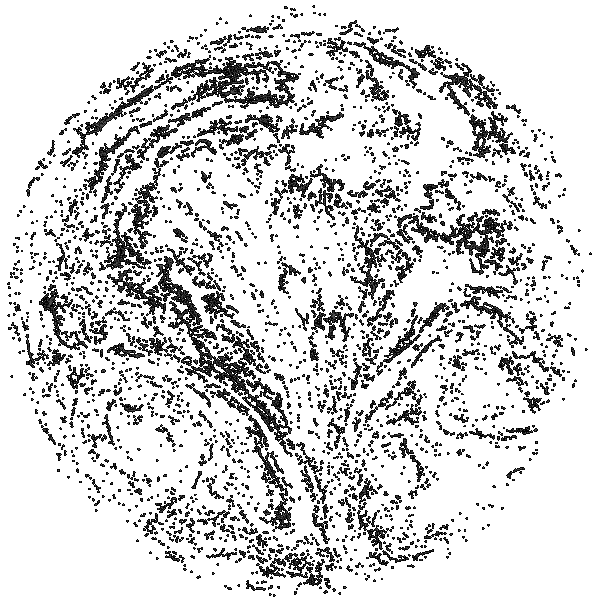

Supplement: S1 Fig — (ZIP) [file pone.0211413.s002.zip › Test images/S4_01.bmp]

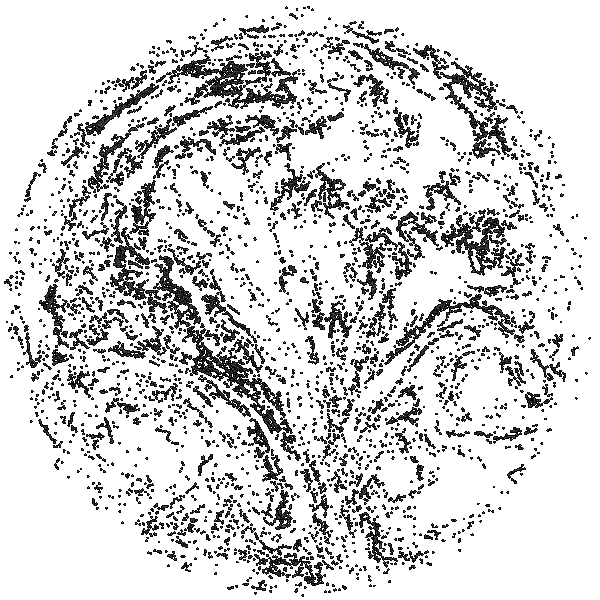

Supplement: S1 Fig — (ZIP) [file pone.0211413.s002.zip › Test images/S4_02.bmp]

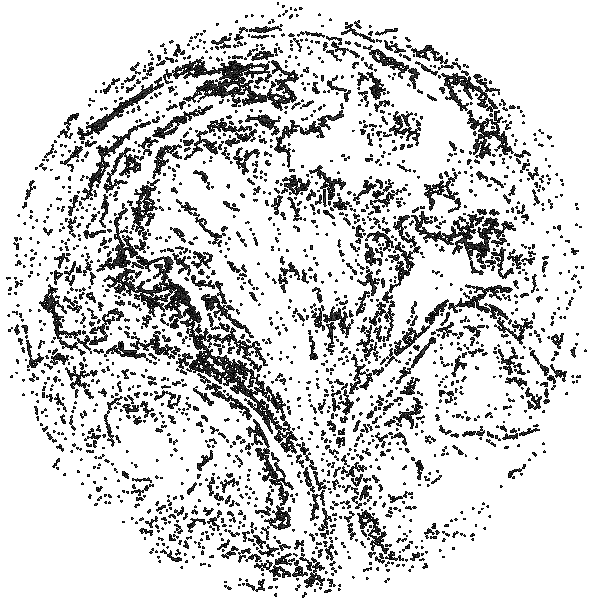

Supplement: S1 Fig — (ZIP) [file pone.0211413.s002.zip › Test images/S4_03.bmp]

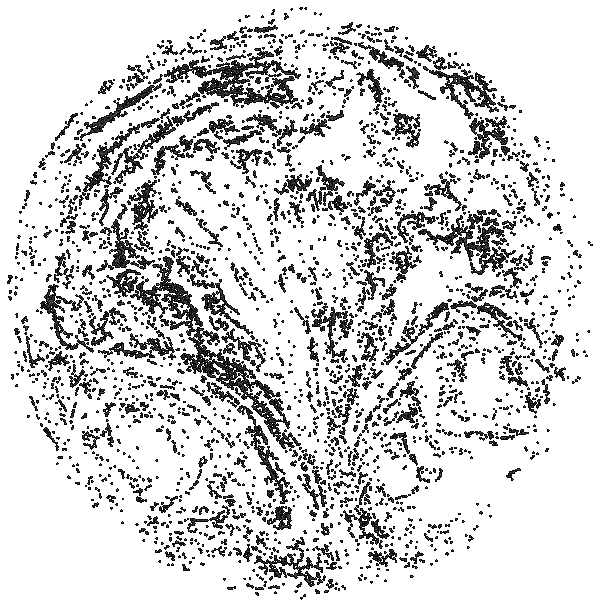

Supplement: S1 Fig — (ZIP) [file pone.0211413.s002.zip › Test images/S4_04.bmp]

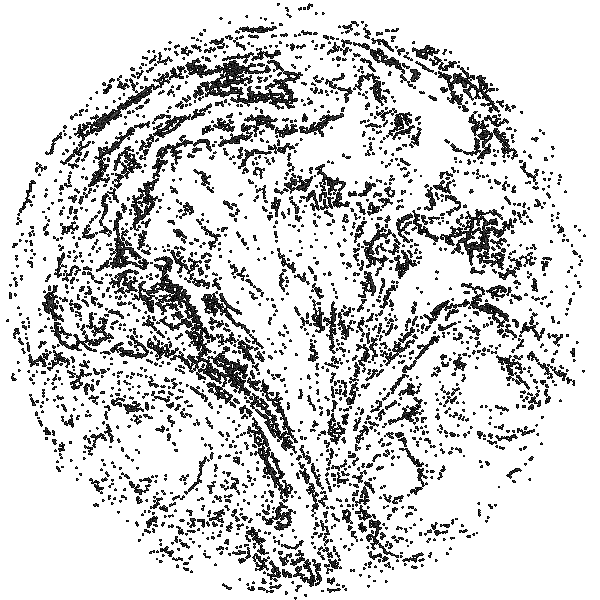

Supplement: S1 Fig — (ZIP) [file pone.0211413.s002.zip › Test images/S4_05.bmp]

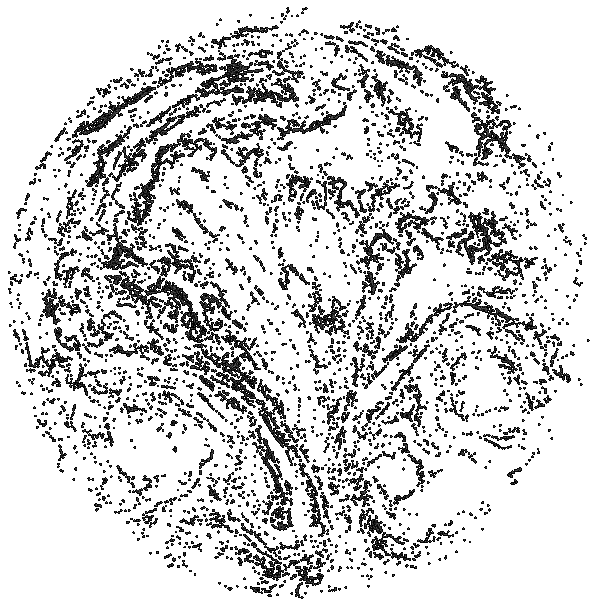

Supplement: S1 Fig — (ZIP) [file pone.0211413.s002.zip › Test images/S4_06.bmp]

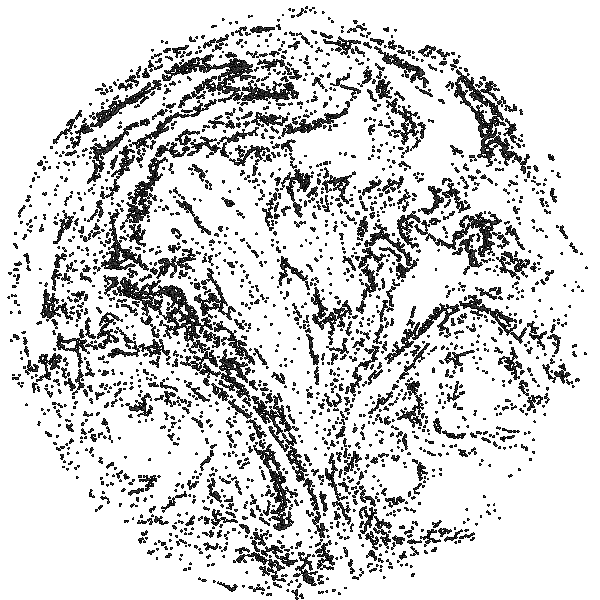

Supplement: S1 Fig — (ZIP) [file pone.0211413.s002.zip › Test images/S4_07.bmp]

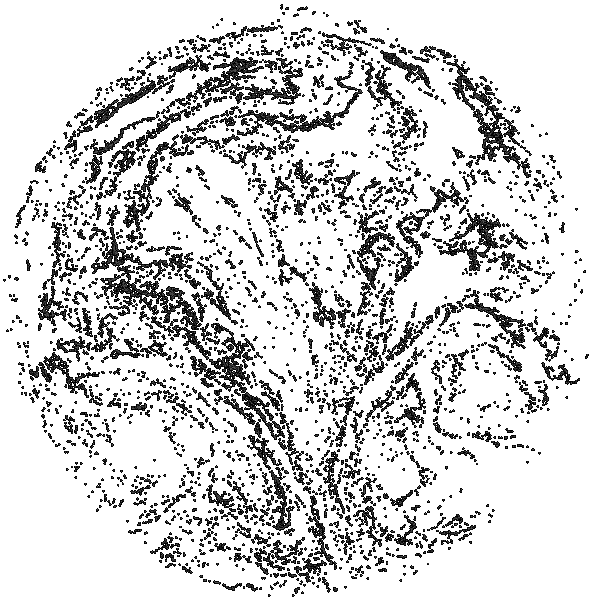

Supplement: S1 Fig — (ZIP) [file pone.0211413.s002.zip › Test images/S4_08.bmp]

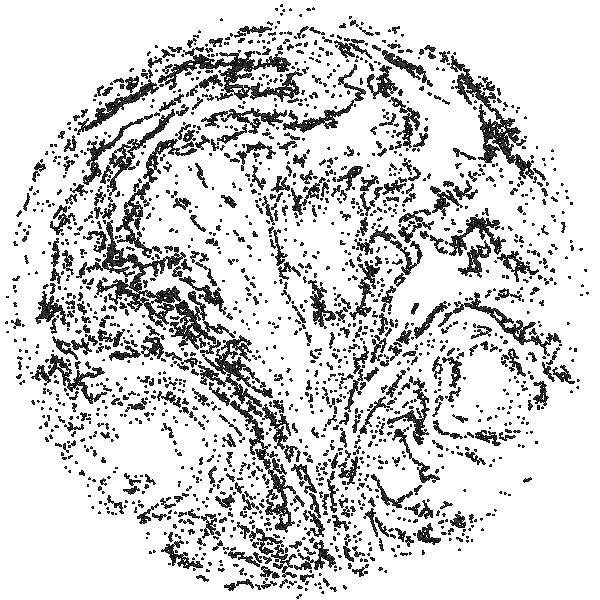

Supplement: S1 Fig — (ZIP) [file pone.0211413.s002.zip › Test images/S4_09.bmp]

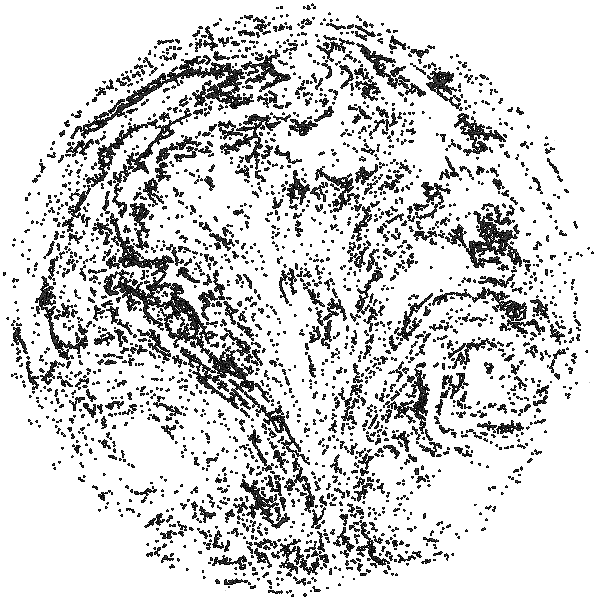

Supplement: S1 Fig — (ZIP) [file pone.0211413.s002.zip › Test images/S4_10.bmp]

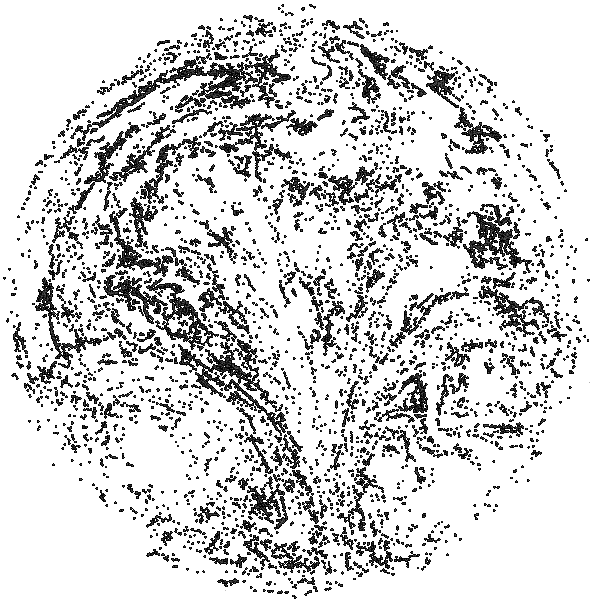

Supplement: S1 Fig — (ZIP) [file pone.0211413.s002.zip › Test images/S4_11.bmp]

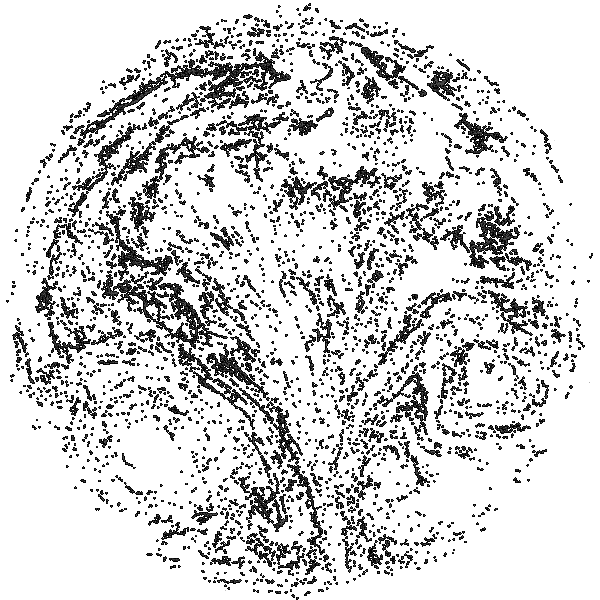

Supplement: S1 Fig — (ZIP) [file pone.0211413.s002.zip › Test images/S4_12.bmp]

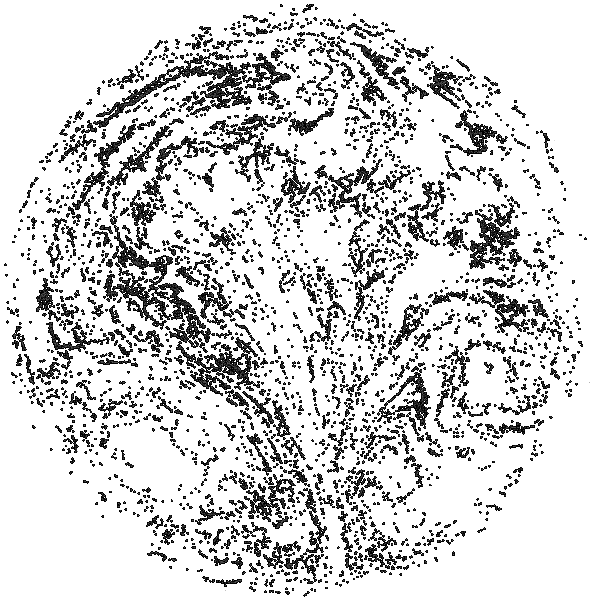

Supplement: S1 Fig — (ZIP) [file pone.0211413.s002.zip › Test images/S4_13.bmp]

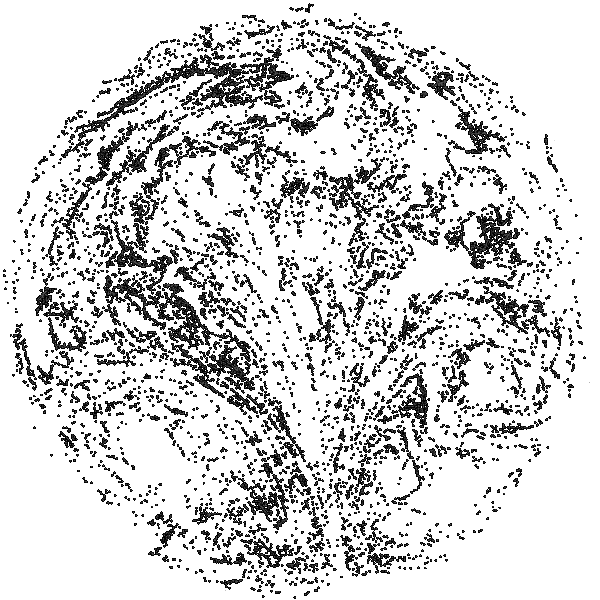

Supplement: S1 Fig — (ZIP) [file pone.0211413.s002.zip › Test images/S4_14.bmp]

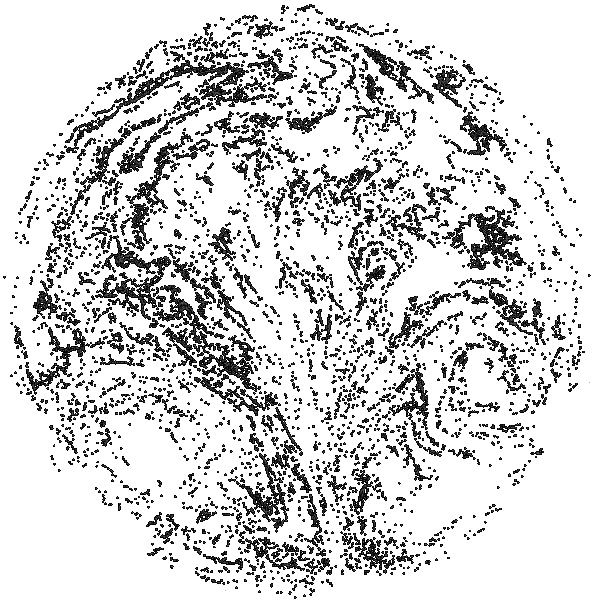

Supplement: S1 Fig — (ZIP) [file pone.0211413.s002.zip › Test images/S4_15.bmp]

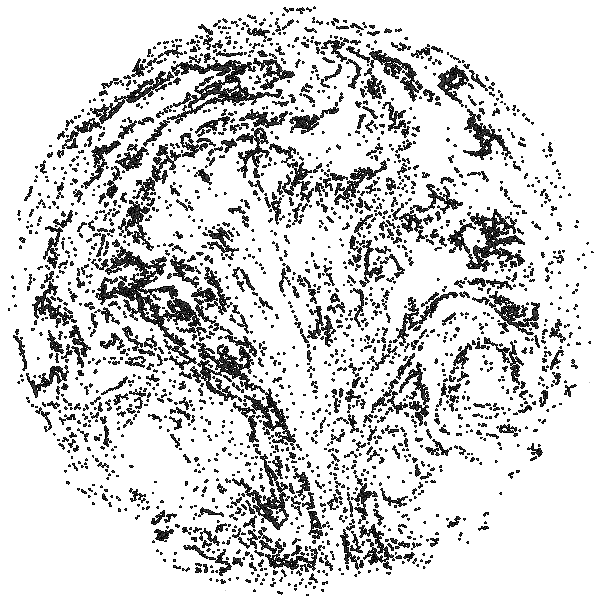

Supplement: S1 Fig — (ZIP) [file pone.0211413.s002.zip › Test images/S4_16.bmp]

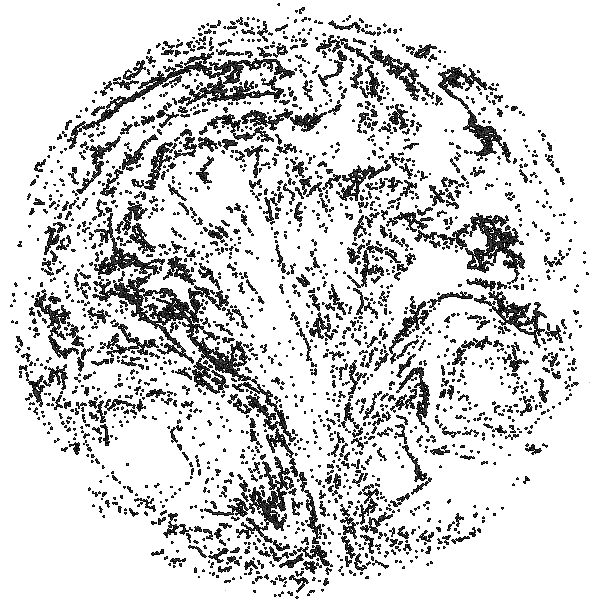

Supplement: S1 Fig — (ZIP) [file pone.0211413.s002.zip › Test images/S4_17.bmp]

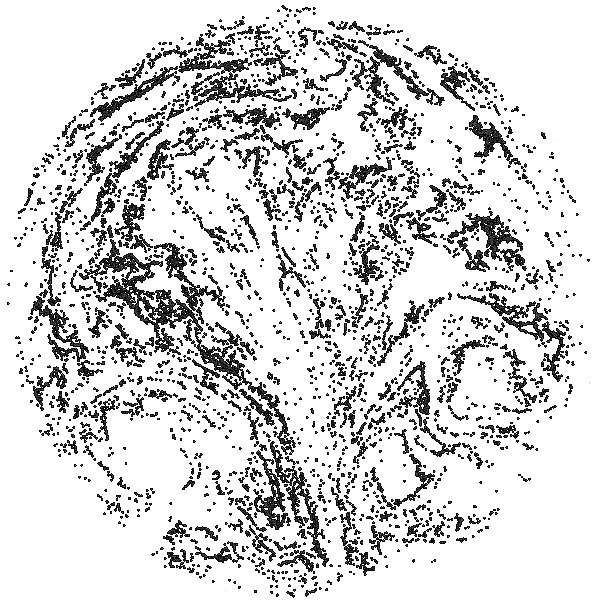

Supplement: S1 Fig — (ZIP) [file pone.0211413.s002.zip › Test images/S4_18.bmp]

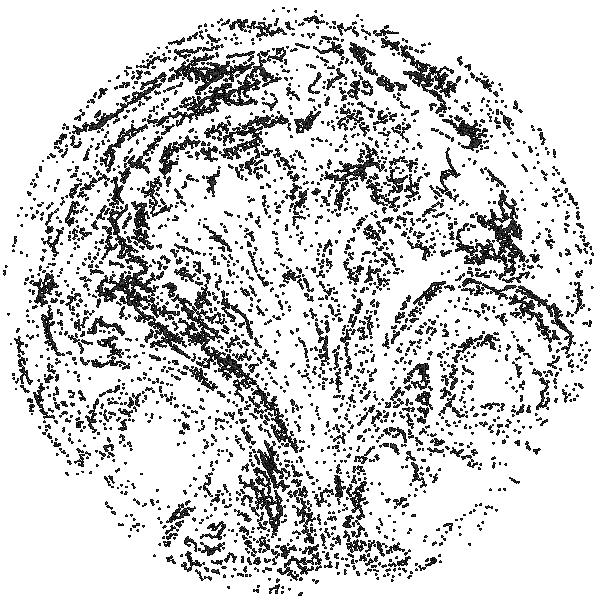

Supplement: S1 Fig — (ZIP) [file pone.0211413.s002.zip › Test images/S4_19.bmp]

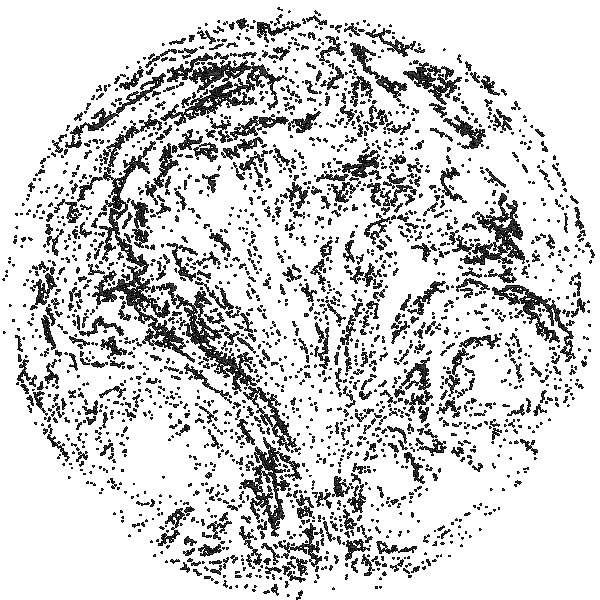

Supplement: S1 Fig — (ZIP) [file pone.0211413.s002.zip › Test images/S4_20.bmp]

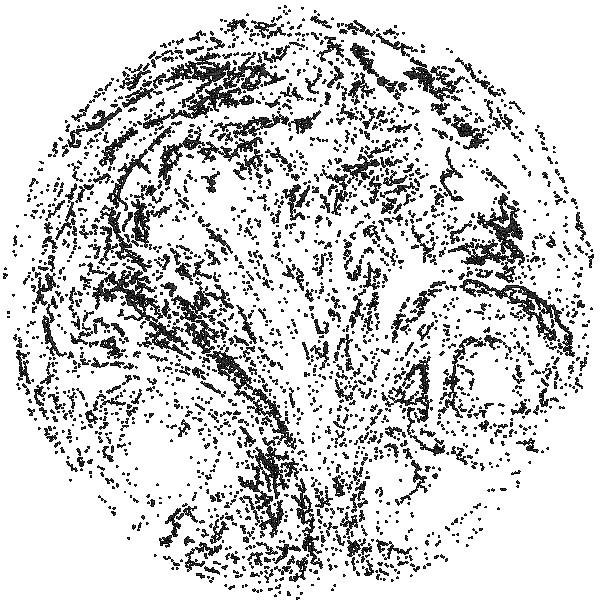

Supplement: S1 Fig — (ZIP) [file pone.0211413.s002.zip › Test images/S4_21.bmp]

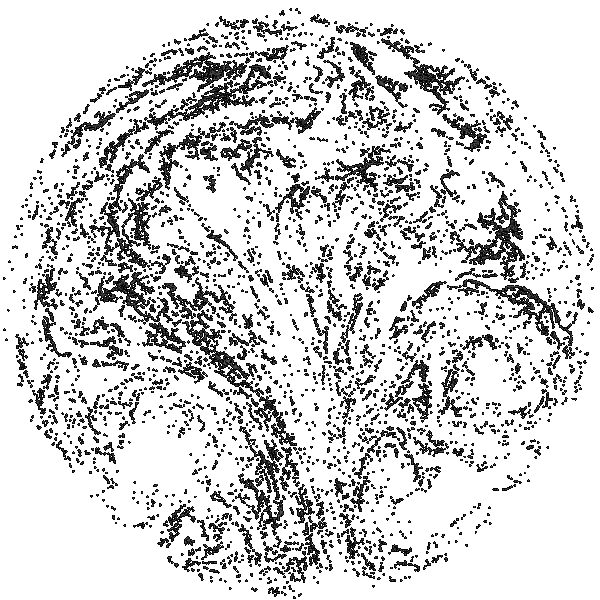

Supplement: S1 Fig — (ZIP) [file pone.0211413.s002.zip › Test images/S4_22.bmp]

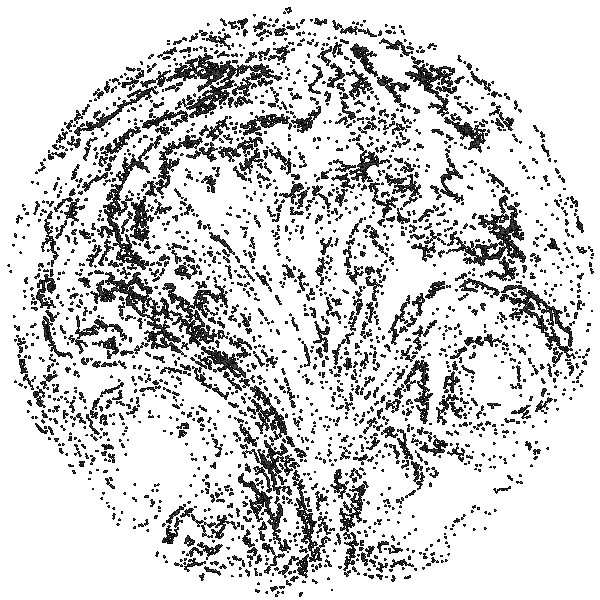

Supplement: S1 Fig — (ZIP) [file pone.0211413.s002.zip › Test images/S4_23.bmp]

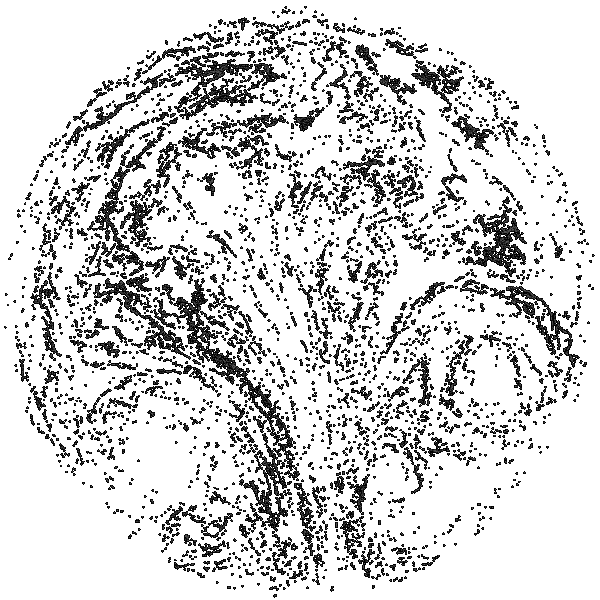

Supplement: S1 Fig — (ZIP) [file pone.0211413.s002.zip › Test images/S4_24.bmp]

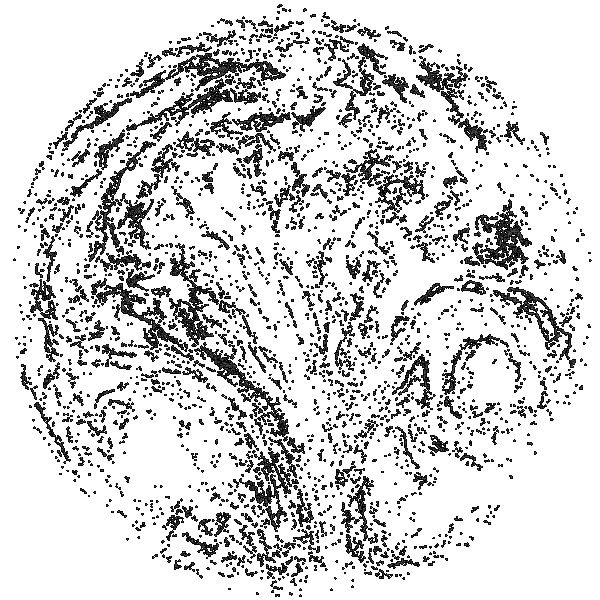

Supplement: S1 Fig — (ZIP) [file pone.0211413.s002.zip › Test images/S4_25.bmp]

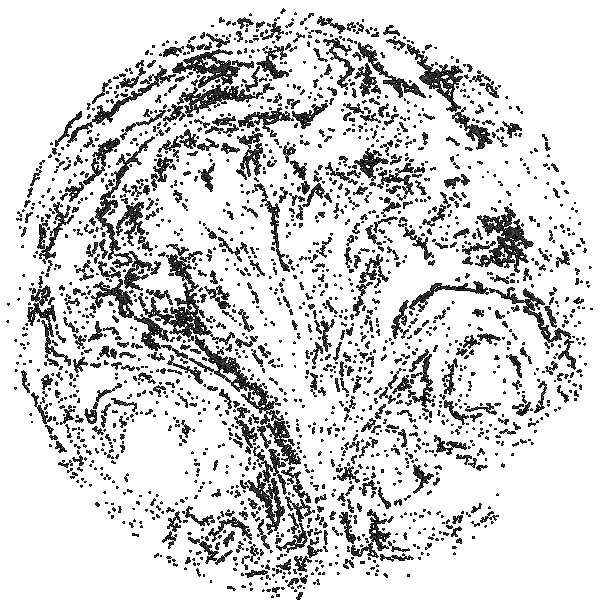

Supplement: S1 Fig — (ZIP) [file pone.0211413.s002.zip › Test images/S4_26.bmp]

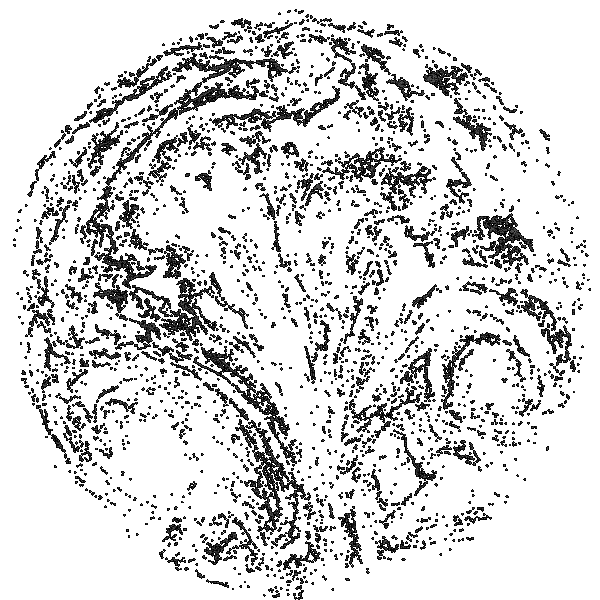

Supplement: S1 Fig — (ZIP) [file pone.0211413.s002.zip › Test images/S4_27.bmp]
